# Theoretical Analysis of Exciton Wave Packet Dynamics in Polaritonic Wires

**DOI:** 10.1021/acs.jpclett.3c01082

**Published:** 2023-06-14

**Authors:** Gustavo J. R. Aroeira, Kyle T. Kairys, Raphael F. Ribeiro

**Affiliations:** Department of Chemistry and Cherry Emerson Center for Scientific Computation, Emory University, Atlanta, Georgia 30322, United States

## Abstract

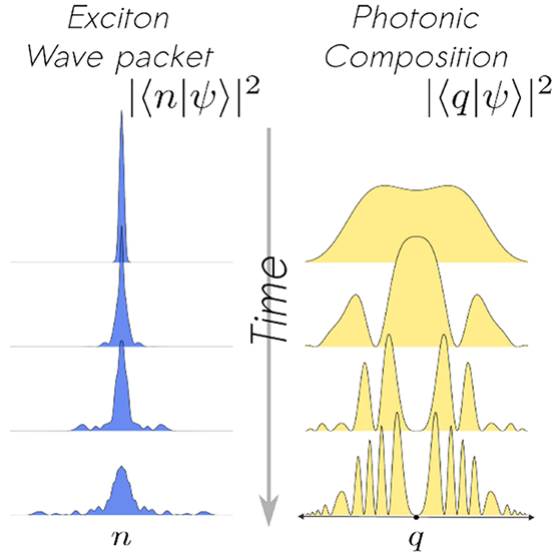

We present a comprehensive study of the exciton wave packet evolution in disordered lossless polaritonic wires. Our simulations reveal signatures of ballistic, diffusive, and subdiffusive exciton dynamics under strong light–matter coupling and identify the typical time scales associated with the transitions between these qualitatively distinct transport phenomena. We determine optimal truncations of the matter and radiation subsystems required for generating reliable time-dependent data from computational simulations at an affordable cost. The time evolution of the photonic part of the wave function reveals that many cavity modes contribute to the dynamics in a nontrivial fashion. Hence, a sizable number of photon modes is needed to describe exciton propagation with a reasonable accuracy. We find and discuss an intriguingly common lack of dominance of the photon mode on resonance with matter in both the presence and absence of disorder. We discuss the implications of our investigations for the development of theoretical models and analysis of experiments where coherent intermolecular energy transport and static disorder play an important role.

Interaction between light and matter is enhanced within optical microcavities and plasmonic devices due to the confinement of the electromagnetic (EM) field to a small region of space.^1^ These structures have been used to design landscapes where the strong light–matter coupling regime achieved enables the emergence of light–matter hybrid states commonly denoted (cavity) polaritons.^2,3^ The presence of these polaritonic states has been shown to modify energy transport,^4−11^ conductivity and photoconductivity,^12−16^ optical response,^3,17^ and chemical reactions.^9,17−20^ Hence, these devices have been not only objects of theoretical interest but also prospectus of new technology.^1,21,22^

Energy transfer mediated by microcavity polaritons was first verified with a binary J-aggregate mixture using photoluminescence^4^ followed by femtosecond transient spectroscopy.^23^ Even when donor and acceptor molecules were spatially separated, energy transfer was observed, ruling out the possibility of dipole–dipole energy transfer.^5^ Transport distances of approximately 20 μm have been reported for inorganic quantum well polaritons with low photon content in a two-dimensional planar cavity.^24^ In contrast, polaritons emergent from strong coupling of Bloch surface waves and organic materials showed  μm propagation lengths.^25,26^ Corresponding group velocities of over 120 μm ps^–1^ were also deduced from dispersion relations.^25^ Direct measurements of exciton–polariton pulse widths have recently revealed growth rates of less than 1 μm ps^–1^.^27^ Recent work has also shown unexpected dependence of the polariton wave packet width on the microcavity quality factor^28,29^ as well as the coexistence of diffusive and ballistic transport regimes controllable via the photonic content of the dominant wave packet component.^30^ Another key feature reported in recent work^30,31^ is the renormalization of the ballistic transport velocity and diffusion constants of exciton-polaritons induced by dynamical and static disorder.

Inspired by promising experimental findings, several theoretical studies have emerged aiming to clarify and optimize the mechanism underlying polariton-assisted transport.^32−42^ Models based on a one-dimensional chain of two-level systems coupled to a single cavity mode showed that the collective coupling between matter and the photonic material might overcome generic disorder-induced transport suppression.^32,33^ It has also been proposed that hopping and cavity-mediated energy transfer formed independent transport channels.^32,43^ The role of dark states, i.e., states with a small or zero photonic content, has also been examined. It was proposed that these states, unlike noninteracting matter states outside a microcavity, can be spatially delocalized and therefore contribute to efficient energy transport.^34,44^ In spite of these compelling insights, most theoretical work so far has been done using single-photon mode theories, where molecules inside the cavity are coupled to a single spatially homogeneous photon mode. Earlier work by Agranovich and Gartstein investigated polariton propagation along a one-dimensional multimode cavity establishing that disorder tends to localize polariton modes with nearly zero wave vector.^45^ The importance of a multimode description of the electromagnetic field has become clear in recent studies.^41−43,46,47^ For example, it has been shown that significantly different dynamics occurs in the presence of a more realistic radiation field including multiple degrees of freedom.^46,47^ Nevertheless, a quantitative analysis is still lacking on how predictions of dynamic exciton phenomena depend on the various choices that need to be made in the computational investigation of multimode polaritonic materials (e.g., finite system size and number of cavity modes). In addition, phenomenological questions remain open on the time scales associated with exciton transport phenomena such as ballistic, diffusive, subdiffusive (transient and long-time localization), and disorder-enhanced transport in optical cavities.

In this work, we analyze the coherent propagation of exciton wave packets in a lossless polaritonic wire to study multimode dynamics and numerical accuracy. We compute the time evolution of exciton wave packets and examine how the results depend on the system size and number of cavity modes. Next, we discuss which photon modes provide the dominant contribution to the exciton transport and how this property is affected by the initial state preparation, light–matter interaction strength, and static energetic disorder. Although energetic disorder introduces nontrivial dynamic effects, we are able to present a simple energy-based criterion to determine the most relevant set of photon modes. Finally, we discuss the most prominent dynamic features observed in our simulations. Since our model does not include dissipative effects, our conclusions can be seen as limiting or upper-bound expectations. Nevertheless, we anticipate the analysis presented here provides new insight into transient exciton evolution under strong light–matter coupling and recommendations for future theoretical model development.

The polaritonic wire model, illustrated in Figure 1, describes a perfectly reflective cuboid microcavity with confinement lengths of *L*_*x*_, *L*_*y*_, and *L*_*z*_, where *L*_*x*_ is the long dimension (*L*_*x*_ ≫ *L*_*z*_, *L*_*y*_). This ideal cavity confines the EM field along the *y* and *z* coordinates, while we impose periodic boundary conditions along the *x* direction. Matter is modeled by a chain of two-level systems, which can represent atoms or molecules with weak vibronic coupling over ultrafast times. The two-level systems are fixed at sites distributed along *x* with an average intersite distance *a*. In a system with *N*_*M*_ sites, the length along *x* is *L*_*x*_ = *N*_*M*_*a*, whereas *L*_*y*_ and *L*_*z*_ are fixed at 200 and 400 nm, respectively. The total Hamiltonian for this system can be separated into three components
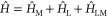
1representing matter , light , and their interaction .

**Figure 1:**
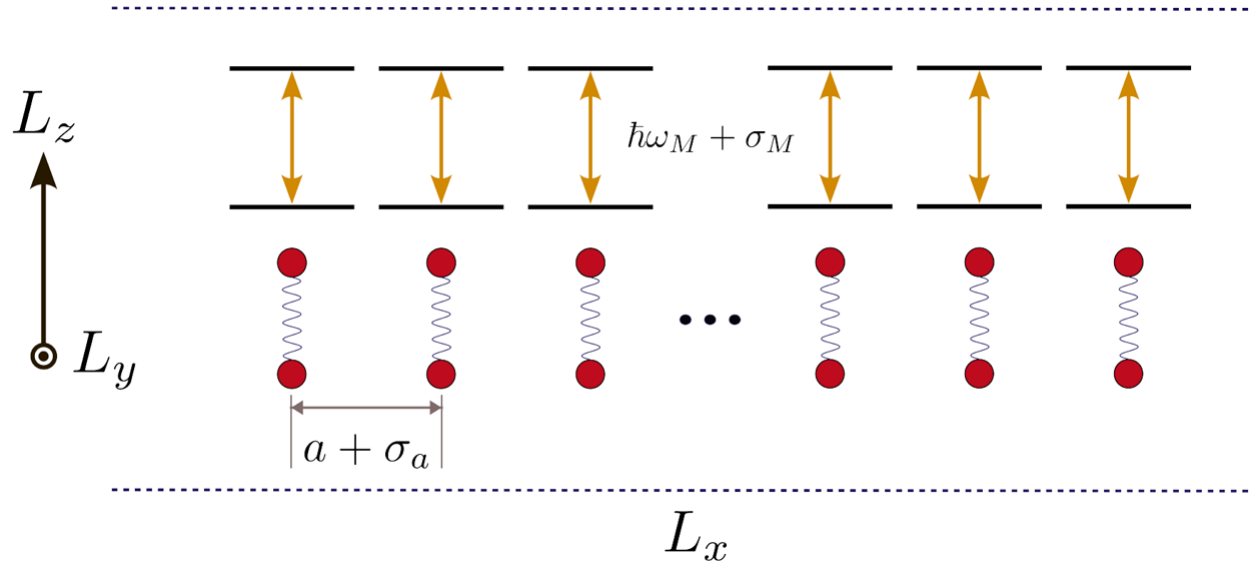
Model of a lossless cuboid microcavity (polaritonic wire) hosting *N*_M_ two-level systems fixed at sites along *x* representing matter. The distance between the sites (*a*) and the excitation energy (*E_M_= ℏω*_*M*_) of each two-level system includes disorder drawn from a normal distribution with corresponding standard deviations σ_*a*_ and σ_*M*_, respectively. Throughout this article, *L*_*y*_ = 200 nm and *L*_*z*_ = 400 nm are employed.

For the sake of simplicity, we assume that all two-level transition dipoles are parallel to the transverse-electric radiation modes of the photonic wire. The cavity radiation field satisfies periodic boundary conditions along *x* and Dirichlet conditions along *y* and *z*. Consequently, the allowed wave vectors are characterized by three quantum numbers
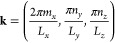
2where  and . Since the quantization length is small along *y* and *z*, the energy gap between different values of *n*_*y*_ and *n*_*z*_ is large. Hence, we consider only the lowest-energy band, i.e., *n*_*y*_ = *n*_*z*_ = 1. Defining the quantities
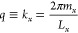
3
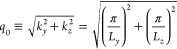
4we can fully describe each cavity mode by its value of *q*. The Hamiltonian of the EM field can be written as
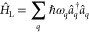
5
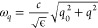
6where *ℏ* is the reduced Planck constant, *c* is the speed of light, ϵ is the relative static permittivity, and  and *â* are bosonic creation and annihilation operators, respectively. The sum over cavity modes in eq 5 is, in principle, infinite. However, the importance of a particular mode becomes negligible as it grows highly off-resonant with the transitions of interest. Therefore, the number of cavity modes (*N*_*c*_) is truncated by choosing a cutoff value . In general, the *N*_*c*_ modes include positive and negative *q* values representing counterclockwise and clockwise waves, but we also consider the effect of retaining only one direction. The relative permittivity is chosen as ϵ = 3, compatible with organic microcavities, and combined with the chosen wire geometry, it yields a minimum cavity energy *ℏω*_*q*=0_ = 2.00 eV.

Each site along the wire is a two-level system with the mean excitation energy *ℏω*_*M*_ and an average intersite distance of *a*. Static disorder is introduced in the system by allowing the two-level energy gap and positions to vary following the normal distributions
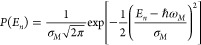
7
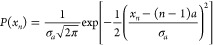
8where *E*_*n*_ and *x*_*n*_ are the energy and position of the *n*th site, respectively. The standard deviations σ_*M*_ and σ_*a*_ characterize the static disorder of the system. We assume intersite interactions are weak enough that any bare energy transfer occurs on a much longer time scale than probed by our simulations and, hence, can be ignored. The matter component of the Hamiltonian is written simply as
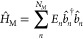
9with *E*_*n*_ sampled from eq 7 and  representing an operator that promotes (demotes) the *n*th two-level system to (from) its excited state (i.e., ).

Employing the Coulomb gauge in the rotating wave approximation (RWA) and neglecting the diamagnetic contribution, the interaction between light and matter can be written in terms of the collective light–matter interaction strength (Rabi splitting, Ω_*R*_) as follows:
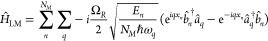
10Since , choosing a value of Ω_*R*_ and density (ρ = *N*_*M*_/*L*_*x*_*L*_*y*_*L*_*z*_) implies a transition dipole moment (μ). We assume that all two-level transition dipoles are aligned with the polarization of the cavity modes; therefore, different interactions between matter and photons arise only from their relative energy and the varying electric field amplitude along the wire for |*q*| > 0. Since this Hamiltonian relies on the electric dipole approximation, we numerically verified that even when very high-energy photons are considered, the wavelength remains greater than the spacing between sites. Furthermore, as our results will show, these highly off-resonant modes are negligible to exciton transport.

The initial states of the simulations consist of purely material exciton Gaussian wave packets placed in the center of the wire (*N*_*M*_*a*/2). In the uncoupled basis, these wave packets can be represented as
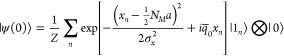
11where *Z* is a normalization constant, σ_*x*_ is the initial spread of the wave packet (eq 13), and |1_*n*_⟩ ⊗ |0⟩ represents a state where the *n*th site is in its excited state while all other sites and cavity modes are in the ground state. The mean initial exciton momentum is given by  and, unless otherwise noted, it is taken as zero in all simulations. The wave packet dynamics is obtained by first diagonalizing the Hamiltonian matrix (eq 1) and constructing the time-evolved wave packet exactly as .

We investigate the dynamics within the one-excitation manifold; that is, the Fock-space is truncated to include photon modes with either 0 or 1 photons. Therefore, our results are mostly relevant in the dilute limit (weak pumping) scenario where the exciton density is small and nonlinearities can be ignored. Note that in disordered microcavities, nearly pure matter initial states can be constructed via resonant excitation of weakly coupled modes.

To obtain a measure of the number of sites over which the wave packet extends, we compute the wave packet width *d*(*t*) defined as the root-mean-square displacement divided by the average intersite distance
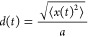
12
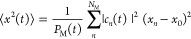
13
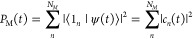
14where *c*_*n*_(*t*) = ⟨1_*n*_|ψ(*t*)⟩ are the local site amplitudes and *P*_M_(*t*) is included as normalization factor in eq 13 so the exciton wave packet width is computed from the conditional probability of finding the exciton on a given site.

All computations were performed using our prototype package PolaritonicSystems.jl.^48^ Double-precision complex numbers were used for representing the state vectors. Matrix operations were carried out with Intel MKL and the LAPLACK backend. Random numbers sampled from normal distributions were generated using the Distributions.jl package.^49^ Figures were produced using the Makie.jl plotting ecosystem.^50^

In most experiments, the collective strong coupling regime is achieved using a macroscopic number of molecules in optical microcavities.^3,51^ For the sake of computational feasibility, simulations are performed on a much smaller system size. To assess how this reduction affects exciton dynamics, we simulated the propagation of wave packets at various system sizes in the absence of disorder. The results presented in Figure 2 were obtained by varying the number of sites (*N*_*M*_) and, consequently, the wire length (*L*_*x*_ = *N*_*M*_*a*). This scheme maintains the density of the system constant, allowing us to fix the Rabi splitting at 0.1 eV. The large number of photon modes employed here (*N*_*c*_ = 1601) was chosen to ensure convergence for all system sizes; the sensitivity of our results with respect to this parameter is explored in the following section. The evolution of the wave packet width *d*(*t*) for multiple system sizes is shown for short and long propagation times in Figure 2a,b, respectively, while snapshots of wave packets at selected times are shown in Figure 2c.

**Figure 2:**
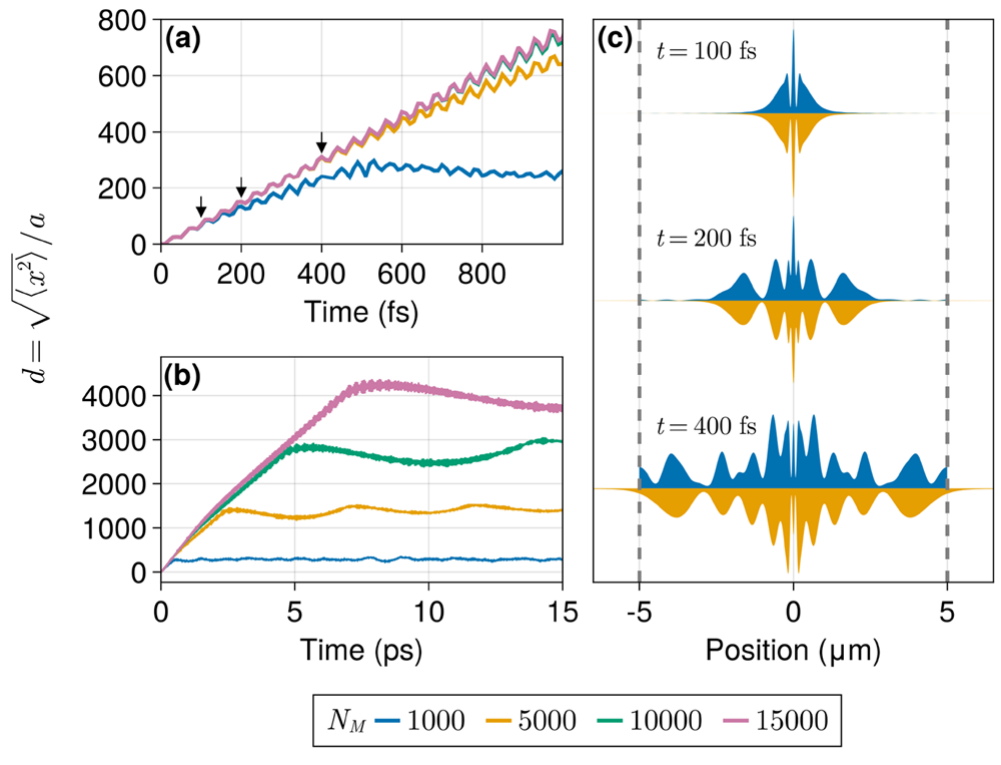
Wave packet width (*d*) over short (a) and long (b) propagation times for several system sizes with no disorder. (c) Exciton wave packet shapes (arbitrary scale) for *N*_*M*_ = 1000 and 5000 at selected time steps, indicated by arrows in panel a. Dashed lines indicate the same point in the *N*_*M*_ = 1000 (circular) wire. The radiation field was modeled by using 1601 cavity modes, and the Rabi splitting was set to 0.1 eV. Sites are positioned 10 nm apart from each other. The lowest cavity mode is in resonance with the two-level energy gap (*ℏω*_*M*_) of 2.0 eV. The initial wave packet was prepared with σ_*x*_ = 60 nm (*d*(0) = 6 sites). Cavity cutoff energies are 57.30, 11.63, 6.07, 4.31, and 3.49 eV, in increasing order of *N*_*M*_.

In every examined case, we see a linear ballistic-like transport with small-amplitude oscillations around the main profile. These oscillations have a period compatible with the corresponding Rabi splitting, indicating that they arise due to periodic exchange of energy between light and matter. At longer times, the exciton width reaches a plateau, around which it oscillates. This apparent localization is a finite system size effect, as in the absence of disorder the wave packet motion in an infinite system is unbounded. At propagation times under 200 fs, the same time-linear profile is found for all selected system sizes. The worst case is seen for *N*_*M*_ = 1000 where the exciton slows down significantly as the wave packet extends over all sites within approximately 400 fs, as shown in Figure 2c. Note that different system sizes will imply different photon densities of states, which may be a source of error. Nevertheless, our computed values of *d* and wave packet shapes are well in agreement for all different sizes up to localization due to finite size. Since disorder induces wave packet localization,^52,53^ we expect it will lessen the finite-size effects responsible for the deviations observed in Figure 2. Therefore, these results suggest that transport properties can be probed with a small number of sites as long as the system is larger than the exciton localization length scale or the probing time is earlier than the localization time scale.

Our next discussion concerns the quantitative convergence of results with respect to the set of cavity modes employed. Although recent work has highlighted the importance of a multimode description of the cavity radiation field,^43,46,47^ as far as we are aware, a quantitative analysis of how the accuracy of material observables depends on the number of cavity modes has not yet been presented. This discussion is important for both theoretical models and the interpretation of experimental results since, as we will show, qualitatively incorrect results are obtained when too few photon modes are considered. To assess the convergence of the exciton dynamics with respect to the number of photon modes (*N*_*c*_), we compute the time-dependent exciton width (eq 12) using different cavity cutoffs *q*_max_ (eq 6). For any choice of cutoff, all modes with *q* satisfying |*q*| < *q*_max_ are retained. A subsequent section examines the consequences of neglecting *q* < 0 modes. We employ the following global (integral) measure of wave packet propagation error due to cavity mode truncation:
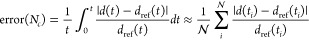
15where *d*_ref_ is taken from a computation using a large number of photon modes (*N*_*c*_ = 1601) sufficient to achieve converged dynamics. In Figure 3a, we show that, without disorder, in the best case scenario with *N*_*M*_ = 5000, a large *N*_*c*_ > 200 is necessary to reduce the numerical error measured by eq 15 substantially. An increase in the size of the system leads to a larger error at fixed *N*_*c*_ and slower convergence toward the exact result as *N*_*c*_ increases. This happens because the photon energy spacing  decreases with the system length (eqs 3–6). For instance, when *N*_*M*_ = 5000 the lowest 201 modes span 0.46 eV, while simulations with *N*_*M*_ = 10^4^ and *N*_*M*_ = 2 × 10^4^ require 401 and 801 modes, respectively, to span the same energy range. When the truncation errors are presented as a function of the photon energy cutoff (Figure 3b), a general trend emerges, where we observe an exponential decay of the error with respect to the cutoff value. Hence, the energy range spanned by the included photon modes is the most relevant parameter of the truncation. Figure 3c shows the necessary upper energy cutoff to keep the truncation error below 0.01 as a function of Ω_*R*_. We found that detuning (redshifting the cavity) had little effect on this trend. However, the initial state spread affected the result significantly. In the worst case scenario, for a narrow initial wave packet (σ_*x*_ = 60 nm), we found that the required photon energy cutoff in the absence of disorder scales linearly as 2Ω_*R*_. This factor can serve as a heuristic relationship to estimate the range of important photon modes. The fact that the error vanishes for a sufficiently large number of photon modes demonstrate the convergence of our model. Recent work has shown that this is not always guaranteed. Mandal et al.^54^ reported that using the dipole gauge and RWA, polaritonic dispersion relations do not converge with respect to the number of cavity modes unless the often neglected dipole self-energy terms are included in the model.^55,56^

**Figure 3:**
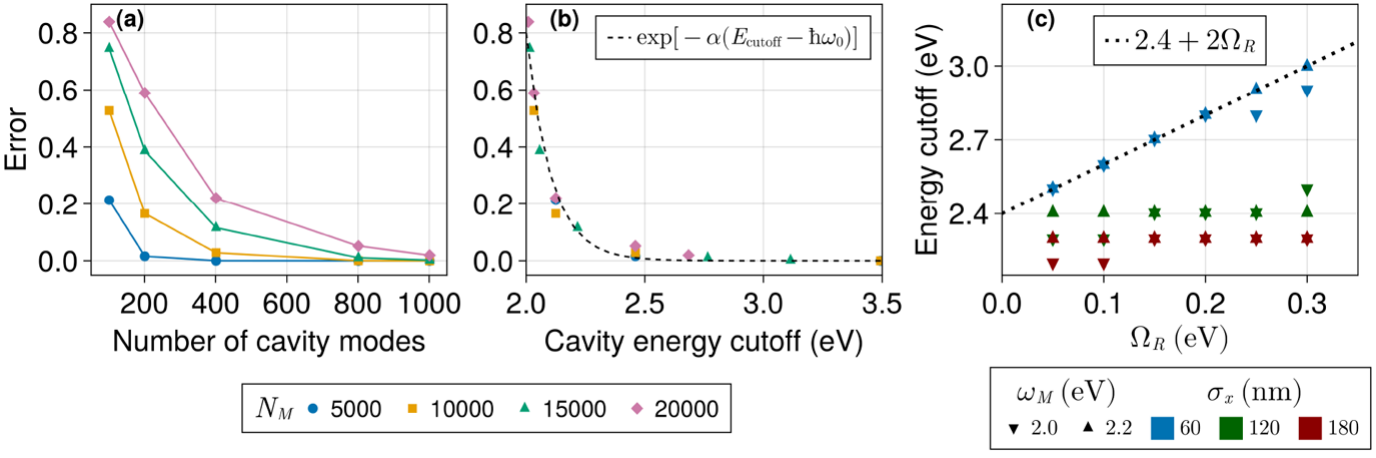
Error in the wave packet width (eq 15) without disorder for several system sizes as a function of the number of cavity modes (a) and the energy cutoff value (b). The error was computed over an interval of 5 ps with time steps of 10 fs. Rabi splitting (Ω_*R*_) was set to 0.1 eV. Sites are positioned 10 nm apart from each other. The lowest cavity mode is in resonance with the two-level energy gap (ω_*M*_) of 2.0 eV. The initial wave packet was prepared with σ_*x*_ = 60 nm (*d*(0) = 6 sites). (c) Estimated energy cutoff necessary for a truncation error of less than 0.01 as a function of Ω_*R*_ for several values of σ_*x*_ and ω_*M*_.

Next, we will consider how static fluctuations in the energy gaps of the two-level systems change the results presented in Figure 3. Static disorder induces wave function localization^45,51,52^ and potentially reduces the total photon content of the wave packet, e.g., for an exciton with σ_*x*_ = 120 nm and Ω_*R*_ = 0.1 eV, the average photon content drops from 40% to around 20% when the energetic disorder is increased from 0.005 to 0.02 eV (see Figure S16). At the same time, exciton propagation in a disordered landscape is irregular, involving many scattering events that might require a more flexible description of the field that includes many degrees of freedom. In light of these considerations and the fact that disorder is an unavoidable feature of polaritonic materials, it is important to determine how the introduction of static disorder changes the accuracy of simulations performed with a finite number of cavity modes.

Our model includes energetic and positional disorder as described in eqs 7 and 8. As reported in earlier work,^47,57,58^ even small energetic disorder dominates over translational disorder. Therefore, we fix the site position standard deviation to be σ_*a*_ = 1 nm in all calculations that include disorder. To assess the effects of field truncation, we set Ω_*R*_ = 0.1 eV, σ_*x*_ = 60 nm, and *a* = 10 nm as representative examples along with *N*_*M*_ = 5000 following our previous discussion of finite size effects. The truncation error is computed from eq 15 by using the average value of *d*(*t*) obtained from 100 realizations for each probed *N*_*c*_ and σ_*M*_. The computed errors’ uncertainty was obtained using linear propagation theory implemented in the Measurements.jl package.^59^

The average exciton *d*(*t*) profiles obtained with several *N*_*c*_ values when the energetic disorder σ_*M*_ is equal to 20% and 50% of Ω_*R*_ are presented in Figure 4a,b, respectively. The shaded region covers twice the standard deviation of *d*_ref_ in both cases. The error analysis is more complex in the presence of disorder due to the stochastic nature of the system. Nonetheless, we can clearly distinguish mean trajectories that are qualitatively different from the reference. For instance, average trajectories with *N*_*c*_ = 1 and *N*_*c*_ = 21 exhibit significant deviation from the reference, indicating the qualitative incorrectness of the corresponding incomplete mode expansion. The mean *d*(*t*) obtained with *N*_*c*_ = 151 lies just above our error threshold of 0.05 and, for the most part, is contained within two standard deviations of *d*_ref_(*t*). Errors as a function of cavity energy cutoffs for several disorder strengths are shown in Figure 4c. In spite of some complicated features at inadequately low cutoff energies, the overall trend is not too different from that observed in the absence of disorder (Figure 2). Therefore, we believe the results obtained in the absence of disorder can be used to reliably estimate the number of cavity modes needed for a particular system.

**Figure 4:**
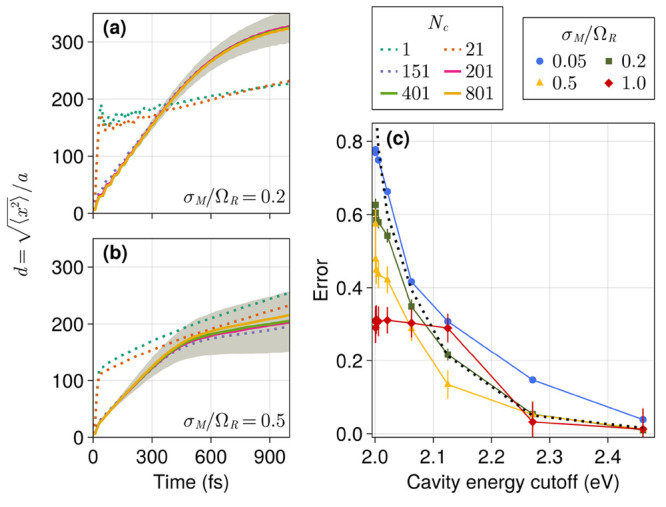
Wave packet width, *d*(*t*), over time using different numbers of cavity modes (*N*_*c*_) with relative disorder strength σ_*M*_/Ω_*R*_ = 0.2 (a) and σ_*M*_/Ω_*R*_ = 0.5 (b). Energy cutoffs are 2.00, 2.01, 2.27, 2.46, 3.49, and 6.07 eV, in increasing order of *N*_*c*_. Dotted lines highlight trajectories with errors above 0.05. (c) Error in the wave packet width (eq 15) for several energetic disorder values and Ω_*R*_ = 0.1 eV. The dotted black line shows the convergence observed without disorder. The lowest cavity mode is in resonance with an average two-level energy gap (ω_*M*_) of 2.0 eV. The wire contains 5000 sites with *a* = 10 and σ_*a*_ = 1 nm. The initial wave packet was prepared with σ_*x*_ = 60 nm (*d*(0) = 6 sites). The simulation was run for 1 ps with time steps of 10 fs. Values shown are averages over 100 realizations, and error bars are propagated from twice the standard deviation of *d*.

In the results discussed so far in this Letter, we found that quantitative converged results require many photon modes. This indicates that a broad energy range of photons modes  contributes to exciton propagation, which is at odds with the intuition that resonant processes must dominate the dynamics over sufficiently long times. To further understand these findings, we inspect the photonic composition of the wavepacket over time. This will give us insight into the state of the radiation within the cavity, which is inaccessible with single-mode models. Moreover, we discuss how this photonic probability distribution changes with experimentally controllable variables. To obtain this profile, we compute the time-averaged relative mode weight distribution, defined as
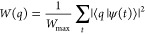
16where  represents a state with one photon with wave vector *q* and no excited sites, and *W*_max_ is equal to max_*q*_*∑*_*t*_|⟨*q*|ψ(*t*)⟩|^2^. The discrete sum over time (*t*) was performed numerically using time increments of 5 fs for a total period of 5 ps.

In Figure 5, we present *W*(*q*) obtained in the absence of disorder for various Ω_*R*_ and variable initial exciton widths *d*(0) with *N*_*m*_ = 5000 and *N*_*c*_ = 401. In this subsection, we work with variable values of Ω_*R*_ and σ_*x*_ because these are experimentally tunable quantities that significantly affect the time-averaged relative photon weight distribution. The mean two-level energy gap was set to 2.2 eV (resonant with the photon modes *q*_*r*_ such that ). We probed the time-averaged photon weight distributions for excitons with both vanishing and nonvanishing initial wave vectors ( and , respectively).

**Figure 5:**
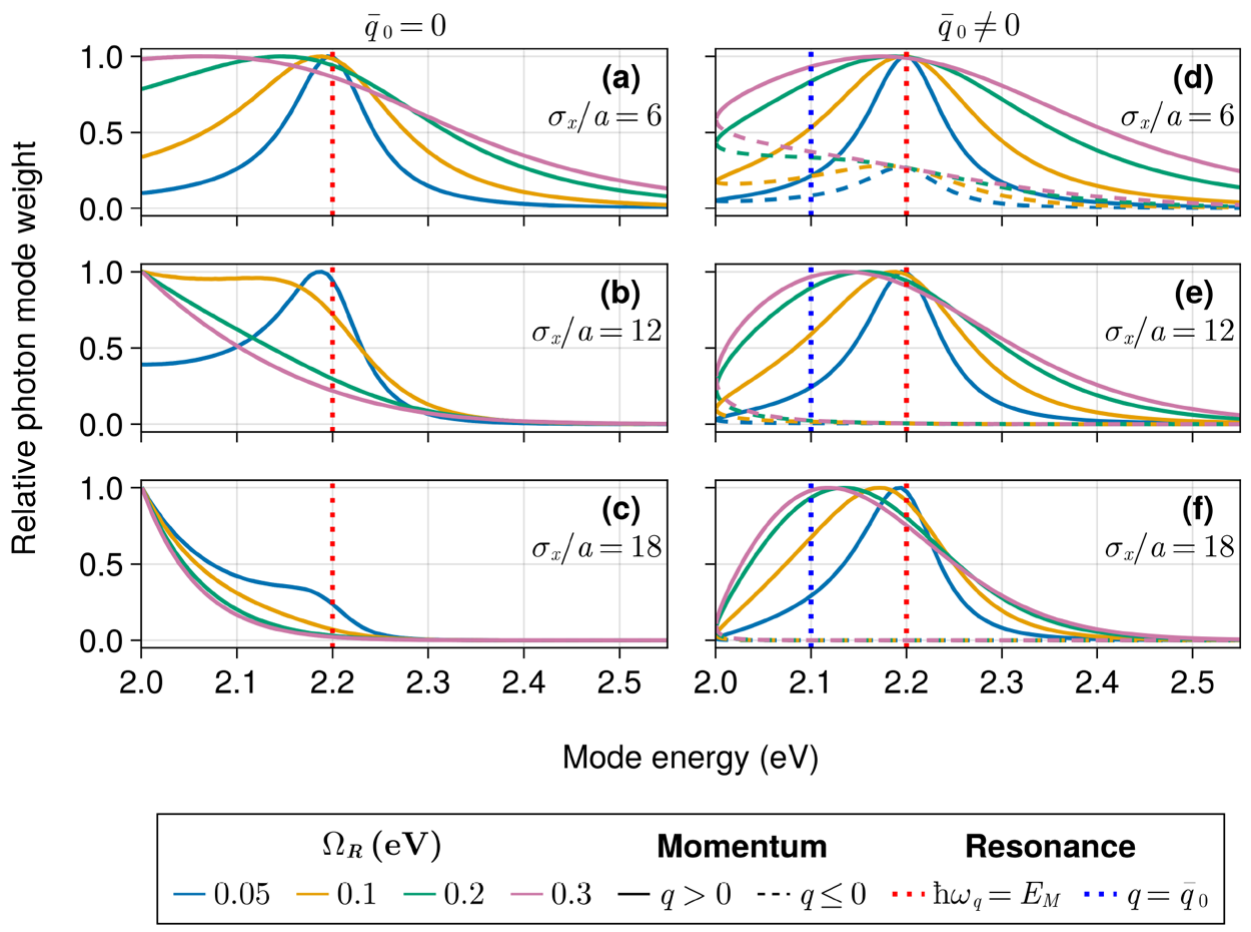
Cavity mode contribution measured by eq 16 under no disorder. The computation was performed over 5 ps using a 5 fs time step. The number of sites and cavity modes is set to 5000 and 401, respectively. The distance between sites (*a*) is fixed at 10 nm, and the sites’ two-level excitation energy is 2.2 eV, indicated by the dotted red line. The mean exciton momentum  is set to zero (a–c) or 0.00565 nm^–1^ (d–f), which matches the momentum of the photon at 2.1 eV (dotted blue line). Modes with *q* > 0 and *q* ≤ 0 are represented by solid and dashed lines, respectively.

The distinct choices of cavity–matter detuning (*E*_0_ – *E*_*M*_ = −0.2 eV) and mean initial exciton wave vector examined in this section relative to the prior will allow us to reveal an interesting interplay between the competing biases of the photon weight distribution toward distinct wave vectors *q* satisfying *q* = *q*_*r*_ (energy resonance) and  (quasimomentum matching). The competition between *q*_*r*_ and  can be demonstrated analytically in the infinite system limit where *L*_*x*_ → *∞* and simultaneously *N*_*M*_ = *N*_*c*_ → *∞*. In this case, for a system without disorder, the time-average photon mode probability distribution generated by an initial exciton Gaussian wave packet in a polaritonic wire can be approximated by (see the Supporting Information for derivation)
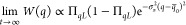
17where Π_*qL*_ is total matter content of the lower polariton mode with wave vector *q*. Note that *W*(*q*) is proportional to the product of two competing terms. The exponential factor favors the photon modes with the same mean quasimomentum  as the initial wave packet with typical fluctuations of size 1/σ_*x*_. However, the bias toward the initial momentum might be irrelevant if the prefactor Π_*qL*_(1 – Π_*qL*_) is very small at . In fact, this prefactor is maximized when , which happens at resonance *q* = *q*_*r*_. It follows that, when  and , the prefactor Π_*qL*_(1 – Π_*qL*_) approaches zero near ; therefore, *W*(*q*) will be maximized around *q* = *q*_*r*_. In the opposite limit, where , Π_*qL*_(1 – Π_*qL*_) is appreciable and varies slowly around , causing *W*(*q*) to be maximized at . In summary, the energy resonance condition (*q* = *q*_*r*_) will dominate when the Rabi splitting is not too large and the exciton is compact. Conversely, if the Rabi splitting is large enough, quasimomentum matching  is expected to determine the most important photon modes.

The photon weights shown in Figure 5 reflect the observations provided above based on eq 17 while also revealing a wide distribution of off-resonant modes (relative to the two-level excitation energy) contributing significantly to the overall dynamics. For instance, increasing Ω_*R*_ leads to a broader photon weight distribution with the corresponding peaks shifting toward the modes with  (2.0 eV when  and 2.1 eV when ). Notably, in every case, for sufficiently large Ω_*R*_ the maximum of the photon weight distribution matches the mean exciton wave vector, while the cavity modes in resonance with the matter system play a lesser role in the dynamics. Correspondingly, we see that when the exciton possesses a nonvanishing initial momentum , the contribution of photons with *q* < 0 is strongly suppressed. Further, the width of the photon weight distribution in *q* space decreases when the initial state becomes more delocalized, as seen when comparing panels d and f in Figure 5. This feature is in accordance with eq 17 and stems from the uncertainty principle. The results presented in this section also allow us to evaluate the validity of our two-level approximation for matter. Consider, for example, a three-level system with well-separated transition energies *E*_0→1_ = 2.0 eV and *E*_0→2_ = 3.0 eV. From Figure 5, we see that the probability of finding photons with energy greater than 2.5 eV is generally very small. Hence, another excited state centered around 3.0 eV would not interact significantly with any of the populated photon modes and, therefore, would have very limited participation in the overall process.

In the presence of disorder, eq 17 does not hold, and the total (photon + exciton) wave packet quasimomentum is not conserved anymore. The nonsingular distribution of site transition energies implies that a range of photon modes will be nearly resonant with the matter system, leading to a broader and more complex photon weight distribution. If the disorder is small enough, e.g., σ_*M*_ = 0.005 eV, the *W*(*q*) profiles are in good agreement with the previous discussion (see Figure S19a–c).

Photon weight distributions under stronger disorder are shown in Figure 6 for a wave packet with an initial width σ_*x*_/*a* = 12 and . At the smallest relative disorder strength, σ_*M*_/Ω_*R*_ = 0.1 (Figure 6a), we observe similar features with only slight changes relative to those of Figure 5d–f, specifically the competition between the resonant quasimomentum *q*_*r*_ and the initial exciton wave vector  leading to an average photon weight maximum at  (Figure 6a). Similarly, the photon modes with *q* < 0 are suppressed relative to *q* > 0 even when σ_*M*_ = 0.02 eV and Ω_*R*_ = 0.05 eV (Figure 6c). However, when the energetic disorder is increased to 0.05 eV, we find nearly equal weights for positive and negative photon wave vectors at all examined values of Ω_*R*_ (Figure 6d–f). This can be explained by the strong scattering induced by the disorder potential on the wave packet, which randomizes its *W*(*q*) distribution. Moreover, the enhancement of coherent backscattering, which is a generic feature of the propagation of waves in disordered media,^60^ favors a *W*(*q*) distribution that is symmetric with respect to the inversion of *q*.

**Figure 6:**
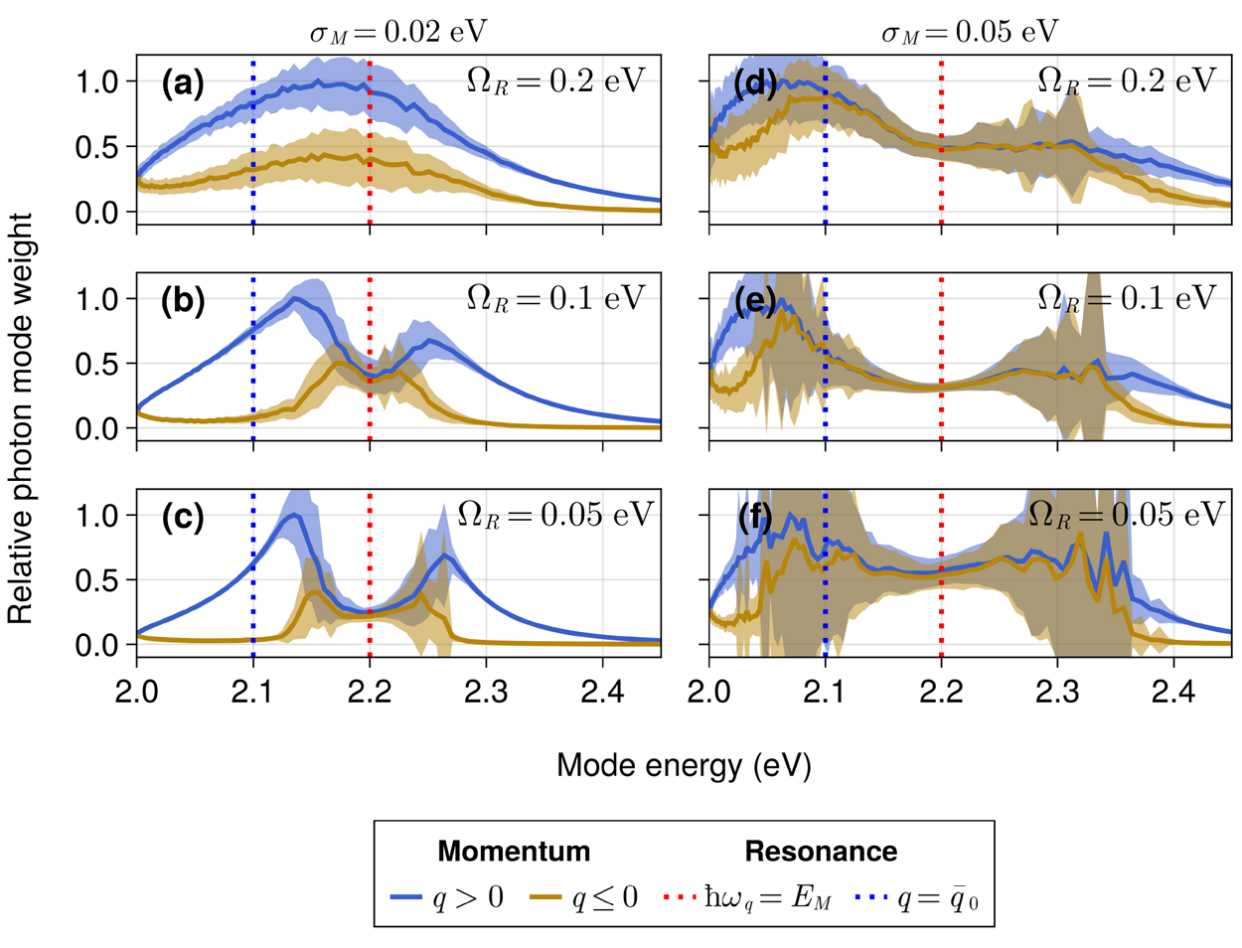
Cavity mode contribution measured by eq 16 under energetic disorder of σ_*M*_ = 0.02 eV (a–c) and σ_*M*_ = 0.05 eV (d–f). The wire was modeled with 401 cavity modes and 5000 sites with separation distances drawn from eq 8 using *a* = 10 and σ_*a*_ = 1 nm. The average site two-level excitation energy is 2.2 eV (dotted red line). The initial wave packet was prepared with σ_*x*_ = 120 nm (*d*(0) ≈ 12 sites) and an effective exciton momentum  0.00565 nm^–1^, which matches the momentum of the photon at 2.1 eV (dotted blue line). Modes with *q* > 0 and *q* ≤ 0 are colored blue and yellow, respectively. The computation was performed over five ps using a five fs time step. Band plots cover one standard deviation around the average values of 100 realizations.

The most remarkable feature observed in Figure 6 is the relative suppression of the photon modes nearly resonant with the center of the two-level excitation energy distribution. Specifically, a dip at *q* = *q*_*r*_ (*ℏω*_*q*_ = 2.2 eV) can be clearly observed when σ_*M*_/Ω_*R*_ is greater than 10% (Figure 6b–e) and begins to fade at stronger disorder σ_*M*_/Ω_*R*_ = 1 (Figure 6f). In general, the time-average photon weight can be expressed as *W*(*q*) ∝ *∑*_χ_|⟨*q*|χ⟩⟨χ|ψ(0)⟩|^2^, where the sum is over all eigenstates χ of the light–matter Hamiltonian (see the Supporting Information). This means that a photon mode will have a large weight if it can overlap with eigenstates (χ) that contribute significantly to the initial state (ψ(0)). A possible explanation for the increase in *W*(*q* < *q*_*r*_), especially with σ_*M*_ = 0.05 eV, may come from the fact that low |*q*| photon modes provide a greater contribution to localized polaritons.^45,51,57,58^ In turn, these localized polaritons might have greater overlap with the exciton initial state than the eigenstates with significant |*q*| ≫ 0 photon content. Future work will address this and other hypotheses for the *W*(*q* > *q*_*r*_) case.

Another interesting feature observed in Figure 6d–f is the greater standard deviation observed for *q* > *q*_*r*_ and *q* < *q*_*r*_ relative to *q*_*r*_. This is likely a byproduct of the greater fluctuations in both the number of sites with  and the light–matter matrix elements  (for the interaction between site *n* and cavity mode *q*) which arises in simulations with static energetic disorder.

Overall, disorder significantly decreases the dominance of photon modes with a particular wave vector or energy, leading to a flatter photon weight profile relative to that in the absence of disorder. This emphasizes the importance of utilizing a flexible and unbiased description of radiation as disorder makes the choice of photon modes less obvious. Nevertheless, in agreement with the nondisordered results discussed earlier, highly off-resonant modes (*ℏω*_*q*_ > 2.4 eV + 2Ω_*R*_) were observed to have negligible weight on the dynamics, further validating our energy cutoff criterion.

In the previous section, we investigated the convergence of our results with respect to the number of cavity modes in order to find the optimal set of photonic modes required to model exciton wave packet dynamics in photonic wires accurately. Another possible strategy to minimize the complexity of our model is to remove the double degeneracy of the cavity modes and retain only photons with *q* ≥ 0. We now briefly discuss the practical consequences of this alternative mode truncation approach.

Figure 7 compares exciton wave packets obtained in simulations that retained only *q* > 0 cavity modes (right panel) to wave packets obtained in the presence of positive and negative quasi-momentum modes (left panel) at different times in the presence of static disorder. The complete model has the wave packet spreading in both directions symmetrically. In contrast, the second model containing only *q* > 0 shows a suppressed transport toward the left side of the wire. Since the energy transport in our model is solely mediated by photons, this result can be readily interpreted to arise from the lack of left-moving photons in the simulations that retained only *q* > 0 modes.

**Figure 7:**
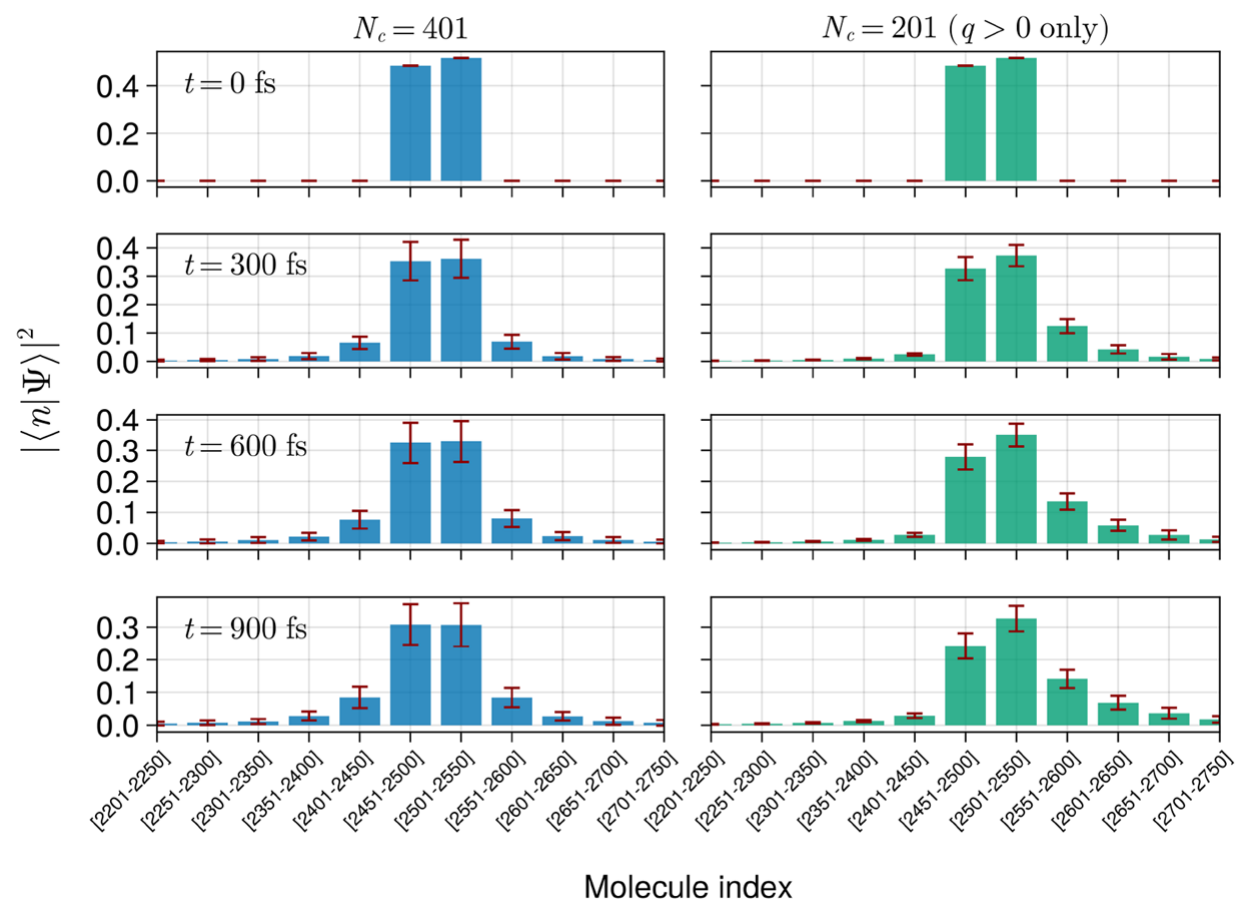
Wave packet grouped in bars over 50 sites for a system with positive and negative momentum photon modes (blue) and a system with only positive momentum photon modes (green). The number of sites in this wire is 5000, and Ω_*R*_ = 0.1 eV. The matter two-level system parameters are *E*_*M*_ = 2.0 eV, σ_*M*_ = 0.04 eV, *a* = 10 nm, and σ_*a*_ = 1 nm. The initial wave packets had a width of 120 nm (*d*(0) ≈ 12 sites). Error bars show the standard deviation obtained from 100 realizations.

We conclude that while an effective model that excludes photon modes traveling along a specific direction may be useful for the investigation of nonreciprocal systems with unidirectional energy transport, their predictions for coherent exciton mobility will likely be drastically overestimated in the presence of disorder due to the lack of backscattering processes. For instance, comparing the complete model (with doubly degenerate cavity modes) with the one-directional model, we find that the probability of detecting an excited site with *x*_*n*_ > *x*_0_ + σ_*x*_ is roughly doubled for all time steps when photons are not allowed to have negative momentum. The situation becomes worse at later times due to the much weaker localization occurring in the model with no negative momentum modes.

We finish this work with an illustration of the main exciton transport phenomena exhibited by our model. The results presented here probe the transient dynamics of exciton transport, which differs from previous studies that focused on steady-state properties. Figure 8a,c shows representative trajectories for the exciton width under a wide range of disorder strengths with Ω_*R*_ = 0.05 and Ω_*R*_ = 0.1 eV, respectively. These trajectories show ballistic transport in the early subpicosecond window followed by a transient diffusive dynamics. For Ω_*R*_ = 0.1 eV (Figure 8c), wave packet localization takes place within the time range of 2–4 ps for σ_*M*_/Ω_*R*_ > 0.1. In contrast, when Ω_*R*_ = 0.05 eV and σ_*M*_/Ω_*R*_ < 0.8 (Figure 8a), localization happens at later times, between 4 and 5 ps. However, at larger values of σ_*M*_, localization occurs on a longer time scale *t* > 5 ps. This is likely caused by strong disorder effects, which are observed earlier for smaller values of Ω_*R*_. Indeed, we also observe clear signatures of recently reported disorder-enhanced transport (DET) phenomena.^41,43,61,62^ For example, Figure 8c shows that the wave packet localization length is an increasing function of σ_*M*_ for all values of σ_*M*_ ≥ 0.1 eV.

**Figure 8:**
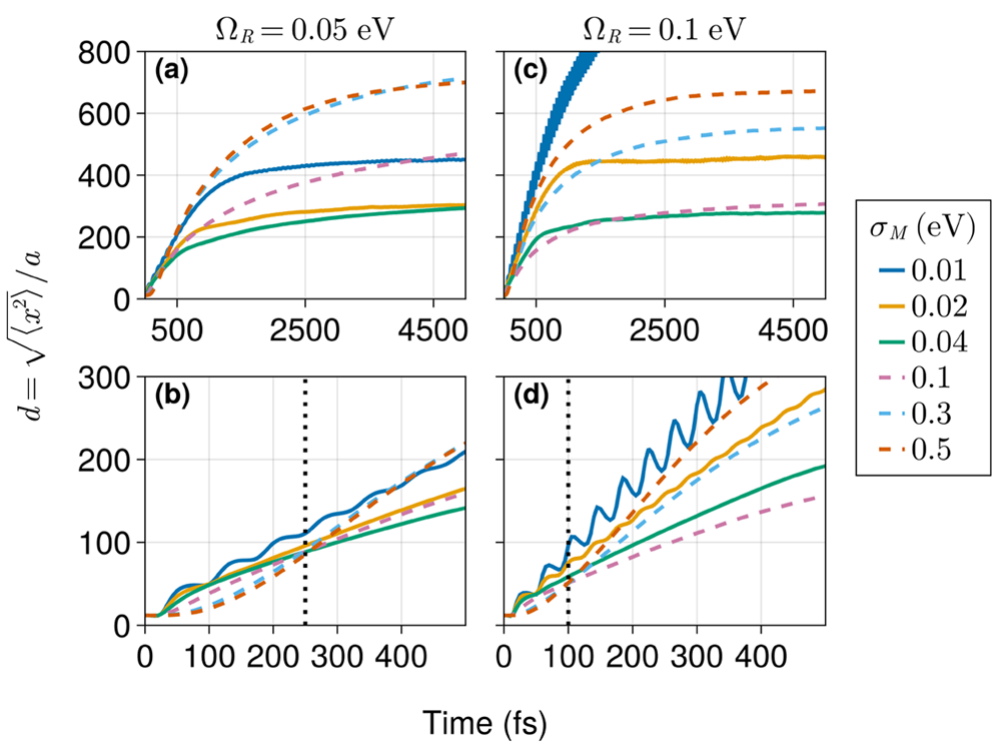
Wave packet spread (*d*) over time for several values of energetic disorder. The Rabi splitting is set to 0.05 eV (a and b) and 0.1 eV (c and d), and the cavity is in resonance with the average site excitation energy. Panels b and d present views into the subpicosecond window of panels c and d, respectively, with dotted vertical lines indicating the time scale for DET emergence. Dashed lines represent trajectories where DET is observed. System size is *N*_*M*_ = 5000 with *N*_*c*_ = 1001. Each trajectory represents an average of over 100 realizations.

Our wave packet simulations allow us to gain direct insight into the transient DET dynamics. We find that at very short times *t* < 100 fs (Figure 8b), with Ω_*R*_ = 0.05 eV, the exciton transport under strong disorder is essentially suppressed (highly subdiffusive). Figure 8b,d also marks the crossover of exciton width trajectories under strong over weak disorder and allows us to visualize the time scales required for DET manifestation. These results show that DET emerges earlier under stronger collective coupling, as demonstrated by the shift from 250 to 100 fs of the time required for DET to operate and the corresponding shortening of the initial subdiffusive dynamics as Ω_*R*_ increases from 0.05 to 0.1 eV.

Our findings show that the coherent evolution of exciton wave packets in photonic wires under strong light–matter coupling leads to DET operating on realistic time scales. However, the relevance of DET in the presence of dephasing induced by dynamical disorder (e.g., interaction with a thermal bath) remains an open question to be addressed in future work.

In summary, our work presents several key features of space-time-resolved exciton wave packet evolution in a lossless polaritonic wire. We have reported time-resolved exciton dynamics explicitly showing ballistic, diffusive, and subdiffusive polariton-assisted exciton transport in disordered wires under a variety of conditions of weak and strong energetic disorder. Our simulations enabled our unveiling of a short period of largely suppressed exciton propagation under strong disorder. This feature is Rabi splitting-dependent and arises prior to the onset of the disorder-enhanced transport regime in ultrafast time scales.

The convergence of our simulations was investigated thoroughly with respect to parameters such as the cavity length (number of sites) and number of photon modes. We found that a small number of sites can reproduce the very early dynamics of the system appropriately. Still, qualitatively incorrect results arise when the wave packet spreads over a length scale of the same order as the system size (the observed exciton wave packet dynamics was essentially independent of the density of photonic states). We have also demonstrated that a multimode description of the radiation field, covering a sufficiently large energy interval, is necessary to properly describe the system evolution, especially at early propagation times. This energy range can be estimated as 0.4 eV + 2Ω_*R*_. For example, when Ω_*R*_ = 0.1 eV and *E*_*M*_ = 2.0 eV, we find that a cutoff energy of 2.6 eV includes the most relevant cavity modes.

In the presence of static disorder, the photonic distributions become more intricate but the convergence trends observed in the absence of disorder are maintained. Hence, the simpler zero-disorder simulations requiring no ensemble averaging may provide a reference for constructing an optimal set of photon modes. Investigating the weight of each EM mode to the overall dynamics, we found that *both* light–matter resonances and the mean initial exciton quasimomentum play a fundamental role in determining the dominant microcavity modes. However, upon a substantial increase in the Rabi splitting or static disorder, we observe a transition into a quasi-ergodic regime where the photon weight approaches a uniform distribution over a large interval of wave vectors. Overall, our findings highlight the rich diversity of exciton coherent transport phenomena in polaritonic wires and emphasize that a multimode description of the radiation field is essential to describing them accurately.

## Supporting Information Available

Additional plots for finite-size and EM truncation error using different parameters, total photon probabilities, and analytical expressions for photon weight distributions (PDF)
Transparent Peer Review report available (PDF)

## References

[ref1] Kavokin A. V.; Baumberg J. J.; Malpuech G.; Laussy F. P. Microcavities; Oxford University Press: London, 2017.

[ref2] Lidzey D. G.; Coles D. M. Organic and Hybrid Photonic Crystals; Springer International Publishing: Cham, 2015; pp 243–273.

[ref3] Ebbesen T. W. Hybrid Light–Matter States in a Molecular and Material Science Perspective. Acc. Chem. Res. 2016, 49, 2403–2412. 10.1021/acs.accounts.6b00295. 27779846

[ref4] Coles D. M.; Somaschi N.; Michetti P.; Clark C.; Lagoudakis P. G.; Savvidis P. G.; Lidzey D. G. Polariton-mediated energy transfer between organic dyes in a strongly coupled optical microcavity. Nat. Mater. 2014, 13, 712–719. 10.1038/nmat3950. 24793357

[ref5] Zhong X.; Chervy T.; Zhang L.; Thomas A.; George J.; Genet C.; Hutchison J. A.; Ebbesen T. W. Energy Transfer between Spatially Separated Entangled Molecules. Angew. Chem. 2017, 129, 9162–9166. 10.1002/ange.201703539. PMC5575472 28598527

[ref6] Xiang B.; Ribeiro R. F.; Du M.; Chen L.; Yang Z.; Wang J.; Yuen-Zhou J.; Xiong W. Intermolecular vibrational energy transfer enabled by microcavity strong light–matter coupling. Science 2020, 368, 665–667. 10.1126/science.aba3544. 32381725

[ref7] Hagenmüller D.; Schachenmayer J.; Schütz S.; Genes C.; Pupillo G. Cavity-Enhanced Transport of Charge. Phys. Rev. Lett. 2017, 119, 223601 10.1103/PhysRevLett.119.223601. 29286774

[ref8] Hagenmüller D.; Schütz S.; Schachenmayer J.; Genes C.; Pupillo G. Cavity-assisted mesoscopic transport of fermions: Coherent and dissipative dynamics. Phys. Rev. B 2018, 97, 205303 10.1103/PhysRevB.97.205303.

[ref9] Garcia-Vidal F. J.; Ciuti C.; Ebbesen T. W. Manipulating matter by strong coupling to vacuum fields. Science 2021, 373, eabd0336 10.1126/science.abd0336. 34244383

[ref10] Wang M.; Hertzog M.; Börjesson K. Polariton-assisted excitation energy channeling in organic heterojunctions. Nat. Commun. 2021, 12, 1874 10.1038/s41467-021-22183-3. 33767204 PMC7994571

[ref11] Guo Q.; Wu B.; Du R.; Ji J.; Wu K.; Li Y.; Shi Z.; Zhang S.; Xu H. Boosting Exciton Transport in WSe2 by Engineering Its Photonic Substrate. ACS Photonics 2022, 9, 2817–2824. 10.1021/acsphotonics.2c00652.

[ref12] Orgiu E.; George J.; Hutchison J. A.; Devaux E.; Dayen J. F.; Doudin B.; Stellacci F.; Genet C.; Schachenmayer J.; Genes C.; et al. Conductivity in organic semiconductors hybridized with the vacuum field. Nat. Mater. 2015, 14, 1123–1129. 10.1038/nmat4392. 26366850

[ref13] Krainova N.; Grede A. J.; Tsokkou D.; Banerji N.; Giebink N. C. Polaron Photoconductivity in the Weak and Strong Light-Matter Coupling Regime. Phys. Rev. Lett. 2020, 124, 177401 10.1103/PhysRevLett.124.177401. 32412265

[ref14] Nagarajan K.; George J.; Thomas A.; Devaux E.; Chervy T.; Azzini S.; Joseph K.; Jouaiti A.; Hosseini M. W.; Kumar A.; et al. Conductivity and Photoconductivity of a p-Type Organic Semiconductor under Ultrastrong Coupling. ACS Nano 2020, 14, 10219–10225. 10.1021/acsnano.0c03496. 32806034

[ref15] Bhatt P.; Kaur K.; George J. Enhanced Charge Transport in Two-Dimensional Materials through Light–Matter Strong Coupling. ACS Nano 2021, 15, 13616–13622. 10.1021/acsnano.1c04544. 34347448

[ref16] Liu B.; Huang X.; Hou S.; Fan D.; Forrest S. R. Photocurrent generation following long-range propagation of organic exciton–polaritons. Optica 2022, 9, 1029–1036. 10.1364/OPTICA.461025.

[ref17] Dunkelberger A. D.; Simpkins B. S.; Vurgaftman I.; Owrutsky J. C. Vibration-Cavity Polariton Chemistry and Dynamics. Annu. Rev. Phys. Chem. 2022, 73, 429–451. 10.1146/annurev-physchem-082620-014627. 35081324

[ref18] Hirai K.; Hutchison J. A.; Uji-i H. Recent Progress in Vibropolaritonic Chemistry. ChemPlusChem. 2020, 85, 1981–1988. 10.1002/cplu.202000411. 32869494

[ref19] Li T. E.; Cui B.; Subotnik J. E.; Nitzan A. Molecular Polaritonics: Chemical Dynamics Under Strong Light–Matter Coupling. Annu. Rev. Phys. Chem. 2022, 73, 43–71. 10.1146/annurev-physchem-090519-042621. 34871038

[ref20] Galego J.; Garcia-Vidal F. J.; Feist J. Suppressing photochemical reactions with quantized light fields. Nat. Commun. 2016, 7, 13841 10.1038/ncomms13841. 27941754 PMC5159835

[ref21] Imamoglu A.; Ram R. J.; Pau S.; Yamamoto Y. Nonequilibrium condensates and lasers without inversion: Exciton-polariton lasers. Phys. Rev. A 1996, 53, 4250–4253. 10.1103/PhysRevA.53.4250. 9913395

[ref22] Sanvitto D.; Kéna-Cohen S. The road towards polaritonic devices. Nat. Mater. 2016, 15, 1061–1073. 10.1038/nmat4668. 27429208

[ref23] Zhong X.; Chervy T.; Wang S.; George J.; Thomas A.; Hutchison J. A.; Devaux E.; Genet C.; Ebbesen T. W. Non-Radiative Energy Transfer Mediated by Hybrid Light-Matter States. Angew. Chem., Int. Ed. 2016, 55, 6202–6206. 10.1002/anie.201600428. 27072296

[ref24] Myers D. M.; Mukherjee S.; Beaumariage J.; Snoke D. W.; Steger M.; Pfeiffer L. N.; West K. Polariton-enhanced exciton transport. Phys. Rev. B 2018, 98, 235302 10.1103/PhysRevB.98.235302.

[ref25] Lerario G.; Ballarini D.; Fieramosca A.; Cannavale A.; Genco A.; Mangione F.; Gambino S.; Dominici L.; De Giorgi M.; Gigli G.; et al. High-speed flow of interacting organic polaritons. Light: Sci. Appl. 2017, 6, e16212 10.1038/lsa.2016.212. 30167229 PMC6062184

[ref26] Hou S.; Khatoniar M.; Ding K.; Qu Y.; Napolov A.; Menon V. M.; Forrest S. R. Ultralong-Range Energy Transport in a Disordered Organic Semiconductor at Room Temperature Via Coherent Exciton-Polariton Propagation. Adv. Mater. 2020, 32, 2002127 10.1002/adma.202002127. 32484288

[ref27] Rozenman G. G.; Akulov K.; Golombek A.; Schwartz T. Long-Range Transport of Organic Exciton-Polaritons Revealed by Ultrafast Microscopy. ACS Photonics 2018, 5, 105–110. 10.1021/acsphotonics.7b01332.

[ref28] Pandya R.; Ashoka A.; Georgiou K.; Sung J.; Jayaprakash R.; Renken S.; Gai L.; Shen Z.; Rao A.; Musser A. J. Tuning the Coherent Propagation of Organic Exciton-Polaritons through Dark State Delocalization. Adv. Sci. 2022, 9, 2105569 10.1002/advs.202105569. PMC9218652 35474309

[ref29] Tichauer R. H.; Sokolovskii I.; Groenhof G. Tuning the Coherent Propagation of Organic Exciton-Polaritons through the Cavity Q-factor. arXiv 2023, 2304.13123 10.48550/arXiv.2304.13123. PMC10667804 37818758

[ref30] Balasubrahmaniyam M.; Simkhovich A.; Golombek A.; Sandik G.; Ankonina G.; Schwartz T. From enhanced diffusion to ultrafast ballistic motion of hybrid light–matter excitations. Nat. Mater. 2023, 22, 338–344. 10.1038/s41563-022-01463-3. 36646793

[ref31] Xu D.; Mandal A.; Baxter J. M.; Cheng S.-W.; Lee I.; Su H.; Liu S.; Reichman D. R.; Delor M. Ultrafast imaging of coherent polariton propagation and interactions. arXiv 2022, 2205.01176 10.48550/arXiv.2205.01176. PMC10313693 37391396

[ref32] Feist J.; Garcia-Vidal F. J. Extraordinary Exciton Conductance Induced by Strong Coupling. Phys. Rev. Lett. 2015, 114, 196402 10.1103/PhysRevLett.114.196402. 26024185

[ref33] Schachenmayer J.; Genes C.; Tignone E.; Pupillo G. Cavity-Enhanced Transport of Excitons. Phys. Rev. Lett. 2015, 114, 196403 10.1103/PhysRevLett.114.196403. 26024186

[ref34] Gonzalez-Ballestero C.; Feist J.; Gonzalo Badía E.; Moreno E.; Garcia-Vidal F. J. Uncoupled Dark States Can Inherit Polaritonic Properties. Phys. Rev. Lett. 2016, 117, 156402 10.1103/PhysRevLett.117.156402. 27768353

[ref35] Du M.; Martínez-Martínez L. A.; Ribeiro R. F.; Hu Z.; Menon V. M.; Yuen-Zhou J. Theory for polariton-assisted remote energy transfer. Chem. Sci. 2018, 9, 6659–6669. 10.1039/C8SC00171E. 30310599 PMC6115621

[ref36] Reitz M.; Mineo F.; Genes C. Energy transfer and correlations in cavity-embedded donor-acceptor configurations. Sci. Rep. 2018, 8, 9050 10.1038/s41598-018-27396-z. 29899401 PMC5998034

[ref37] Schäfer C.; Ruggenthaler M.; Appel H.; Rubio A. Modification of excitation and charge transfer in cavity quantum-electrodynamical chemistry. Proc. Natl. Acad. Sci. U.S.A. 2019, 116, 4883–4892. 10.1073/pnas.1814178116. 30733295 PMC6421448

[ref38] Scholes G. D. Polaritons and excitons: Hamiltonian design for enhanced coherence. Proc. R. Soc. A 2020, 476, 20200278 10.1098/rspa.2020.0278. 33223931 PMC7655764

[ref39] Li T. E.; Nitzan A.; Subotnik J. E. Collective vibrational strong coupling effects on molecular vibrational relaxation and energy transfer: Numerical insights via cavity molecular dynamics simulations. Angew. Chem. 2021, 133, 15661–15668. 10.1002/ange.202103920. 33957010

[ref40] Gettapola K.; Gunapala S. D.; Premaratne M. Directional energy transport in strongly coupled chiral quantum emitter plasmonic nanostructures. J. Phys.: Condens. Matter 2021, 33, 475301 10.1088/1361-648X/ac203f. 34425568

[ref41] Engelhardt G.; Cao J. Polarition localization and spectroscopic properties of disordered quantum emitters in spatially-extended microcavities. arXiv 2022, 2209.00290 10.48550/arXiv.2209.00290.

[ref42] Ribeiro R. F.; Suyabatmaz E. Vibrational polariton transport in disordered media. arXiv 2023, 2303.00547 10.48550/arXiv.2303.00547. 37458348

[ref43] Allard T. F.; Weick G. Disorder-enhanced transport in a chain of lossy dipoles strongly coupled to cavity photons. Phys. Rev. B 2022, 106, 245424 10.1103/PhysRevB.106.245424.

[ref44] Botzung T.; Hagenmüller D.; Schütz S.; Dubail J.; Pupillo G.; Schachenmayer J. Dark state semilocalization of quantum emitters in a cavity. Phys. Rev. B 2020, 102, 144202 10.1103/PhysRevB.102.144202.

[ref45] Agranovich V.; Gartstein Y. N. Nature and dynamics of low-energy exciton polaritons in semiconductor microcavities. Phys. Rev. B 2007, 75, 075302 10.1103/PhysRevB.75.075302.

[ref46] Tichauer R. H.; Feist J.; Groenhof G. Multi-scale dynamics simulations of molecular polaritons: The effect of multiple cavity modes on polariton relaxation. J. Chem. Phys. 2021, 154, 104112 10.1063/5.0037868. 33722041

[ref47] Ribeiro R. F. Multimode polariton effects on molecular energy transport and spectral fluctuations. Commun. Chem. 2022, 5, 48 10.1038/s42004-022-00660-0. 36697846 PMC9814737

[ref48] PolaritonicSystems.jl: Toolbox for representing and computing observables of polaritonic systems. https://github.com/RibeiroGroup/PolaritonicSystems.jl (accessed 2023-04-14).

[ref49] Besançon M.; Papamarkou T.; Anthoff D.; Arslan A.; Byrne S.; Lin D.; Pearson J. Distributions.jl: Definition and Modeling of Probability Distributions in the JuliaStats Ecosystem. J. Stat. Softw. 2021, 98, 1–30. 10.18637/jss.v098.i16.

[ref50] Danisch S.; Krumbiegel J. Makie.jl: Flexible high-performance data visualization for Julia. J. Open Source Softw. 2021, 6, 3349 10.21105/joss.03349.

[ref51] Agranovich V. M.; Litinskaia M.; Lidzey D. G. Cavity polaritons in microcavities containing disordered organic semiconductors. Phys. Rev. B 2003, 67, 085311 10.1103/PhysRevB.67.085311.

[ref52] Anderson P. W. Absence of diffusion in certain random lattices. Phys. Rev. 1958, 109, 1492 10.1103/PhysRev.109.1492.

[ref53] Allen P. B.; Kelner J. Evolution of a vibrational wave packet on a disordered chain. Am. J. Phys. 1998, 66, 497–506. 10.1119/1.18890.

[ref54] Mandal A.; Xu D.; Mahajan A.; Lee J.; Delor M.; Reichman D. R. Microscopic Theory of Multimode Polariton Dispersion in Multilayered Materials. Nano Lett. 2023, 23, 4082–4089. 10.1021/acs.nanolett.3c01017. 37103998

[ref55] Hoffmann N. M.; Lacombe L.; Rubio A.; Maitra N. T. Effect of many modes on self-polarization and photochemical suppression in cavities. J. Chem. Phys. 2020, 153, 104103 10.1063/5.0012723. 32933282

[ref56] Rokaj V.; Welakuh D. M.; Ruggenthaler M.; Rubio A. Light–matter interaction in the long-wavelength limit: no ground-state without dipole self-energy. Journal of Physics B: Atomic, Molecular and Optical Physics 2018, 51, 034005 10.1088/1361-6455/aa9c99.

[ref57] Litinskaya M.; Reineker P. Loss of coherence of exciton polaritons in inhomogeneous organic microcavities. Phys. Rev. B 2006, 74, 165320 10.1103/PhysRevB.74.165320.

[ref58] Litinskaya M. Propagation and localization of polaritons in disordered organic microcavities. Phys. Lett. A 2008, 372, 3898–3903. 10.1016/j.physleta.2008.02.062.

[ref59] Giordano M. Uncertainty propagation with functionally correlated quantities. arXiv 2016, 1610.00871 10.48550/arXiv.1610.00871.

[ref60] Akkermans E.; Montambaux G. Mesoscopic physics of electrons and photons; Cambridge University Press: Cambridge, 2007.

[ref61] Chávez N. C.; Mattiotti F.; Méndez-Bermúdez J.; Borgonovi F.; Celardo G. L. Disorder-Enhanced and Disorder-Independent Transport with Long-Range Hopping: Application to Molecular Chains in Optical Cavities. Phys. Rev. Lett. 2021, 126, 153201 10.1103/PhysRevLett.126.153201. 33929231

[ref62] Dubail J.; Botzung T.; Schachenmayer J.; Pupillo G.; Hagenmüller D. Large random arrowhead matrices: Multifractality, semilocalization, and protected transport in disordered quantum spins coupled to a cavity. Phys. Rev. A 2022, 105, 023714 10.1103/PhysRevA.105.023714.

