# Supplementary material for: Theoretical Analysis of Exciton Wave Packet Dynamics in Polaritonic Wires
Source: J Phys Chem Lett. 2023 Jun 14;14(24):5681–91. doi: 10.1021/acs.jpclett.3c01082 (PMC10291640; doi:10.1021/acs.jpclett.3c01082)
Supplement: Supplementary file 1 — jz3c01082_si_001.pdf [file jz3c01082_si_001.pdf]

Supporting Information

Theoretical Analysis of Exciton Wave Packet Dynamics in Polaritonic Wires

Gustavo J. R. Aroeira, Kyle T. Kairys, and Raphael F. Ribeiro*

Department of Chemistry and Cherry Emerson Center for Scientific Computation, Emory University, Atlanta, Georgia 30322, United States of America

E-mail: raphael.ribeiro@emory.edu

Table S1: List of symbols and notation used in this work

Symbol	Description	Value
N_M	Number of molecules in the wire.	Variable
N_c	Number of cavity modes used to describe the radiation field inside the cavity.	Variable
Ω_{R}	Rabi splitting: a measure of the collective light-matter interaction strength.	Variable
a	Intermolecular distance (or average value, if static disorder is included).	Variable
σ_{a}	Standard deviation of molecular positions.	Variable
E_{M}	Molecular excitation energy (or average value, if static disorder is included).	Variable
σ_{M}	Standard deviation of the distribution of molecular excitation energies.	Variable
L_z	Wire length along z dimension.	400 nm
L_y	Wire length along y dimension.	200 nm
L_x	Wire length along x dimension.	$N_M a$
ϵ	Relative static permittivity.	3
n_y	Cavity quantum number associated with the y dimension.	1
n_z	Cavity quantum number associated with the z dimension.	1
m_x	Cavity quantum number associated with the x dimension.	$\in \mathbb{Z}$
q	x-component of the wavevector $\mathbf{k}$	$2\pi m_x/L_x$.
σ_x	Initial wave packet width.	Variable
$\bar{q}_0$	Average initial exciton momentum along x	Variable

Contents

1. Finite-size Effects **S6**

2. Time dependence of the EM truncation error without disorder **S11**

3. Ω_R dependence of the EM truncation error without disorder **S14**

4. EM truncation error in the presence of disorder **S16**

5. Time-dependent photon probabilities **S19**

6. Average photon probabilities **S21**

7. Detuning effect on photon weight distribution without disorder **S22**

8. Photon weight distribution in the presence of disorder **S25**

9. Analytical Results for Photon weight distribution in the absence of disorder **S31**

List of Figures

S1 Wave packet width (d) vs. time for short (**upper panel**) and long (**lower panel**) propagation times for various system sizes (N_{M}). **Parameters:** $N_c = 1601$, $\Omega_R = 0.1$ eV, $a = 20$ nm, $E_M = 2.0$ eV, $\sigma_x = 60$ nm. No disorder. S6

S2 Wave packet width (d) vs. time for short (**upper panel**) and long (**lower panel**) propagation times for various system sizes (N_{M}). **Parameters:** $N_c = 1601$, $\Omega_R = 0.2$ eV, $a = 10$ nm, $E_M = 2.0$ eV, $\sigma_x = 60$ nm. No disorder. S7

S3 Wave packet width (d) over time for short (**upper panel**) and long (**lower panel**) propagation times for various system sizes (N_{M}). **Parameters:** $N_c = 1$, $\Omega_R = 0.1$ eV, $a = 10$ nm, $E_M = 2.0$ eV, $\sigma_x = 60$ nm. No disorder. S8

S4 Wave packet width (d) vs time for short (**upper panel**) and long (**lower panel**) propagation times for various system sizes (N_{M}). **Parameters:** $N_c = 201$, $\Omega_R = 0.1$ eV, $a = 10$ nm, $E_M = 2.0$, $E_M = 2.0$ eV, $\sigma_x = 60$ nm. No disorder. S9

S5 Wave packet width (d) vs time for short (**upper panel**) and long (**lower panel**) propagation times for various system sizes (N_{M}). **Parameters:** $Nc = 801$, $\Omega_R = 0.1$ eV, $a = 10$ nm, $E_M = 2.0$ eV, $\sigma_x = 60$ nm. No disorder. S10

S6 Error due to cavity modes truncation (w.r.t to $N_c = 1601$) as a function of (a) N_c and (b) cavity cutoff energy. Error computed over 0.5 ps of simulation. The exponential profile shown is the same as in Fig. 3. **Parameters:** $N_M = 5000$, $\Omega_R = 0.1$ eV, $a = 10$ nm, $E_M = 2.0$ eV, $\sigma_x = 60$ nm. No disorder. S11

S7 Error due to cavity modes truncation (w.r.t to $N_c = 1601$) as a function of (a) N_c and (b) cavity cutoff energy. Error computed over 1 ps of simulation. The exponential profile shown is the same as in Fig. 3. **Parameters:** $N_M = 5000$, $\Omega_R = 0.1$ eV, $a = 10$ nm, $E_M = 2.0$ eV, $\sigma_x = 60$ nm. No disorder. S12

S8 Error due to cavity modes truncation (w.r.t to $N_c = 1601$) as a function of (a) N_c and (b) cavity cutoff energy. Error computed over 20 ps of simulation. The exponential profile shown is the same as in Fig. 3. **Parameters:** $N_M = 5000$, $\Omega_R = 0.1$ eV, $a = 10$ nm, $E_M = 2.0$ eV, $\sigma_x = 60$ nm. No disorder. S13

S9 Error due to cavity modes truncation (w.r.t to $N_c = 1601$) as a function of (a) N_c and (b) cavity cutoff energy. Error computed over 20 ps of simulation. The exponential profile shown is the same as in Fig. 3. **Parameters:** $N_M = 5000$, $\Omega_R = 0.05$ eV, $a = 10$ nm, $E_M = 2.0$ eV, $\sigma_x = 60$ nm. No disorder. S14

S10 Error due to cavity modes truncation (w.r.t to $N_c = 1601$) as a function of (a) N_c and (b) cavity cutoff energy. Error computed over 20 ps of simulation. The exponential profile shown is the same as in Fig. 3. **Parameters:** $N_M = 5000$, $\Omega_R = 0.3$ eV, $a = 10$ nm, $E_M = 2.0$ eV, $\sigma_x = 60$ nm. No disorder. S15

S11 Propagation under disorder for (a) $\sigma_M = 0.04$ eV and (b) $\sigma_M = 0.1$ eV for several values of N_c. The shaded region covers one standard deviation around the reference trajectory ($N_c = 1601$). (c) Error due to cavity modes truncation (w.r.t to Nc = 1601) as a function of the cutoff energy at various disorder strengths. Errors computed over 1 ps of simulation. **Parameters:** $N_M = 5000$, $\Omega_R = 0.2$ eV, $a = 10$ nm, $\omega_M = 2.0$ eV, $\sigma_x = 60$ nm. S16

S12 Propagation under disorder for (a) $\sigma_M = 0.02$ eV and (b) $\sigma_M = 0.05$ eV for several values of N_c. The shaded region covers one standard deviation around the reference trajectory ($N_c = 1601$). (c) Error due to cavity modes truncation (w.r.t to Nc = 1601) as a function of the cutoff energy at various disorder strengths. Errors computed over 1 ps of simulation. **Parameters:** $N_M = 5000$, $\Omega_R = 0.1$ eV, $a = 10$ nm, $\omega_M = 2.0$ eV, $\sigma_x = 180$ nm. S17

S13 Propagation under disorder for (a) $\sigma_M = 0.02$ eV and (b) $\sigma_M = 0.05$ eV for several values of N_c. The shaded region covers one standard deviation around the reference trajectory ($N_c = 1601$). (c) Error due to cavity modes truncation (w.r.t to Nc = 1601) as a function of the cutoff energy at various disorder strengths. Errors computed over 1 ps of simulation. **Parameters:** $N_M = 5000$, $\Omega_R = 0.1$ eV, $a = 10$ nm, $\omega_M = 2.2$ eV, $\sigma_x = 180$ nm. S18

S14 **Upper panel:** Total Exciton amplitude in the region of space with width covering 200 molecules centered at the point diametrically opposed to the wave packet center as a function of time. **Lower panel:** Total photon probability as a function of time. **Parameters:** $N_M = 1000$, $N_c = 1601$, $\Omega_R = 0.1$ eV, $a = 10$ nm, $E_M = 2.0$ eV, $\sigma_x = 60$ nm. No disorder. . S19

S15 Total photon probability over time for several disorder magnitudes and initial states. **Parameters:** $N_M = 5000$, $N_c = 401$ ($E_{\text{cutoff}} = 3.49$ eV), $\Omega_R = 0.1$ eV, $a = 10$ nm, $E_M = 2.2$ eV. S20

S16 Average photon probability over 5 ps for a wave packet with various initial configurations for different values of Rabi splitting and detuning. **Parameters:** $N_M = 5000$, $N_c = 401$ ($E_{\text{cutoff}} = 3.49$ eV), $a = 10$ nm. S21

S17 Cavity mode contribution under no disorder. The computation was performed over 5 ps using a 5 fs time step. Modes with $q > 0$ and $q \leq 0$ are represented by solid and dashed lines, respectively. **Parameters:** $N_M = 5000$, $N_c = 401$ ($E_{\text{cutoff}} = 3.49$ eV), $a = 10$ nm, $E_M = 1.8$ eV. S23

S18 Cavity mode contribution under no disorder. The computation was performed over 5 ps using a 5 fs time step. Modes with $q > 0$ and $q \leq 0$ are represented by solid and dashed lines, respectively. **Parameters:** $N_M = 5000$, $N_c = 401$ ($E_{\text{cutoff}} = 3.49$ eV), $a = 10$ nm, $E_M = 2.0$ eV. No disorder. S24

S19 Cavity mode contribution measured under energetic disorder of $\sigma_M = 0.005$ eV (a, b, c) and $\sigma_M = 0.1$ eV (d, e, f). The computation was performed over 5 ps using a 5 fs time step. Band plots cover one standard deviation around the average values of 100 realizations. **Parameters:** $N_M = 5000$, $N_c = 401$ ($E_{\text{cutoff}} = 3.49$ eV), $a = 10$ nm, $\sigma_a = 1$ nm, $E_M = 2.2$ eV, $\sigma_x = 120$ nm, $\bar{q}_0 \approx 0.00565$ nm^{-1}. S25

S20 Cavity mode contribution measured under energetic disorder of $\sigma_M = 0.02$ eV (a, b, c) and $\sigma_M = 0.05$ eV (d, e, f). The computation was performed over 500 fs using a 5 fs time step. Band plots cover one standard deviation around the average values of 100 realizations. **Parameters:** $N_M = 5000$, $N_c = 401$ ($E_{\text{cutoff}} = 3.49$ eV), $a = 10$ nm, $\sigma_a = 1$ nm, $E_M = 2.2$ eV, $\sigma_x = 120$ nm, $\bar{q}_0 \approx 0.00565$ nm^{-1}. S26

S21 Cavity mode contribution measured under energetic disorder of $\sigma_M = 0.02$ eV (a, b, c) and $\sigma_M = 0.05$ eV (d, e, f). The computation was performed over 5 ps using a 5 fs time step. Band plots cover one standard deviation around the average values of 100 realizations. **Parameters:** $N_M = 5000$, $N_c = 401$ ($E_{\text{cutoff}} = 3.49$ eV), $a = 10$ nm, $\sigma_a = 1$ nm, $E_M = 2.2$ eV, $\sigma_x = 120$ nm, $\bar{q}_0 = 0$. S27

S22 Cavity mode contribution measured under energetic disorder of $\sigma_M = 0.02$ eV (a, b, c) and $\sigma_M = 0.05$ eV (d, e, f). The computation was performed over 5 ps using a 5 fs time step. Band plots cover one standard deviation around the average values of 100 realizations. **Parameters:** $N_M = 5000$, $N_c = 401$ ($E_{\text{cutoff}} = 3.49$ eV), $a = 10$ nm, $\sigma_a = 1$ nm, $E_M = 2.2$ eV, $\sigma_x = 60$ nm, $\bar{q}_0 \approx 0.00565$ nm^{-1}. S28

S23 Cavity mode contribution measured under energetic disorder of $\sigma_M = 0.02$ eV (a, b, c) and $\sigma_M = 0.05$ eV (d, e, f). The computation was performed over 5 ps using a 5 fs time step. Band plots cover one standard deviation around the average values of 100 realizations. **Parameters:** $N_M = 5000$, $N_c = 401$ ($E_{\text{cutoff}} = 3.49$ eV), $a = 10$ nm, $\sigma_a = 1$ nm, $E_M = 2.2$ eV, $\sigma_x = 180$ nm, $\bar{q}_0 \approx 0.00565$ nm^{-1}. S29

S24 Cavity mode contribution measured under energetic disorder of $\sigma_M = 0.02$ eV (a, b, c) and $\sigma_M = 0.05$ eV (d, e, f). The computation was performed over 5 ps using a 5 fs time step. Band plots cover one standard deviation around the average values of 100 realizations. **Parameters:** $N_M = 5000$, $N_c = 401$ ($E_{\text{cutoff}} = 3.49$ eV), $a = 10$ nm, $\sigma_a = 1$ nm, $E_M = 2.0$ eV, $\sigma_x = 120$ nm, $\bar{q}_0 \approx 0.00565$ nm^{-1}. S30

1. Finite-size Effects

Fig. S1-S5 show variations of Fig.2(a) and (b) obtained with different parameters. In Fig. S1, the larger system size caused by setting $a = 20$ nm induces a substantial increase in the EM truncation error of the system with $N_M = 2 \cdot 10^4$. This occurs because the cutoff energy with such a large value of N_M is only 2.46 eV when $N_c = 1601$. Thus, the anomalous deviation observed for this system size is due to the EM field truncation, as discussed in the main text.

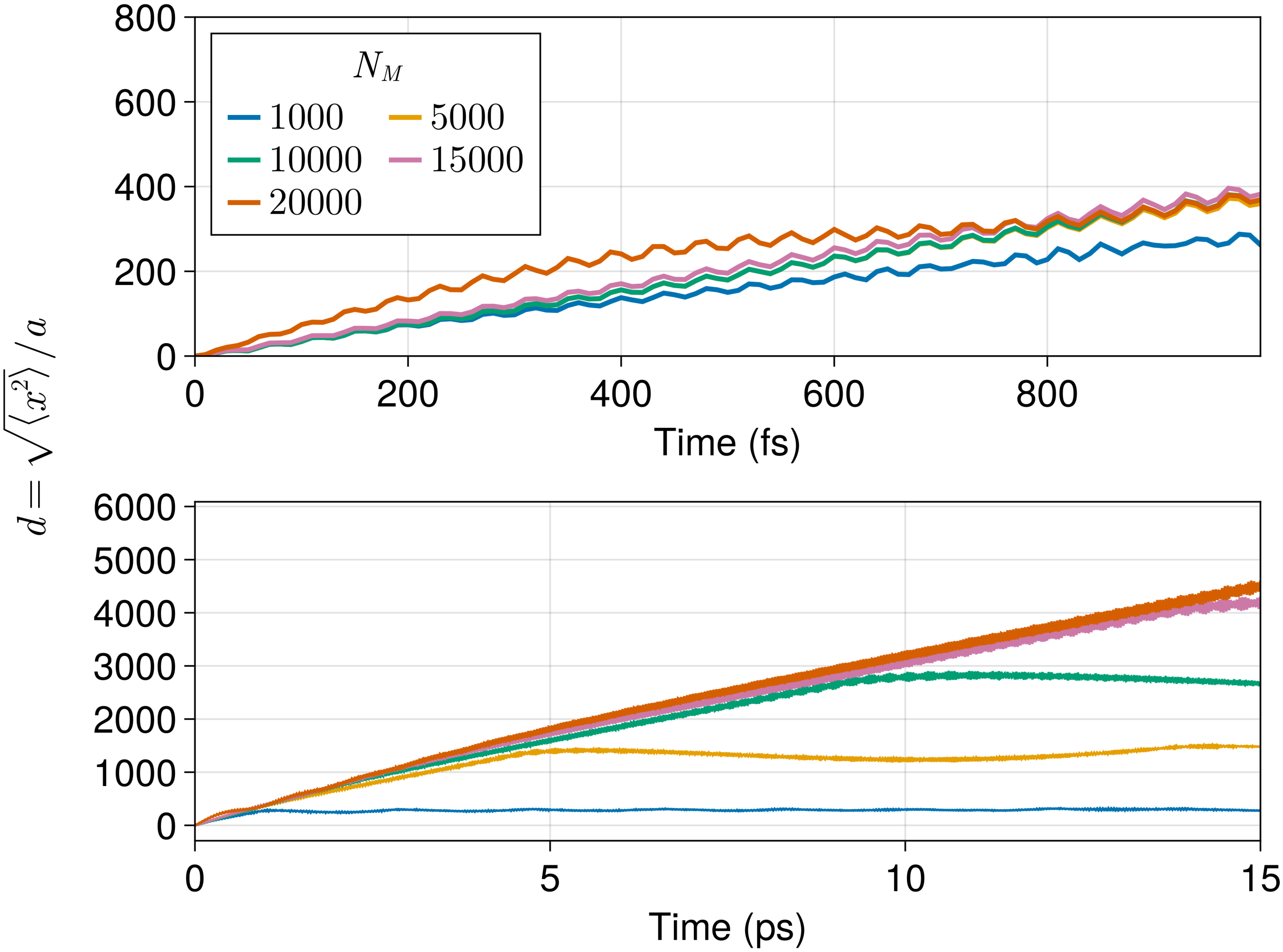

Figure S1: Wave packet width (d) vs. time for short (**upper panel**) and long (**lower panel**) propagation times for various system sizes (N_{M}). **Parameters:** $N_c = 1601$, $\Omega_R = 0.1$ eV, $a = 20$ nm, $E_M = 2.0$ eV, $\sigma_x = 60$ nm. No disorder.

Figs. S3-S5 show trajectories using different N_c values. It is clear that results are extremely affected by this parameter. Most notably, when $N_c = 1$, the wave packet width undergoes unreasonably large oscillations in striking contrast to the quasiballistic transport observed with sufficiently large N_c.

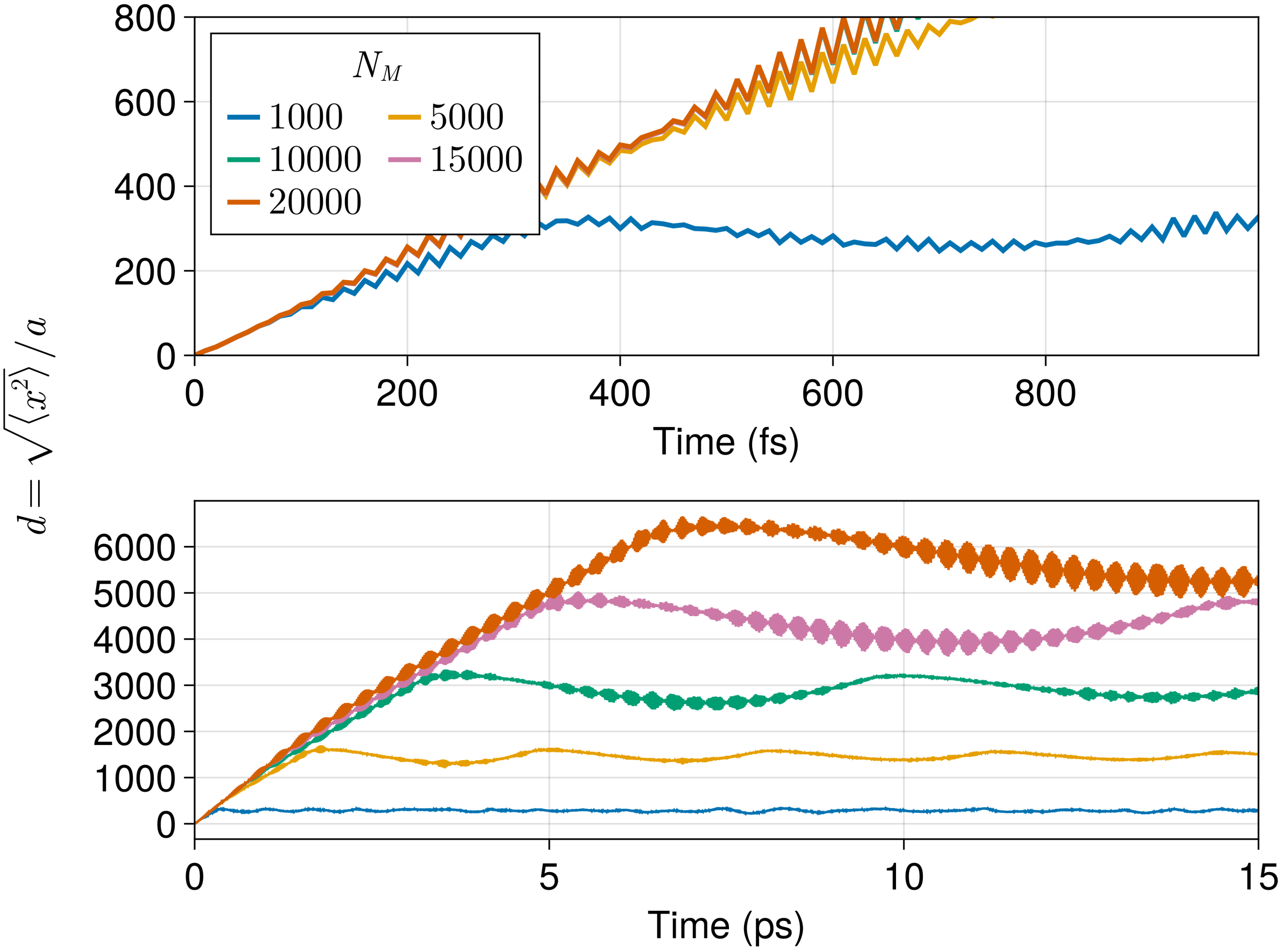

Figure S2: Wave packet width (d) vs. time for short (**upper panel**) and long (**lower panel**) propagation times for various system sizes (N_{M}). **Parameters:** $N_c = 1601$, $\Omega_R = 0.2$ eV, $a = 10$ nm, $E_M = 2.0$ eV, $\sigma_x = 60$ nm. No disorder.

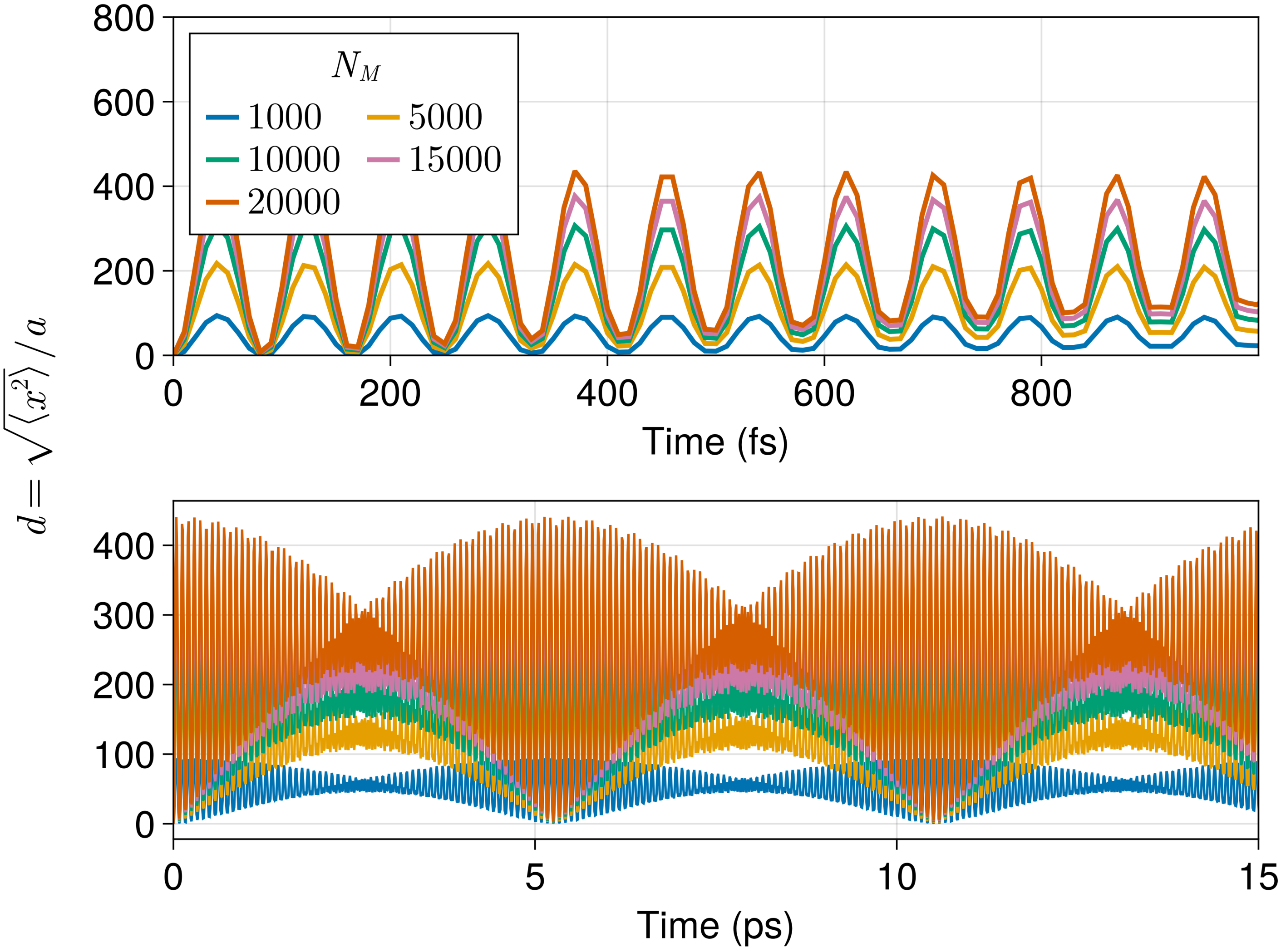

Figure S3: Wave packet width (d) over time for short (**upper panel**) and long (**lower panel**) propagation times for various system sizes (N_{M}). **Parameters:** $N_c = 1$, $\Omega_R = 0.1$ eV, $a = 10$ nm, $E_M = 2.0$ eV, $\sigma_x = 60$ nm. No disorder.

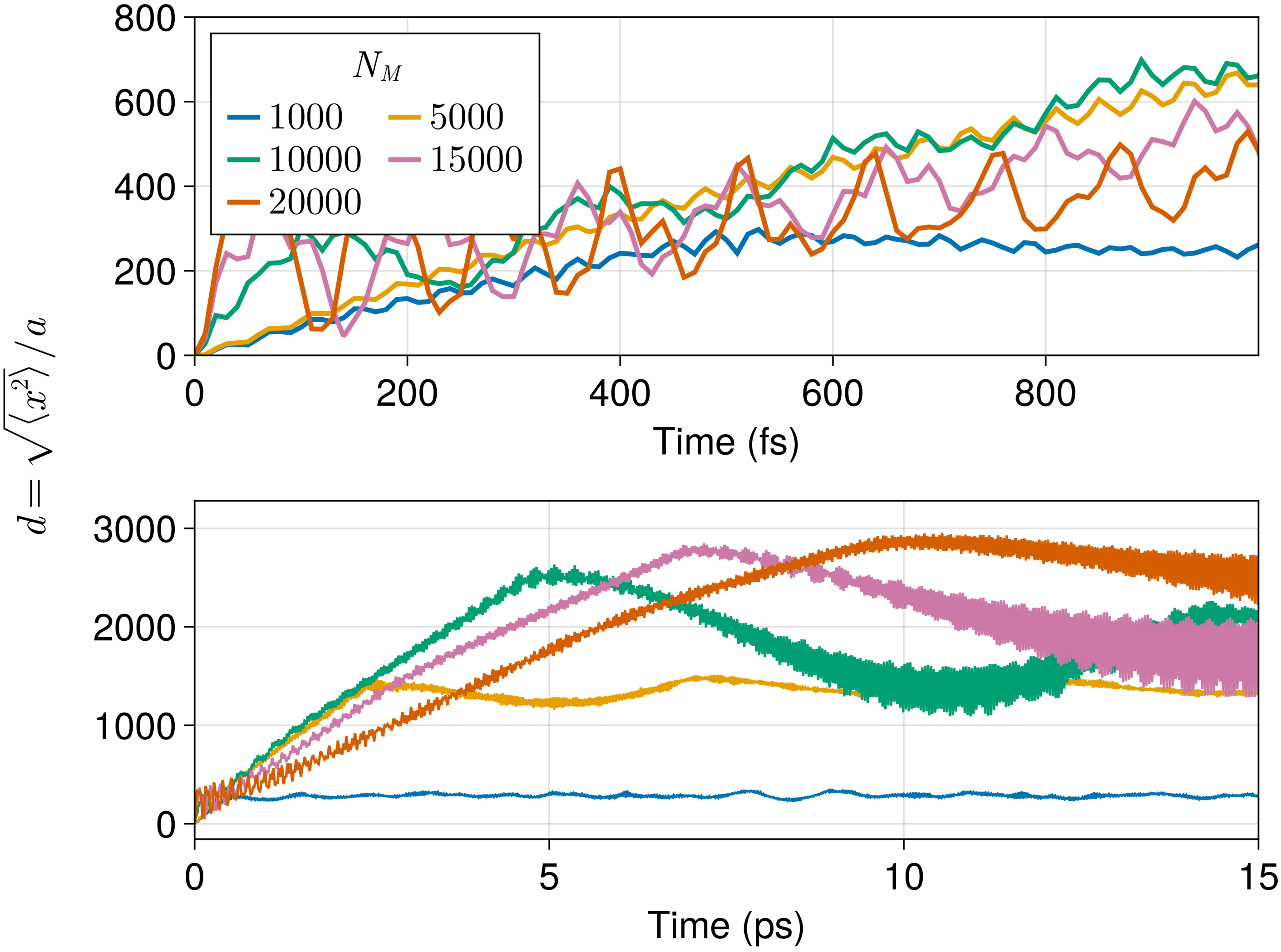

Figure S4: Wave packet width (d) vs time for short (**upper panel**) and long (**lower panel**) propagation times for various system sizes (N_{M}). **Parameters:** $N_c = 201$, $\Omega_R = 0.1$ eV, $a = 10$ nm, $E_M = 2.0$, $E_M = 2.0$ eV, $\sigma_x = 60$ nm. No disorder.

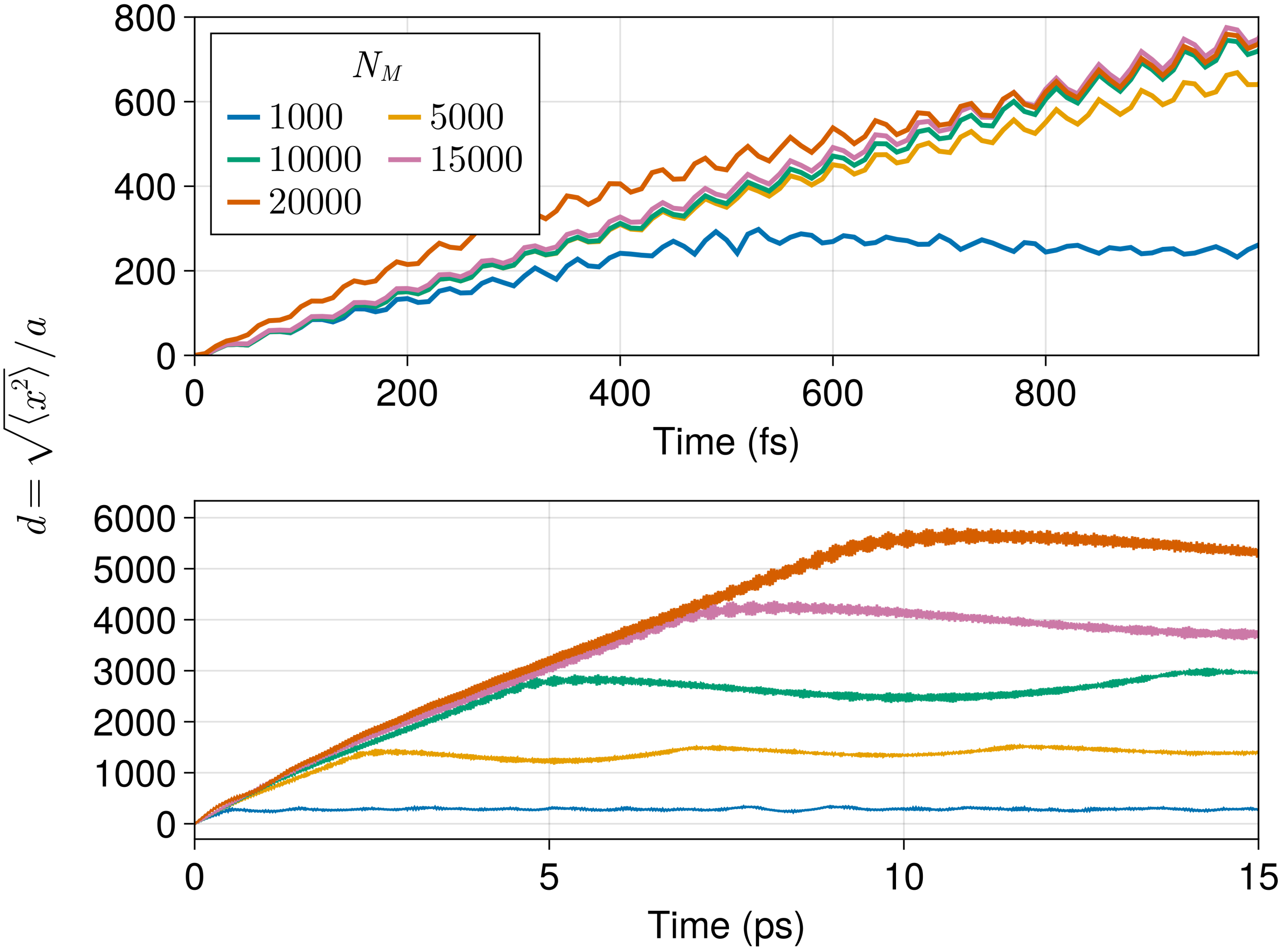

Figure S5: Wave packet width (d) vs time for short (**upper panel**) and long (**lower panel**) propagation times for various system sizes (N_{M}). **Parameters:** $Nc = 801$, $\Omega_R = 0.1$ eV, $a = 10$ nm, $E_M = 2.0$ eV, $\sigma_x = 60$ nm. No disorder.

2. Time dependence of the EM truncation error without disorder

Figs. S6−S8 show variations of Figs. 3(a) and 3(b) with different total simulation times over which the average error is computed. Note that, for the sake of comparison, the exponential profile $\exp[-\alpha(E_{\rm cutoff} - E_{\rm min})]$ is the same as in Fig. 3(b), (i.e. obtained from the error computed over 5 ps). If the simulation time is too small (0.5 ps), the computed error is significantly larger [Fig. S6], decreasing for 1 ps [Fig. S7]. Finally, Fig. S8 shows the exponential decay profile of the error computed in Fig. 3(b) (over 5 ps) agrees very well with the errors evaluated over 20 ps, indicating that 5 ps propagation produces converged errors.

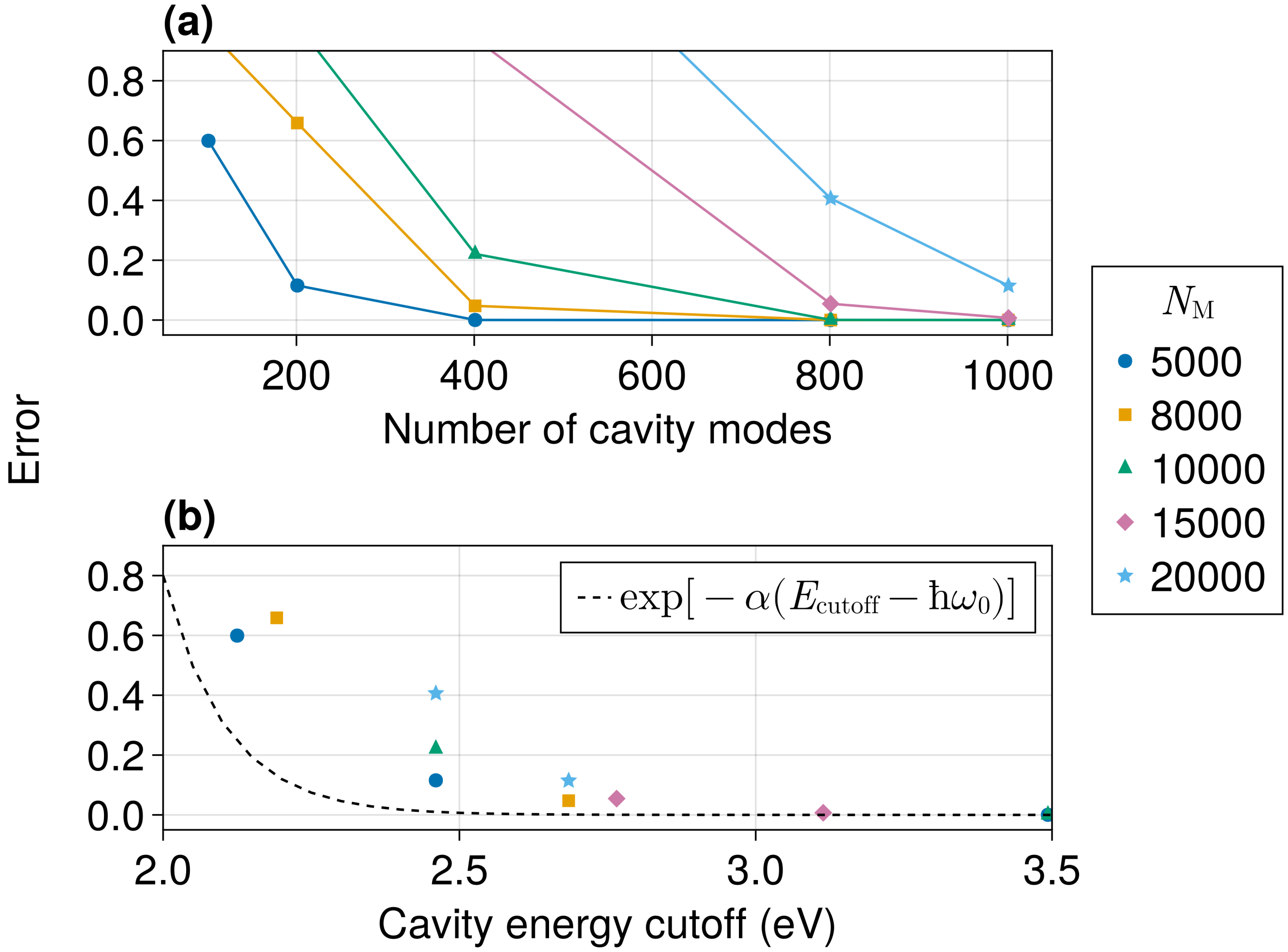

Figure S6: Error due to cavity modes truncation (w.r.t to $N_c = 1601$) as a function of (a) N_c and (b) cavity cutoff energy. Error computed over 0.5 ps of simulation. The exponential profile shown is the same as in Fig. 3. **Parameters:** $N_M = 5000$, $\Omega_R = 0.1$ eV, $a = 10$ nm, $E_M = 2.0$ eV, $\sigma_x = 60$ nm. No disorder.

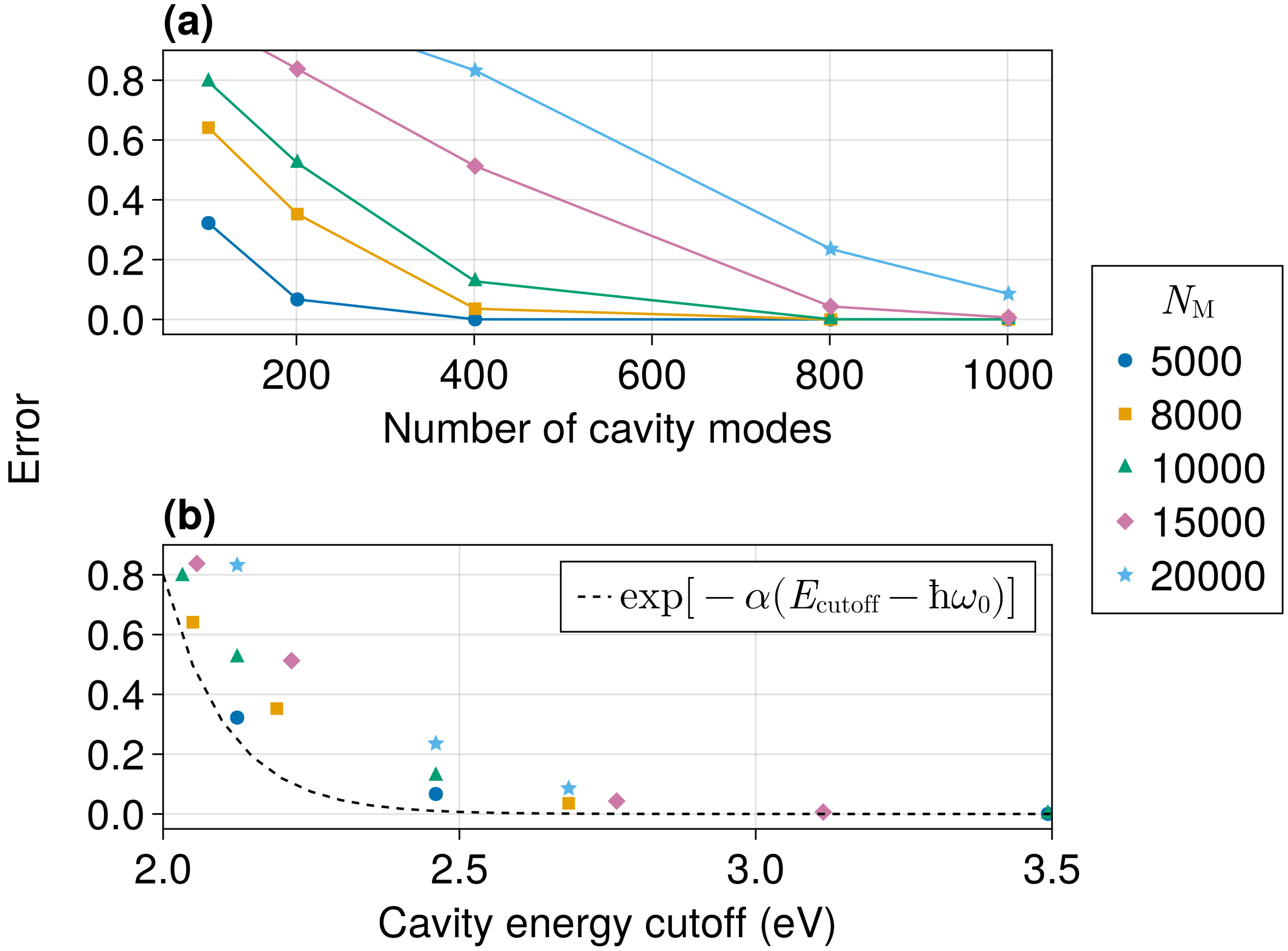

Figure S7: Error due to cavity modes truncation (w.r.t to $N_c = 1601$) as a function of (a) N_c and (b) cavity cutoff energy. Error computed over 1 ps of simulation. The exponential profile shown is the same as in Fig. 3. **Parameters:** $N_M = 5000$, $\Omega_R = 0.1$ eV, $a = 10$ nm, $E_M = 2.0$ eV, $\sigma_x = 60$ nm. No disorder.

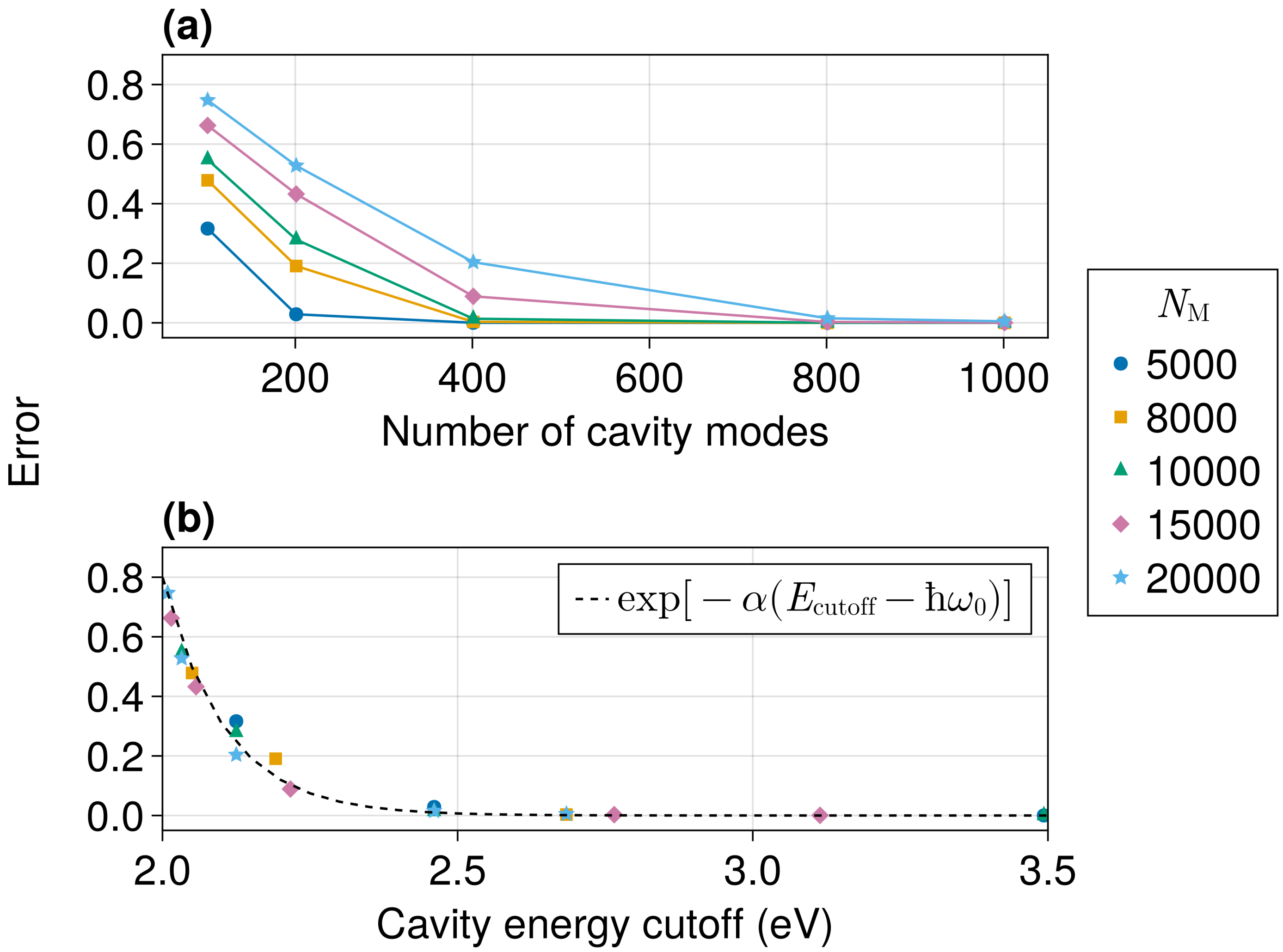

Figure S8: Error due to cavity modes truncation (w.r.t to $N_c = 1601$) as a function of (a) N_c and (b) cavity cutoff energy. Error computed over 20 ps of simulation. The exponential profile shown is the same as in Fig. 3. **Parameters:** $N_M = 5000$, $\Omega_R = 0.1$ eV, $a = 10$ nm, $E_M = 2.0$ eV, $\sigma_x = 60$ nm. No disorder.

3. Ω_R dependence of the EM truncation error without disorder

Figs. S9−S10 show variations of Figs. 3(a) and 3(b) using different values of Ω_R. Overall, there is a clear trend where, for larger Ω_R, the error convergence is slower. That is, larger Rabi splittings require larger EM energy cutoff values for the same accuracy. This justifies the trend observed in Fig. 3(c), where for a target error below 0.01, the EM energy cutoff grows as $2\Omega_R$.

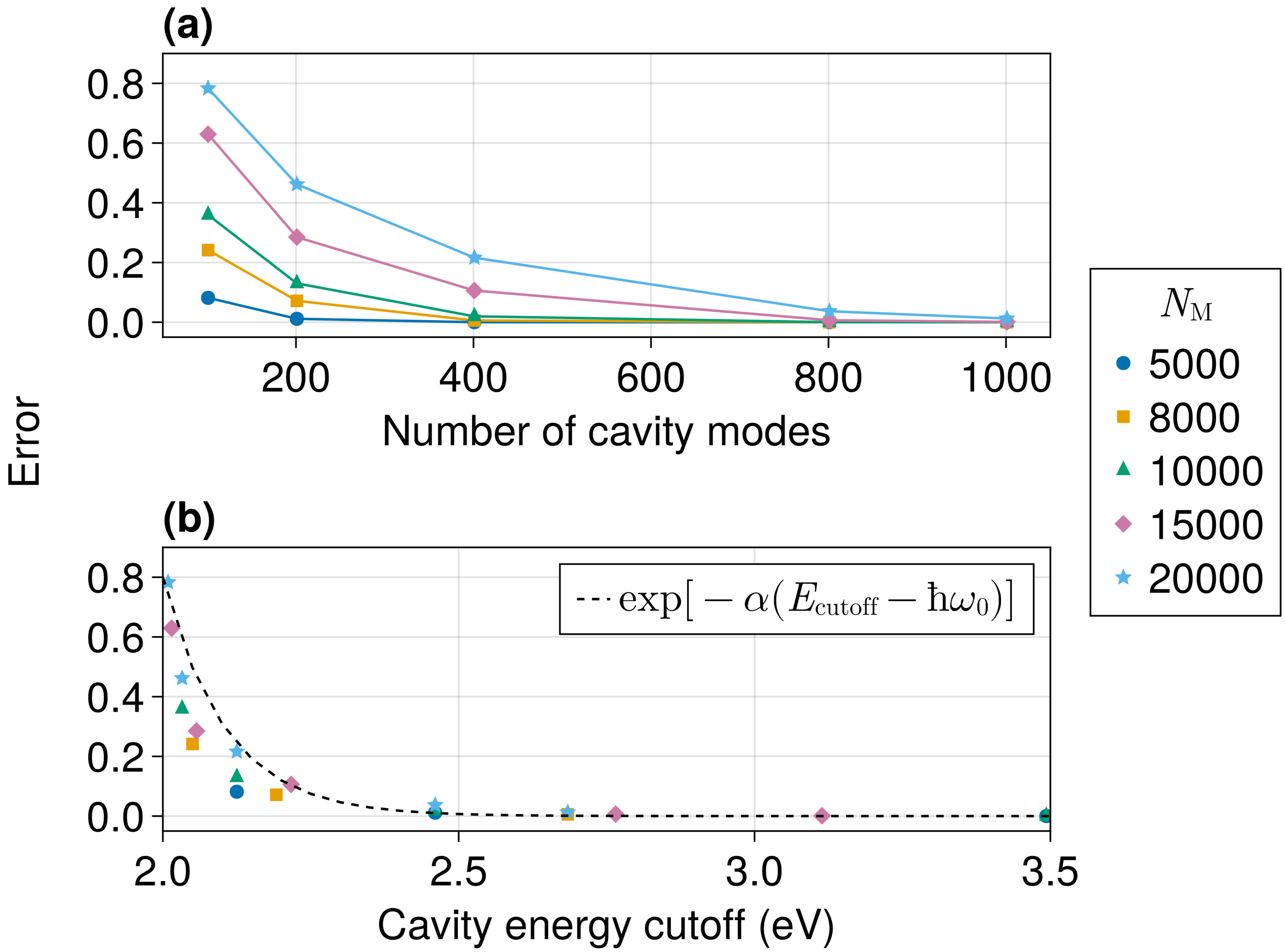

Figure S9: Error due to cavity modes truncation (w.r.t to $N_c = 1601$) as a function of (a) N_c and (b) cavity cutoff energy. Error computed over 20 ps of simulation. The exponential profile shown is the same as in Fig. 3. **Parameters:** $N_M = 5000$, $\Omega_R = 0.05$ eV, $a = 10$ nm, $E_M = 2.0$ eV, $\sigma_x = 60$ nm. No disorder.

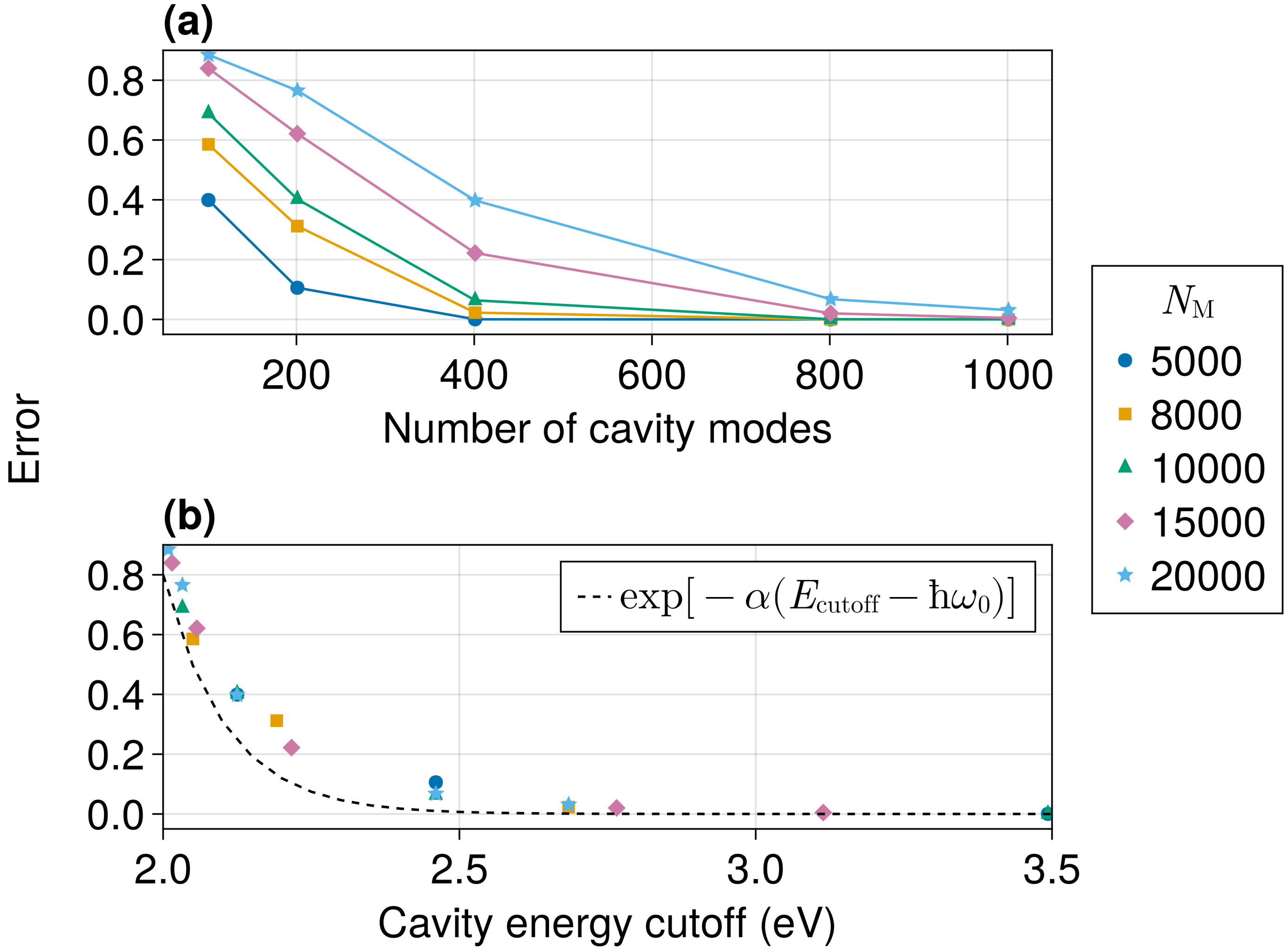

Figure S10: Error due to cavity modes truncation (w.r.t to $N_c = 1601$) as a function of (a) N_c and (b) cavity cutoff energy. Error computed over 20 ps of simulation. The exponential profile shown is the same as in Fig. 3. **Parameters:** $N_M = 5000$, $\Omega_R = 0.3$ eV, $a = 10$ nm, $E_M = 2.0$ eV, $\sigma_x = 60$ nm. No disorder.

4. EM truncation error in the presence of disorder

Figs. S11 – S13 show variations of Fig. 4 with different parameters. For the most part, these results largely agree with those presented in the main text and are presented here only for completeness.

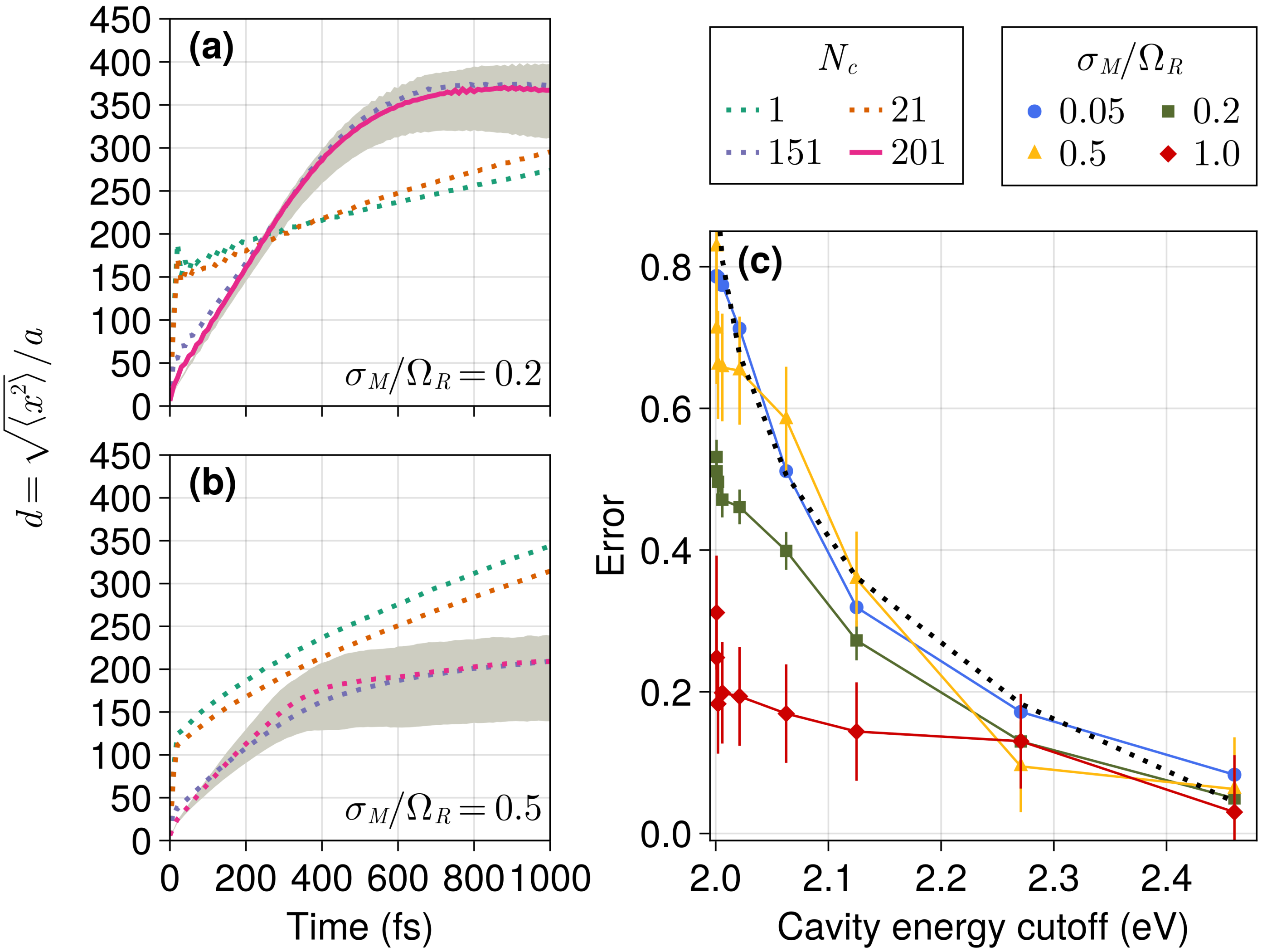

Figure S11: Propagation under disorder for (a) $\sigma_M = 0.04$ eV and (b) $\sigma_M = 0.1$ eV for several values of N_c. The shaded region covers one standard deviation around the reference trajectory ($N_c = 1601$). (c) Error due to cavity modes truncation (w.r.t to Nc = 1601) as a function of the cutoff energy at various disorder strengths. Errors computed over 1 ps of simulation. **Parameters:** $N_M = 5000$, $\Omega_R = 0.2$ eV, $a = 10$ nm, $\omega_M = 2.0$ eV, $\sigma_x = 60$ nm.

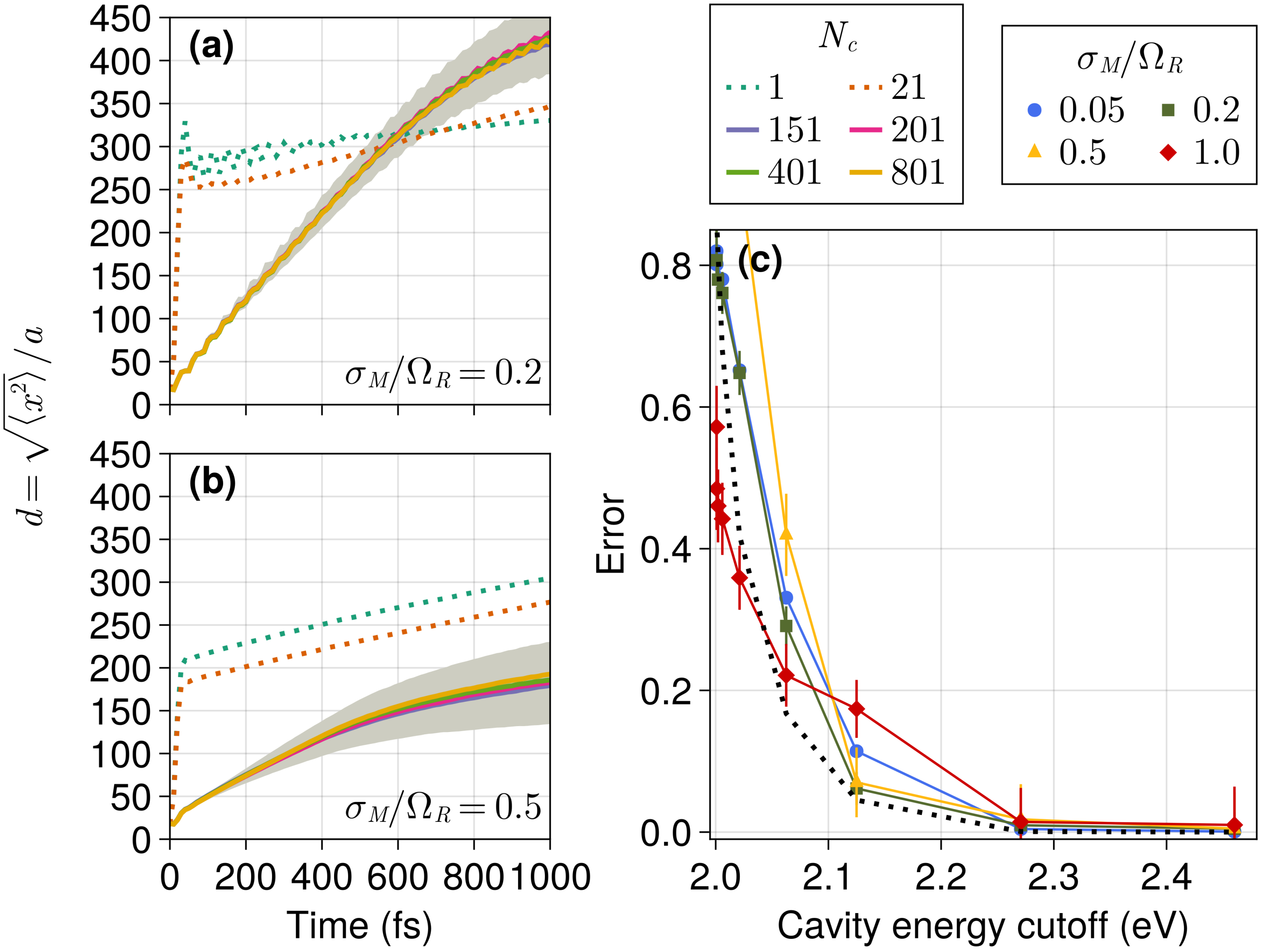

Figure S12: Propagation under disorder for (a) $\sigma_M = 0.02$ eV and (b) $\sigma_M = 0.05$ eV for several values of N_c. The shaded region covers one standard deviation around the reference trajectory ($N_c = 1601$). (c) Error due to cavity modes truncation (w.r.t to Nc = 1601) as a function of the cutoff energy at various disorder strengths. Errors computed over 1 ps of simulation. **Parameters:** $N_M = 5000$, $\Omega_R = 0.1$ eV, $a = 10$ nm, $\omega_M = 2.0$ eV, $\sigma_x = 180$ nm.

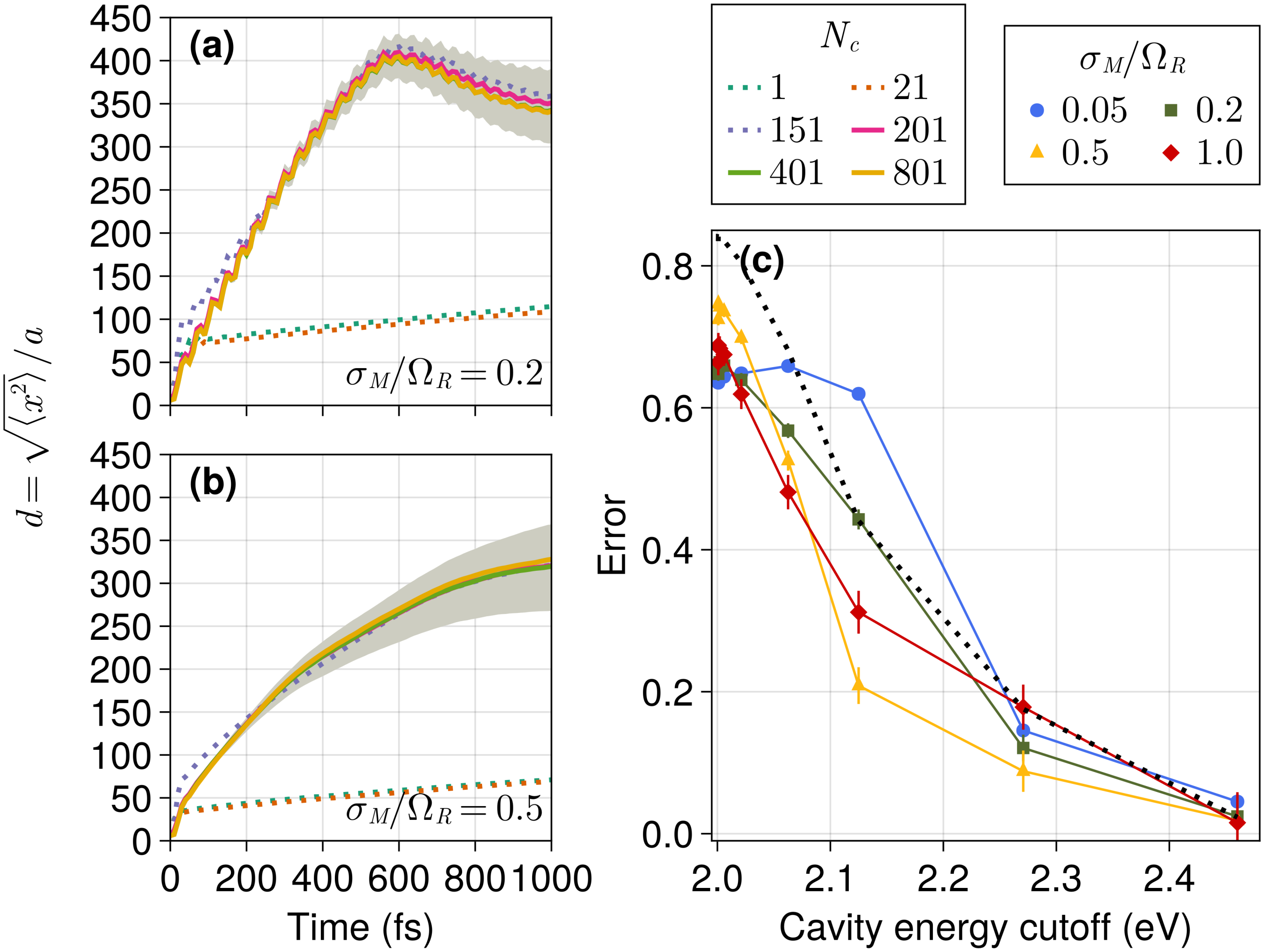

Figure S13: Propagation under disorder for (a) $\sigma_M = 0.02$ eV and (b) $\sigma_M = 0.05$ eV for several values of N_c. The shaded region covers one standard deviation around the reference trajectory ($N_c = 1601$). (c) Error due to cavity modes truncation (w.r.t to Nc = 1601) as a function of the cutoff energy at various disorder strengths. Errors computed over 1 ps of simulation. **Parameters:** $N_M = 5000$, $\Omega_R = 0.1$ eV, $a = 10$ nm, $\omega_M = 2.2$ eV, $\sigma_x = 180$ nm.

5. Time-dependent photon probabilities

Fig. S14 shows the total photon probability oscillates with approximately the same frequency as Rabi splitting. These same oscillations appear in Fig. 2 and are attributed to the continuous exchange of energy between light and matter (Rabi oscillations).

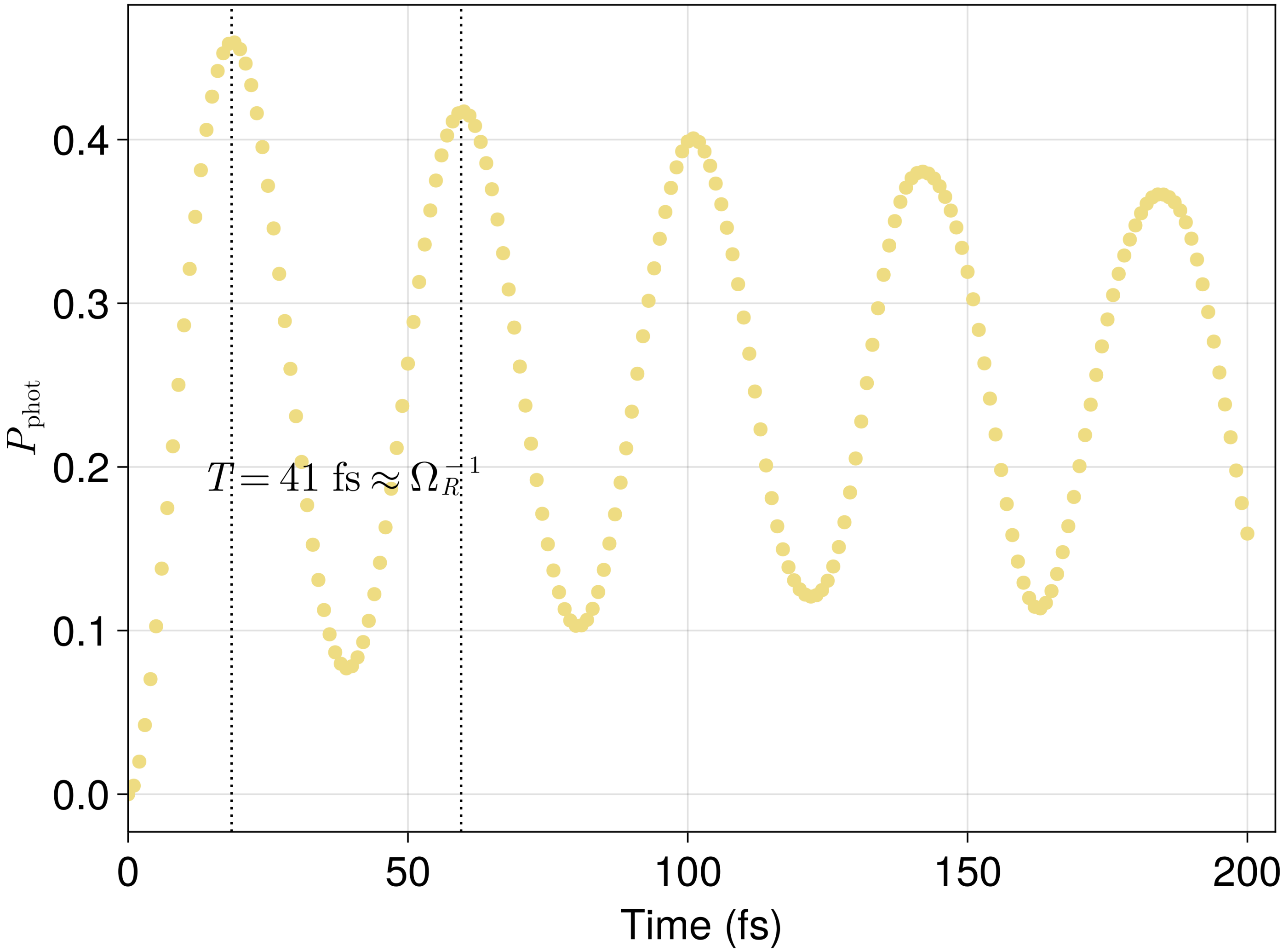

Figure S14: **Upper panel:** Total Exciton amplitude in the region of space with width covering 200 molecules centered at the point diametrically opposed to the wave packet center as a function of time. **Lower panel:** Total photon probability as a function of time. **Parameters:** $N_M = 1000$, $N_c = 1601$, $\Omega_R = 0.1$ eV, $a = 10$ nm, $E_M = 2.0$ eV, $\sigma_x = 60$ nm. No disorder.

Fig. S15 shows the time evolution of the total photon probability over 300 fs of simulation at variable disorder. The general pattern shows a rapid increase at the very early stages of the dynamics, followed by damped oscillations. The damping strength increases quickly with the static disorder.

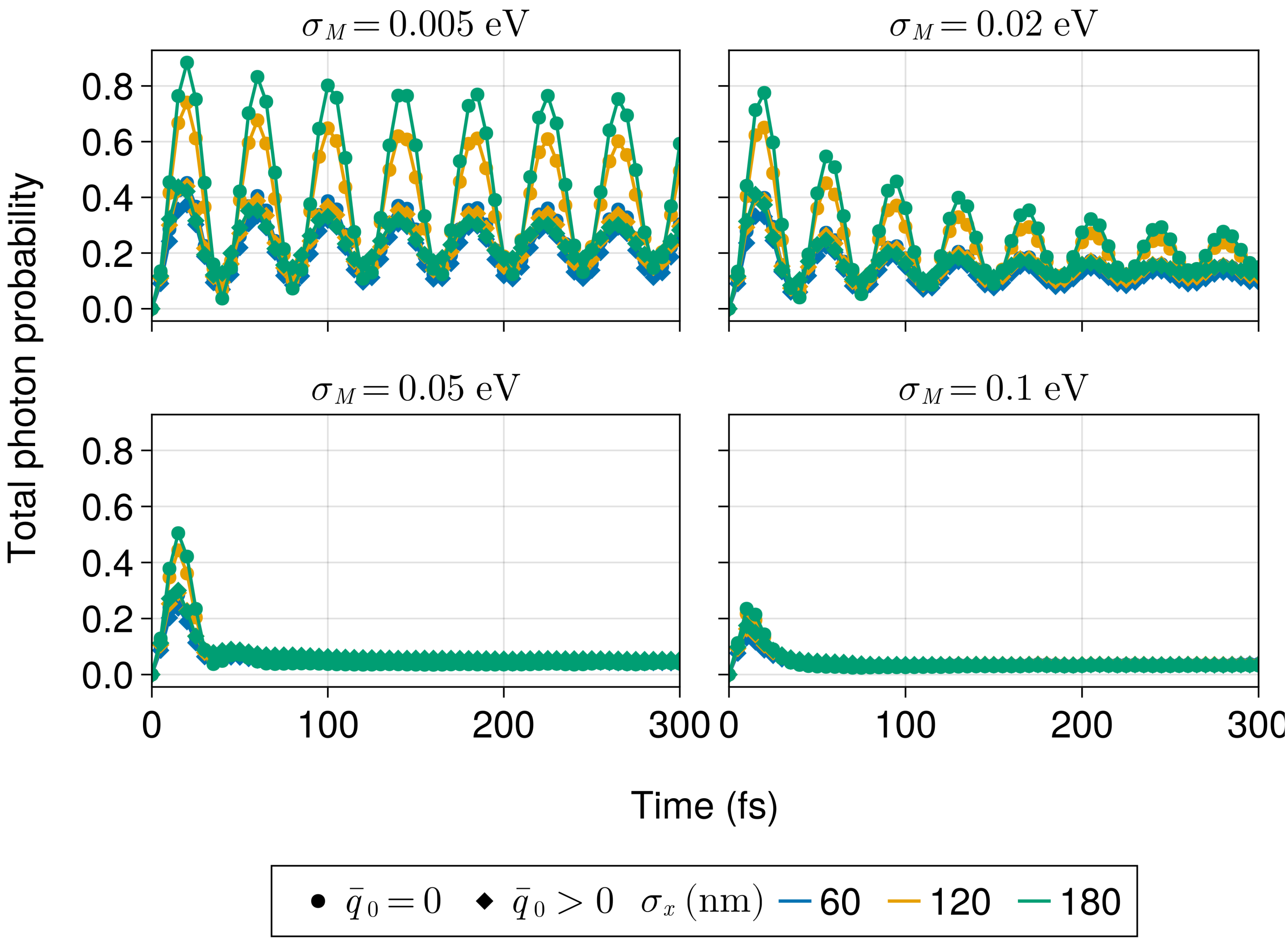

Figure S15: Total photon probability over time for several disorder magnitudes and initial states. **Parameters:** $N_M = 5000$, $N_c = 401$ ($E_{\text{cutoff}} = 3.49$ eV), $\Omega_R = 0.1$ eV, $a = 10$ nm, $E_M = 2.2$ eV.

6. Average photon probabilities

Fig. S16 shows the time-averaged photonic content of the wave packet as a function of energetic disorder for zero and negative cavity-matter detuning at multiple Rabi splittings. In all cases, the controlling factors are the energetic disorder σ_M and Ω_R. Larger disorder values decrease the photonic content of the wave packet. Conversely, stronger light-matter interactions increase the photonic probability. The redshift detuning only affects the photon probability significantly when the exciton is sufficiently broad and has zero average momentum, as seen by comparing Figs. S16 (a), (b), and (c).

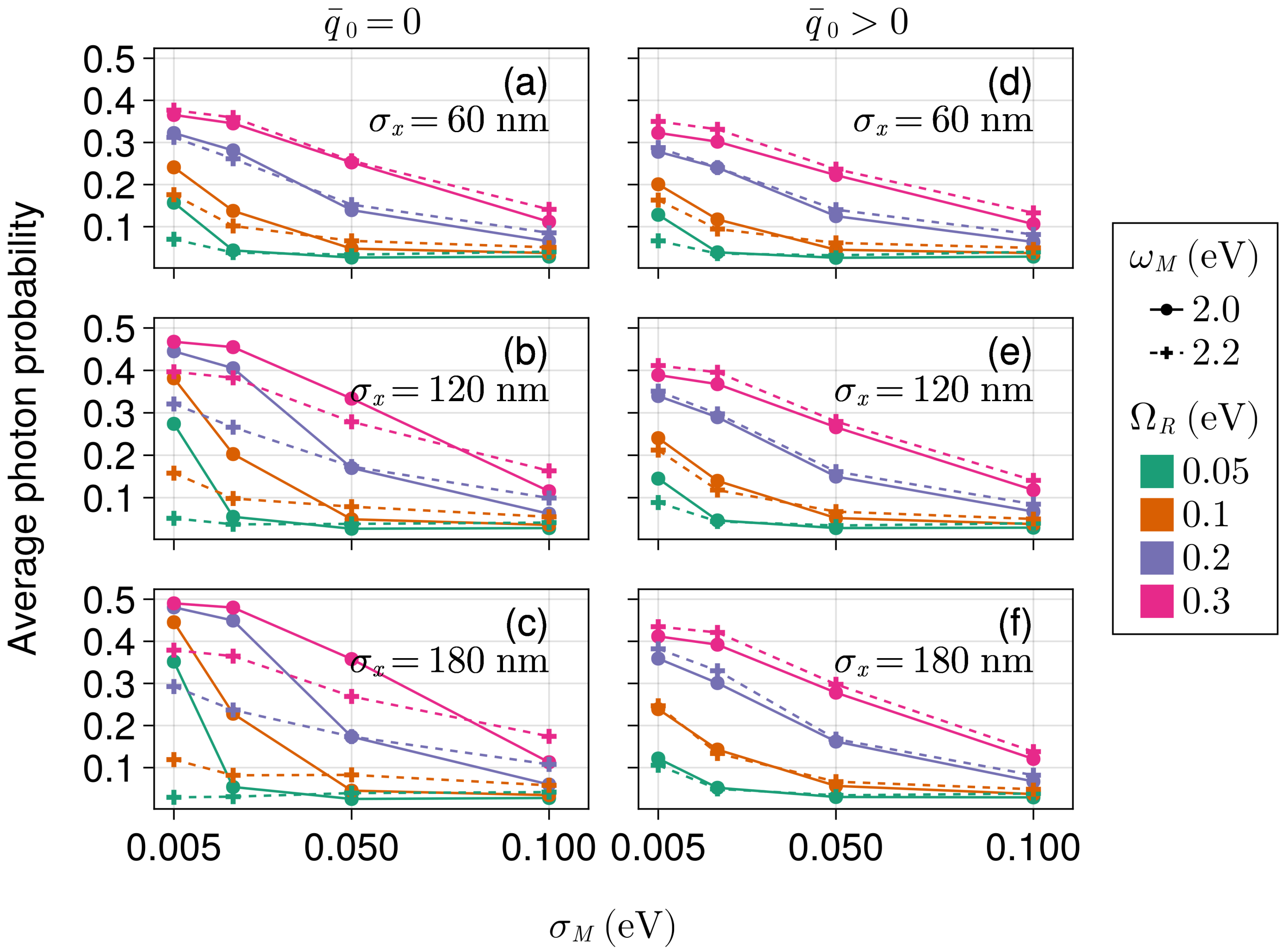

Figure S16: Average photon probability over 5 ps for a wave packet with various initial configurations for different values of Rabi splitting and detuning. **Parameters:** $N_M = 5000$, $N_c = 401$ ($E_{\text{cutoff}} = 3.49$ eV), $a = 10$ nm.

7. Detuning effect on photon weight distribution without disorder

Figs. S17 and S18 are variations of Fig. 5 using different detuning values. In Fig. S17, we examine the case where the cavity is blueshifted relative to the molecular system by 0.2 eV in the absence of disorder. In this case, there is no photon resonant with the molecular system, and the cavity modes satisfying $q = \bar{q}_0$ will necessarily be off-resonant. When $\bar{q}_0 = 0$ [Figs. S17(a)-(c)] the $q = 0$ photon is always dominant because not only it satisfies the quasimomentum matching condition, but it is also the least off-resonant mode. Thus the $q = 0$ mode maximizes $\Pi_{\bar{q}_0 L}(1 - \Pi_{\bar{q}_0 L})$. In the case where $\bar{q}_0 \neq 0$ [Figs. S17(d)-(f)] the quasimomentum matching condition arising from $e^{-\sigma_x^2 (q-\bar{q}_0)^2}$ competes with the prefactor $\Pi_{\bar{q}_0 L}(1 - \Pi_{\bar{q}_0 L})$, which is maximum for the least off-resonant modes around $q = 0$. At larger values of σ_x [Fig. S17 (f)], the exponential decay becomes fast enough to overcome the highly unfavorable prefactor, and we see the photon weight distribution peak shifting towards $\bar{q}_0$.

In Fig. S18 we examine the case where the cavity is in resonance with the molecular system. In this case, the discussion follows the arguments given in the main text. Modes near $q = 0$ provide the optimal prefactor $\Pi_{qL}(1 - \Pi_{qL})$ whereas photons with $q = \bar{q}_0$ maximize the exponential term $e^{-\sigma_x^2 (q-\bar{q}_0)^2}$. When $\bar{q}_0 = 0$, the distribution is almost trivial [Fig. S18(a)-(c)] with $q = 0$ being dominant. The fact that the width of these distributions decreases for larger σ_x is readily understood from the position-wave vector uncertainty principle. In the case where $\bar{q}_0 \neq 0$ [Fig. S18(d)-(f)], as seen from (d) to (f), the determining parameter becomes σ_x.

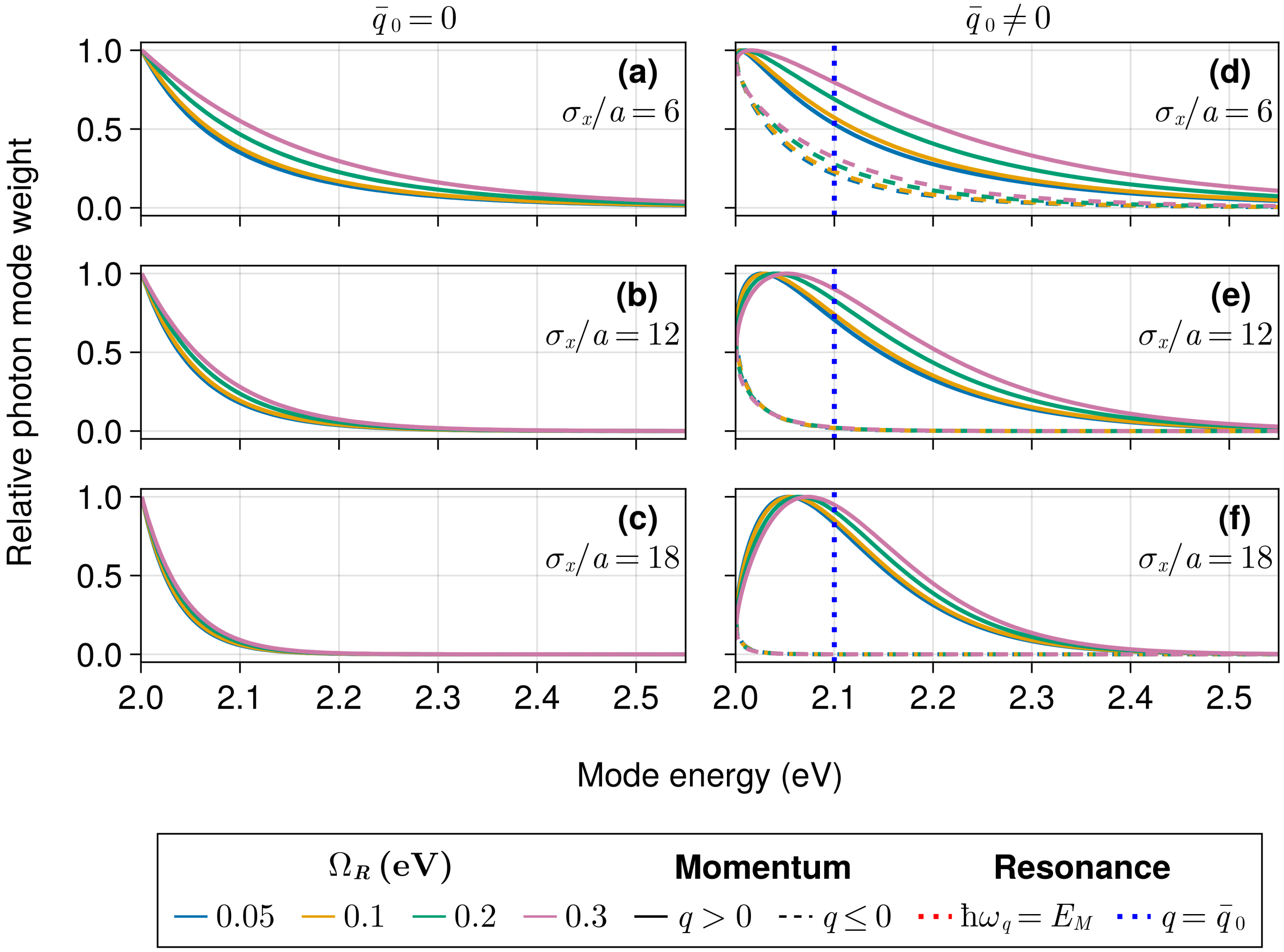

Figure S17: Cavity mode contribution under no disorder. The computation was performed over 5 ps using a 5 fs time step. Modes with $q > 0$ and $q \leq 0$ are represented by solid and dashed lines, respectively. **Parameters:** $N_M = 5000$, $N_c = 401$ ($E_{\text{cutoff}} = 3.49$ eV), $a = 10$ nm, $E_M = 1.8$ eV.

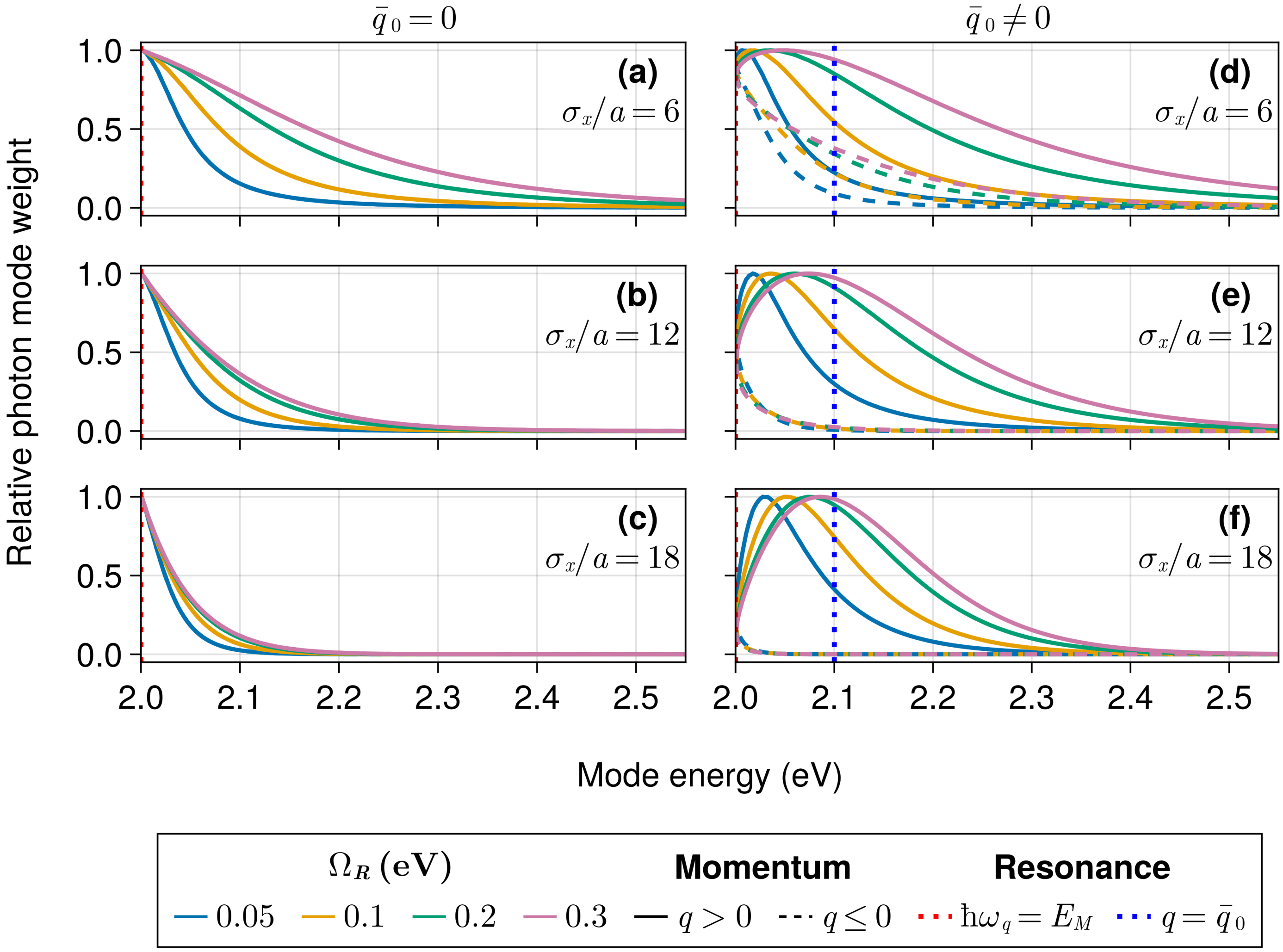

Figure S18: Cavity mode contribution under no disorder. The computation was performed over 5 ps using a 5 fs time step. Modes with $q > 0$ and $q \leq 0$ are represented by solid and dashed lines, respectively. **Parameters:** $N_M = 5000$, $N_c = 401$ ($E_{\text{cutoff}} = 3.49$ eV), $a = 10$ nm, $E_M = 2.0$ eV. No disorder.

8. Photon weight distribution in the presence of disorder

Figs. S19-S24 are variations of Fig. 5 using different parameters, including the simulation time over which the mode weights are collected.

Figs. S19(a)−(c) confirm that at weak disorder, the photon weight distributions at various values of Ω_R show minimal deviations from the zero disorder computations of Fig. 5(e). Conversely, Figs. S19(d)−(f) representing strong disorder effects on the photon weight distribution indicate a nearly flat photon weight distribution over 0.5 eV. Note the disappearance of the bias towards $q \to 0$ modes with increasing σ_M/Ω_R.

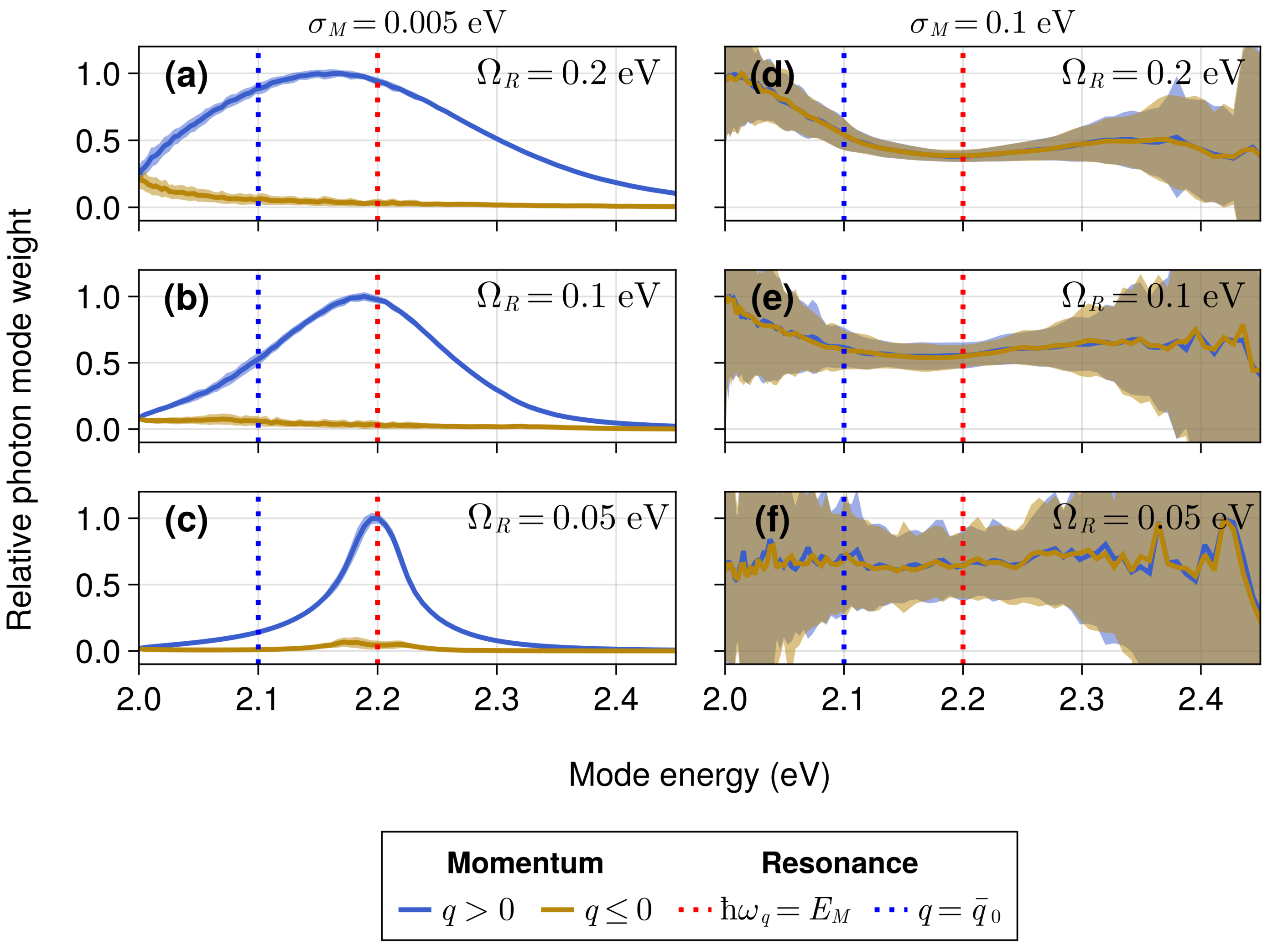

Figure S19: Cavity mode contribution measured under energetic disorder of $\sigma_M = 0.005$ eV (a, b, c) and $\sigma_M = 0.1$ eV (d, e, f). The computation was performed over 5 ps using a 5 fs time step. Band plots cover one standard deviation around the average values of 100 realizations. **Parameters:** $N_M = 5000$, $N_c = 401$ ($E_{\text{cutoff}} = 3.49$ eV), $a = 10$ nm, $\sigma_a = 1$ nm, $E_M = 2.2$ eV, $\sigma_x = 120$ nm, $\bar{q}_0 \approx 0.00565$ nm^{-1}.

Fig. S20 shows a variation of Fig. 5 using a shorter simulation time of 0.5 ps. While longer simulations

sample more events, they also collect greater uncertainty due to the stochastic nature of the wave packet evolution in the presence of disorder. Nevertheless, the qualitative profile here is in good agreement with Fig. 5, with the most noticeable difference being the generic (expected) decrease in uncertainty and, since fewer scattering events are sampled, a stronger suppression of the modes with $q < 0$ (when $\bar{q}_0 > 0$). Thus, it takes more than 500 fs for negative momentum modes to start playing a significant role in the dynamics, especially when $\sigma_M = 0.02$ eV.

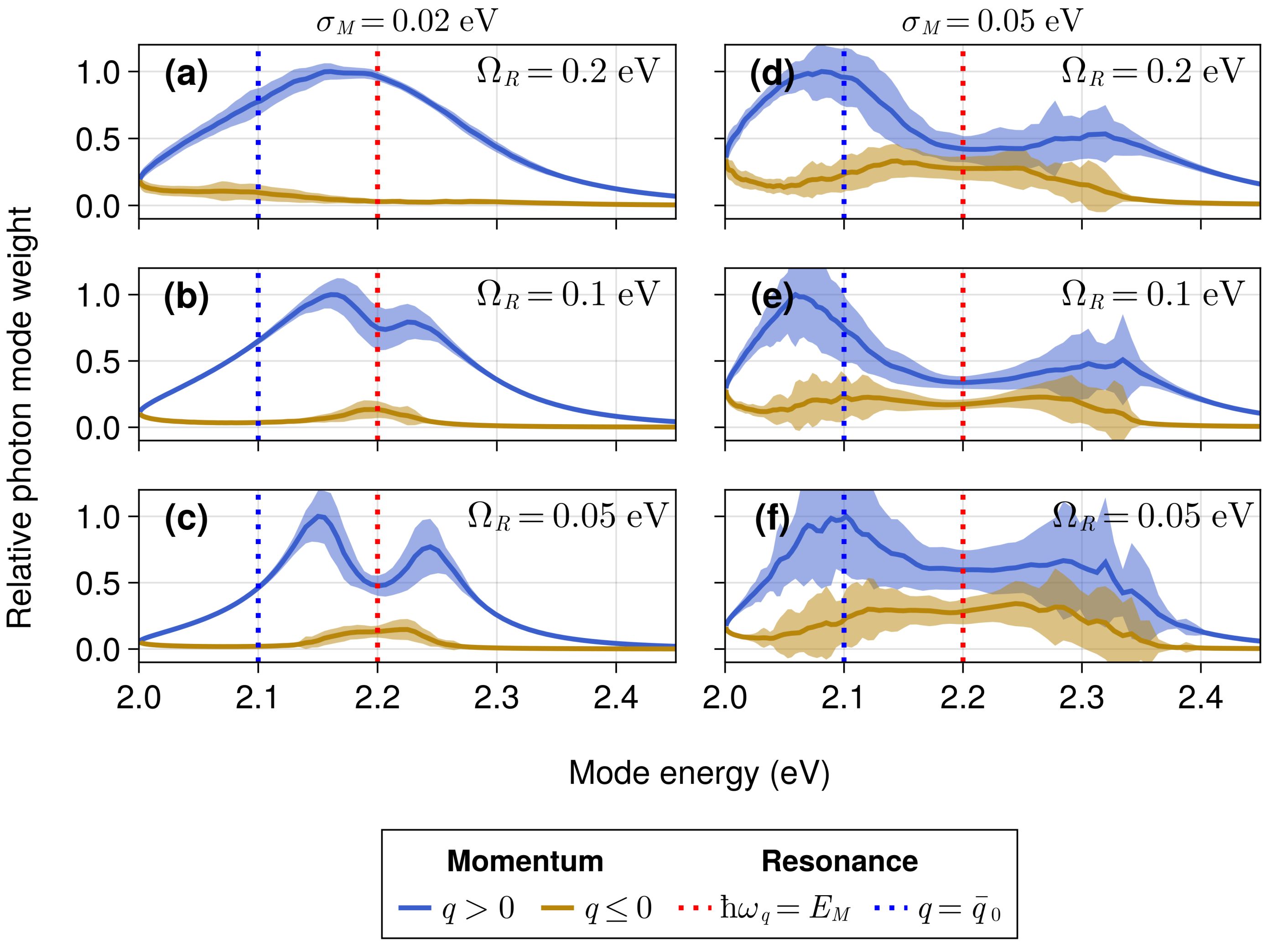

Figure S20: Cavity mode contribution measured under energetic disorder of $\sigma_M = 0.02$ eV (a, b, c) and $\sigma_M = 0.05$ eV (d, e, f). The computation was performed over 500 fs using a 5 fs time step. Band plots cover one standard deviation around the average values of 100 realizations. **Parameters:** $N_M = 5000$, $N_c = 401$ ($E_{\text{cutoff}} = 3.49$ eV), $a = 10$ nm, $\sigma_a = 1$ nm, $E_M = 2.2$ eV, $\sigma_x = 120$ nm, $\bar{q}_0 \approx 0.00565$ nm^{-1}.

Figs. S21(a)-(f) shows the mode weight distribution when $\bar{q}_0 = 0$. As expected, there is no more suppression of $q < 0$ modes. Overall the results follow similar trends to those discussed in Fig. 6.

Photon weight distributions obtained from the evolution of a narrower initial exciton wave packet ($\sigma_x = 60$ nm) are shown in Fig. S22. The trends are all in good agreement with Fig. 5, but due to

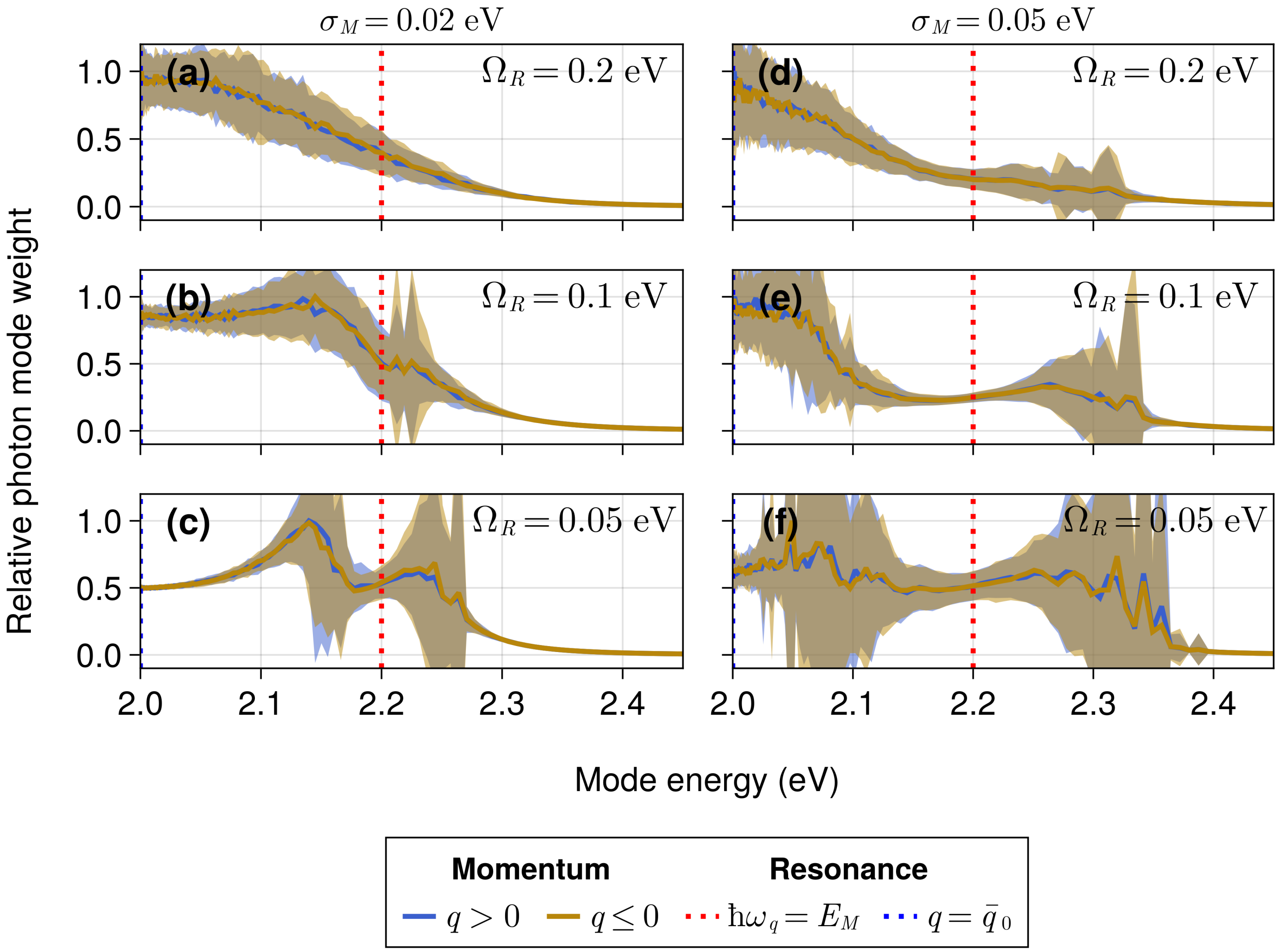

Figure S21: Cavity mode contribution measured under energetic disorder of $\sigma_M = 0.02$ eV (a, b, c) and $\sigma_M = 0.05$ eV (d, e, f). The computation was performed over 5 ps using a 5 fs time step. Band plots cover one standard deviation around the average values of 100 realizations. **Parameters:** $N_M = 5000$, $N_c = 401$ ($E_{\text{cutoff}} = 3.49$ eV), $a = 10$ nm, $\sigma_a = 1$ nm, $E_M = 2.2$ eV, $\sigma_x = 120$ nm, $\bar{q}_0 = 0$.

the weaker quasimomentum matching condition, we see that the suppression of $q < 0$ modes is generally weaker when $\sigma_M = 0.02$ eV. Conversely, when $\sigma_x = 180$ nm (Fig. S23), not only is the $q < 0$ suppression stronger, but there is an expected increase in the mode weights around $q = \bar{q}_0$.

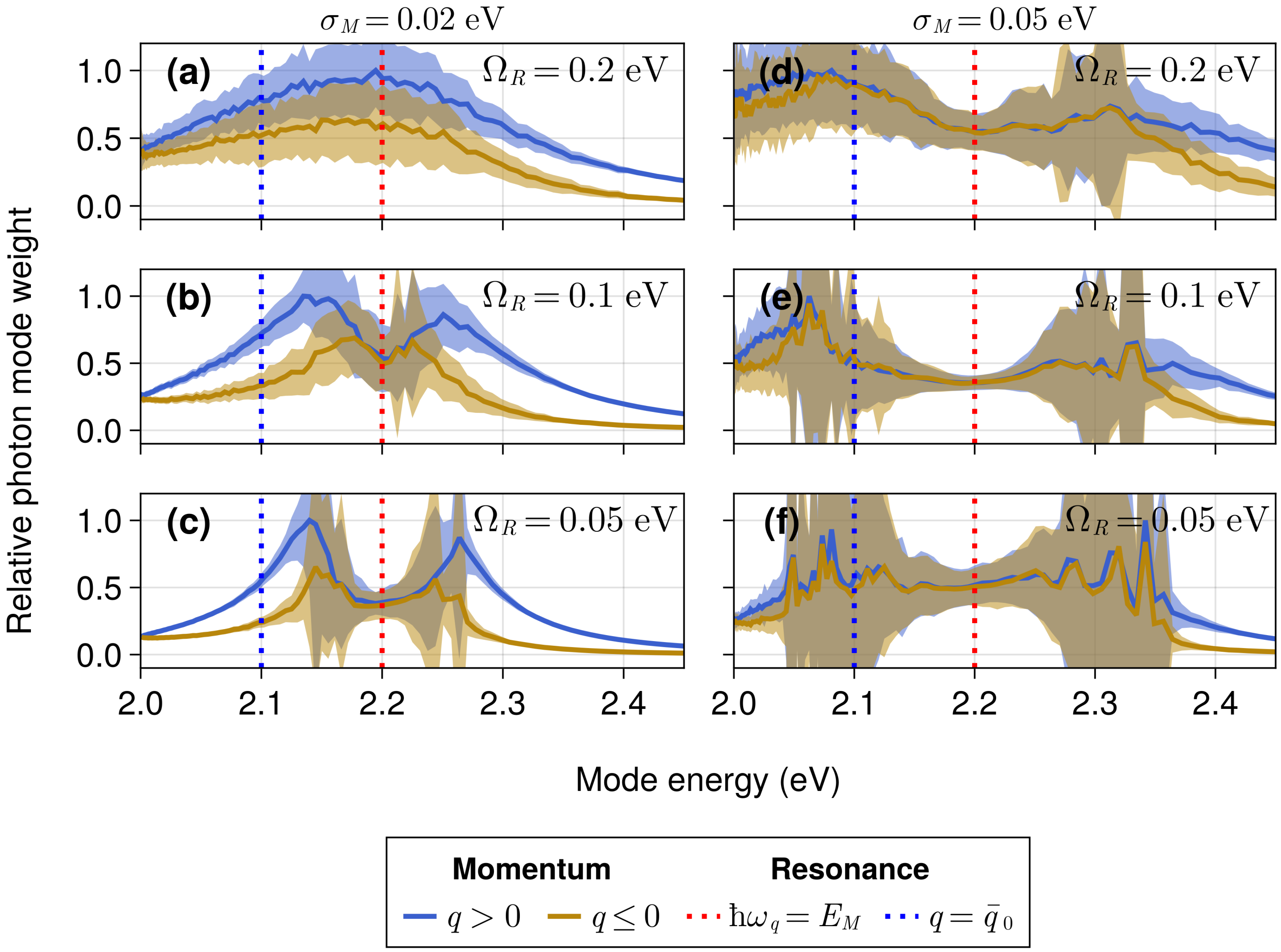

Figure S22: Cavity mode contribution measured under energetic disorder of $\sigma_M = 0.02$ eV (a, b, c) and $\sigma_M = 0.05$ eV (d, e, f). The computation was performed over 5 ps using a 5 fs time step. Band plots cover one standard deviation around the average values of 100 realizations. **Parameters:** $N_M = 5000$, $N_c = 401$ ($E_{\text{cutoff}} = 3.49$ eV), $a = 10$ nm, $\sigma_a = 1$ nm, $E_M = 2.2$ eV, $\sigma_x = 60$ nm, $\bar{q}_0 \approx 0.00565$ nm^{-1}.

Fig. S24 presents results analogous to Fig. 5, but with the cavity in resonance with the molecular system $\hbar\omega_{q=0} = E_M$. The trends seen here are in agreement with Fig. 5, but they are shifted due to the repositioning of the energy resonant region.

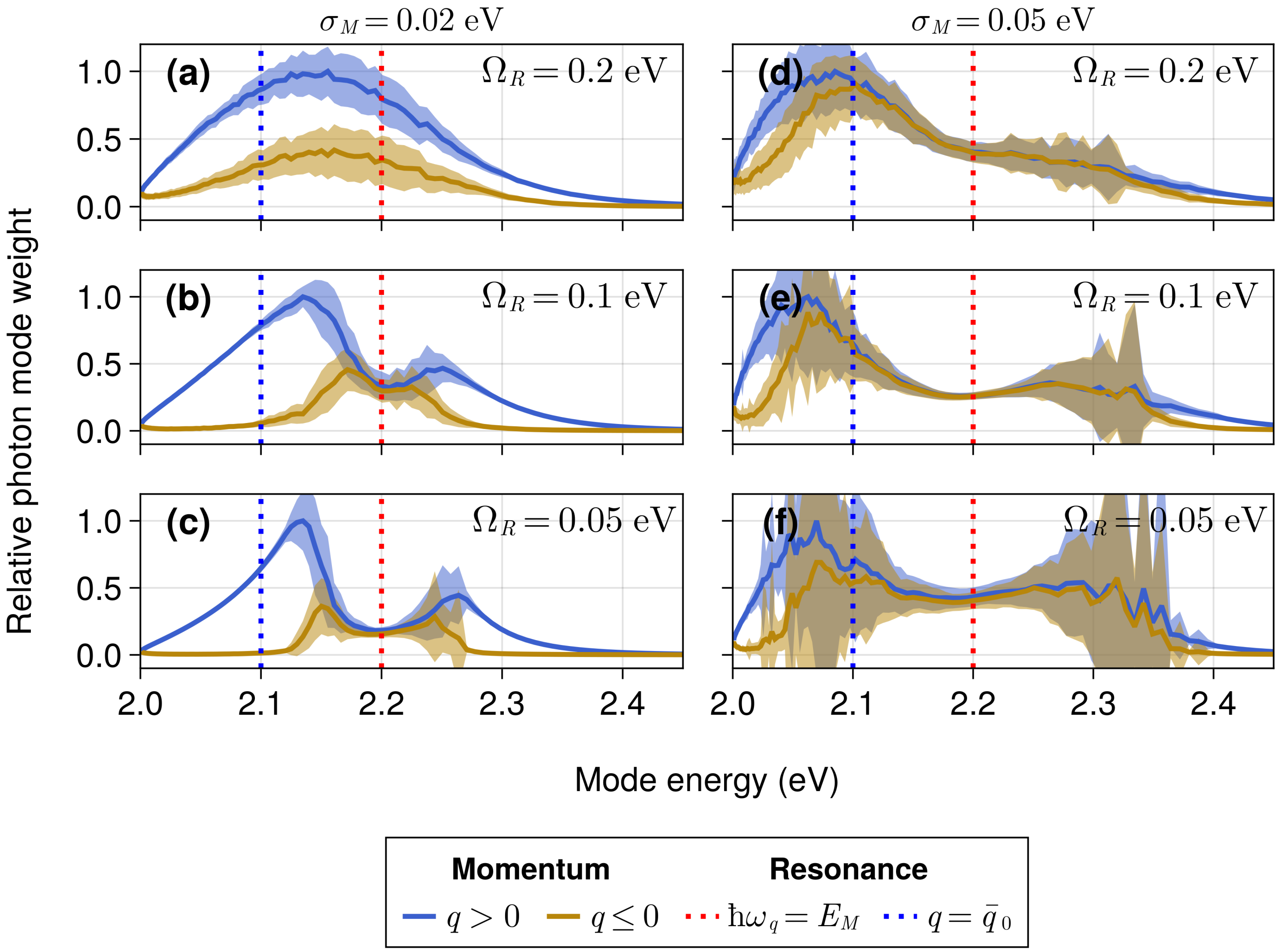

Figure S23: Cavity mode contribution measured under energetic disorder of $\sigma_M = 0.02$ eV (a, b, c) and $\sigma_M = 0.05$ eV (d, e, f). The computation was performed over 5 ps using a 5 fs time step. Band plots cover one standard deviation around the average values of 100 realizations. **Parameters:** $N_M = 5000$, $N_c = 401$ ($E_{\text{cutoff}} = 3.49$ eV), $a = 10$ nm, $\sigma_a = 1$ nm, $E_M = 2.2$ eV, $\sigma_x = 180$ nm, $\bar{q}_0 \approx 0.00565$ nm^{-1}.

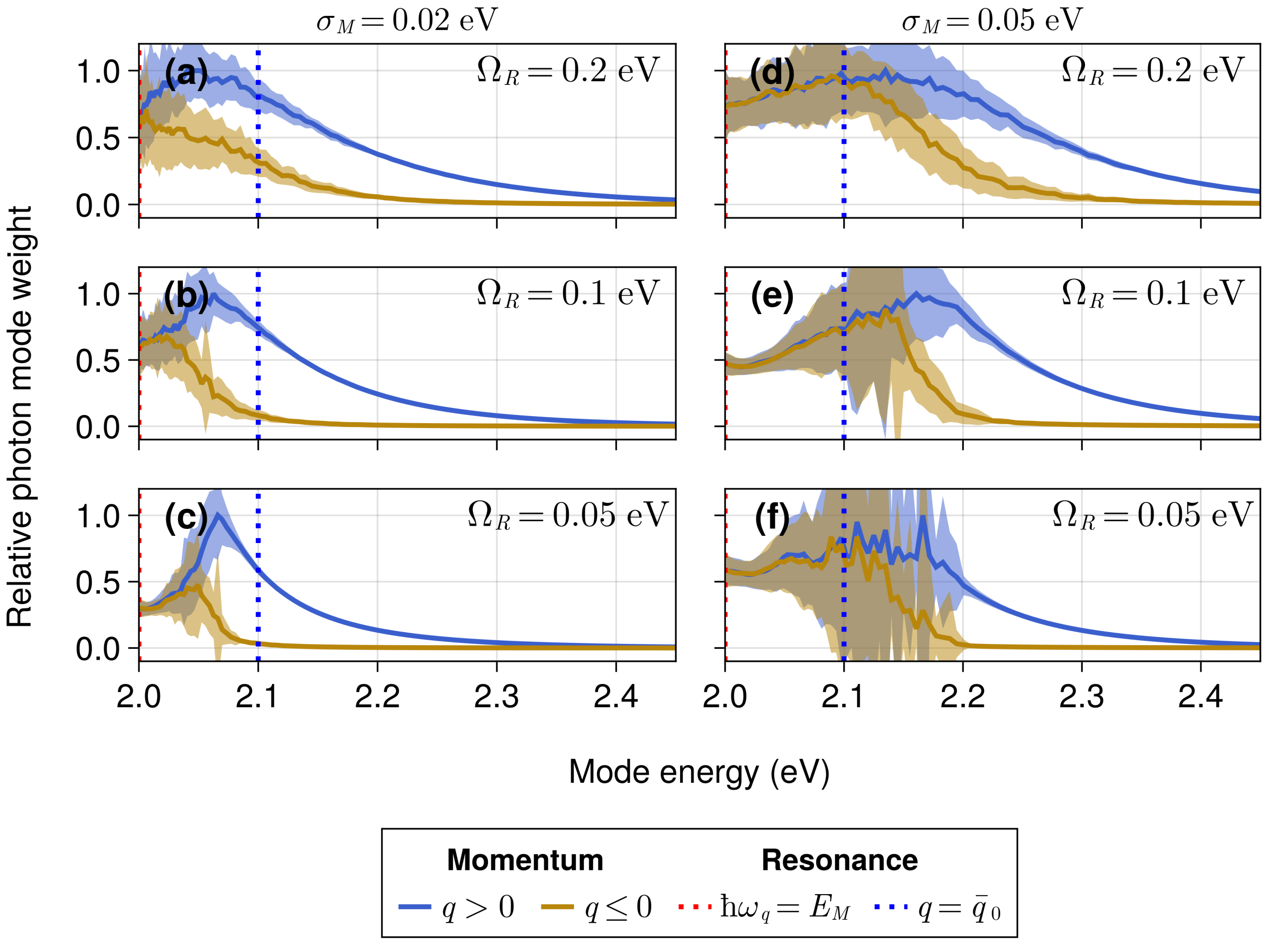

Figure S24: Cavity mode contribution measured under energetic disorder of $\sigma_M = 0.02$ eV (a, b, c) and $\sigma_M = 0.05$ eV (d, e, f). The computation was performed over 5 ps using a 5 fs time step. Band plots cover one standard deviation around the average values of 100 realizations. **Parameters:** $N_M = 5000$, $N_c = 401$ ($E_{\text{cutoff}} = 3.49$ eV), $a = 10$ nm, $\sigma_a = 1$ nm, $E_M = 2.0$ eV, $\sigma_x = 120$ nm, $\bar{q}_0 \approx 0.00565$ nm^{-1}.

9. Analytical Results for Photon weight distribution in the absence of disorder

In this section, we present the derivation of Eq. 17 of the main text. We remind the reader the basis of molecular localized states is given by $|1_n\rangle$, while (delocalized) single-photon states with all other cavity modes and molecules in the ground state will be represented by $|q\rangle$. For a generic state $|\psi(t)\rangle$ the weight of a photon $|q\rangle$ at time t is defined as

$$W(q,t) = |\langle q|\psi(t)\rangle|^2, \tag{1}$$

which can be expressed as a function of the initial state $\psi(0)$

$$W(q,t) = |\langle q| e^{-i\hat{H}t/\hbar} |\psi(0)\rangle|^2. \tag{2}$$

Introducing the resolution of the identity $\mathbb{1} = \sum_\chi |\chi\rangle\langle\chi|$ where χ are the energy eigenstates ($\hat{H}|\chi\rangle = E_\chi|\chi\rangle$) we get

$$\begin{aligned}
W(q,t) &= \left|\sum_\chi \langle q| e^{-i\hat{H}t/\hbar} |\chi\rangle \langle\chi|\psi(0)\rangle\right|^2, && (3)\\
&= \left|\sum_\chi e^{-iE_\chi t/\hbar} \langle q|\chi\rangle \langle\chi|\psi(0)\rangle\right|^2, && (4)\\
&= \left(\sum_\chi e^{-iE_\chi t/\hbar} \langle q|\chi\rangle \langle\chi|\psi(0)\rangle\right) \cdot \left(\sum_{\chi'} e^{iE_{\chi'} t/\hbar} \langle\chi'|q\rangle \langle\psi(0)|\chi'\rangle\right), && (5)\\
&= \sum_\chi |\langle q|\chi\rangle \langle\chi|\psi(0)\rangle|^2 + \sum_{\chi\neq\chi'} \langle q|\chi\rangle \langle\chi'|q\rangle \langle\psi(0)|\chi'\rangle\langle\chi|\psi(0)\rangle e^{-i(E_\chi - E'_\chi)t/\hbar}. && (6)
\end{aligned}$$

Upon time averaging, the photon weight distribution loses its time dependence as the oscillating term averages to zero. Hence,

$$\tilde{W}(q) = \lim_{T\to\infty} \frac{1}{T}\int_0^T W(q,t)dt = \sum_\chi |\langle q|\chi\rangle \langle\chi|\psi(0)\rangle|^2. \tag{7}$$

Note that $\tilde{W}(q)$ is not exactly the same quantity as shown in the main article (Eq. 16) because of the arbitrary normalization factor used there. Additionally, we compute the mode weight distribution

numerically from the time-evolved wave functions, which will inevitably introduce numerical deviations from the time-independent quantity idealized here. Nonetheless, $\tilde{W}(q)$ provides an excellent model to interpret the numerical results and, in the absence of disorder, we will show that Eq. 7 can be reduced to a simple analytical formula.

With a fixed and equal number of molecules and cavity modes ($N_M = N_c$) and in the absence of disorder ($E_n = E_M = \hbar\omega_M$ and $x_n = na$), the eigenstates of the total light-matter Hamiltonian (Eqs. 1,5,9, and 10) can be labeled by a wave vector index k and branch index $\alpha = \{\mathrm{L}, \mathrm{U}\}$, so we denote them by $|\chi\rangle \to |k\alpha\rangle$ with energy $E_{k\alpha}$. They can be written in the uncoupled basis $\{|1_n\rangle, |q\rangle\}$ as

$$|k\alpha\rangle = \sum_q \langle q|k\alpha\rangle |q\rangle + \sum_n \langle 1_n|k\alpha\rangle |1_n\rangle, \tag{8}$$

where the photonic and molecular amplitudes for each state are given by

$$\langle q|k\alpha\rangle = \delta_{kq}\frac{E_{k\alpha} - E_M}{\sqrt{(E_{k\alpha} - E_M)^2 + L_k^2}}, \tag{9}$$

$$\langle 1_n|k\alpha\rangle = \frac{-ie^{ikx_n}}{\sqrt{N_M}}\sqrt{\frac{L_k^2}{(E_{k\alpha} - E_M)^2 + L_k^2}}, \tag{10}$$

with

$$L_q = \frac{\hbar\Omega_R}{2}\sqrt{\frac{E_M}{\hbar\omega_q}}. \tag{11}$$

We can compute the total molecular contribution to the eigenstate $|k\alpha\rangle$ as

$$\Pi_{k\alpha} = \sum_n |\langle 1_n|k\alpha\rangle|^2 = \frac{1}{N_M}\sum_n \frac{L_k^2}{(E_{k\alpha} - E_M)^2 + L_k^2} = \frac{L_k^2}{(E_{k\alpha} - E_M)^2 + L_k^2}. \tag{12}$$

Hence, Eq. 10 can be expressed more compactly as

$$\langle 1_n|k\alpha\rangle = -ie^{ikx_n}\sqrt{\frac{\Pi_{k\alpha}}{N_M}}. \tag{13}$$

As only one photon state contributes to each eigenstate $|k\alpha\rangle$ (δ_{qk} in Eq. 9) we can also simplify the

photonic amplitude to

$$\langle q|k\alpha\rangle = \delta_{kq}\sqrt{(1-\Pi_{k\alpha})}. \tag{14}$$

One important observation that we will use later is that at resonance, i.e. $E_M = \hbar\omega_q$, the energy gap between the bare molecular system and each polariton branch $\alpha = \{L, U\}$ becomes $E_M - E_{kL} = E_{kU} - E_M = \Omega_R/2$. Thus, at resonance, it follows from Eq. 12 that $\Pi_{q\alpha} = 0.5$.

We can use the result from Eq. 14 in Eq. 7 to compute the mode weight distribution in the absence of disorder:

$$\tilde{W}(q) = \sum_{k\alpha} |\langle q|k\alpha\rangle \langle k\alpha|\psi(0)\rangle|^2, \tag{15}$$

$$= \sum_{k\alpha} |\delta_{kq}\sqrt{(1-\Pi_{k\alpha})}\langle k\alpha|\psi(0)\rangle|^2, \tag{16}$$

$$= \sum_{\alpha}(1-\Pi_{q\alpha})|\langle q\alpha|\psi(0)\rangle|^2. \tag{17}$$

As defined in the text, the initial exciton is created as a Gaussian wave packet with spacial spread σ_x and average momentum $\bar{q}_0$

$$|\psi(0)\rangle = \sqrt{a}(2\pi\sigma_x^2)^{-1/2}\sum_n e^{-(x_n-\bar{x}_0)^2/2\sigma_x^2}e^{i\bar{q}_0 x_n}|1_n\rangle, \tag{18}$$

where $\sqrt{a}(2\pi\sigma_x^2)^{-1/2}$ is a normalization constant. The $\sqrt{a}$ prefactor is necessary for the molecular probability distribution to be normalized to one in the continuum limit. Using Eq. 13 we get

$$\langle q\alpha|\psi(0)\rangle = \sqrt{a}(2\pi\sigma_x^2)^{-1/2}\sum_n e^{-(x_n-\bar{x}_0)^2/2\sigma_x^2}e^{i\bar{q}_0 x_n}\langle q\alpha|1_n\rangle, \tag{19}$$

$$= i\sqrt{a}(2\pi\sigma_x^2)^{-1/2}\sum_n e^{-(x_n-\bar{x}_0)^2/2\sigma_x^2}e^{i\bar{q}_0 x_n}e^{-iqx_n}\sqrt{\frac{\Pi_{q\alpha}}{N_M}}. \tag{20}$$

For small enough distances between the molecules relative to $1/q$ such that $qa \ll 1$ for any relevant q, the summation above can be approximated by:

$$\langle q\alpha|\psi(0)\rangle \approx i\sqrt{a}(2\pi\sigma_x^2)^{-1/2}\sqrt{\frac{\Pi_{q\alpha}}{N_M}}\frac{1}{a}\int_{-\infty}^{+\infty} e^{-(x-\bar{x}_0)^2/2\sigma_x^2}e^{-i(q-\bar{q}_0)x}dx\,. \tag{21}$$

This integral is a Fourier transform from position to momentum space; it can be evaluated to

$$\langle q\alpha|\psi(0)\rangle \approx i\sqrt{a}(2\pi\sigma_x^2)^{-1/2}\sqrt{\frac{\Pi_{q\alpha}}{N_M}}\frac{(2\pi\sigma_x^2)^{1/2}}{a}e^{-\sigma_x^2(q-\bar{q}_0)^2/2}. \tag{22}$$

$$\tag{23}$$

Therefore,

$$|\langle q\alpha|\psi(0)\rangle|^2 \approx \frac{\Pi_{q\alpha}}{N_M a}e^{-\sigma_x^2(q-\bar{q}_0)^2}. \tag{24}$$

Finally, Eq. 17 becomes

$$\tilde{W}(q) = \sum_\alpha \frac{\Pi_{q\alpha}(1-\Pi_{q\alpha})}{N_M a}e^{-\sigma_x^2(q-\bar{q}_0)^2}, \tag{25}$$

$$= \frac{1}{N_M a}e^{-\sigma_x^2(q-\bar{q}_0)^2}\left[\Pi_{qL}(1-\Pi_{qL}) + \Pi_{qU}(1-\Pi_{qU})\right], \tag{26}$$

Since $\Pi_{qU} + \Pi_{qL} = 1$ we have the final expression

$$\tilde{W}(q) = \frac{2}{N_M a}\Pi_{qL}(1-\Pi_{qL})e^{-\sigma_x^2(q-\bar{q}_0)^2}. \tag{27}$$

As discussed above, at resonance $\Pi_{qL} = 0.5$ maximizing the prefactor $\Pi_{qL}(1-\Pi_{qL})$. At the same time the exponential factor $e^{-\sigma_x^2(q-\bar{q}_0)^2}$ vanishes when q is too far from $\bar{q}_0$. These two effects are used to explain the results in the main text.
