# Supplementary material for: Theoretical Analysis of Exciton Wave Packet Dynamics in Polaritonic Wires
Source: J Phys Chem Lett. 2023 Jun 14;14(24):5681–91. doi: 10.1021/acs.jpclett.3c01082 (PMC10291640; doi:10.1021/acs.jpclett.3c01082)
Supplement: Supplementary file 2 — jz3c01082_si_002.pdf [file jz3c01082_si_002.pdf]

jz-2023-01082p.R1

Name: Peer Review Information for "Theoretical Analysis of Exciton Wave Packet Dynamics in Polaritonic Wires"

First Round of Reviewer Comments

Reviewer: 1

Comments to the Author

In this submission the authors present a study of exciton wave packet evolution in disordered lossless polaritonic wires. They investigate different conditions of weak to strong energetic disorder for the time-resolved exciton dynamics and show ballistic, diffusive and subdiffusive polariton-assisted exciton transport. They study these conditions with respect to different parameters such as cavity length (i.e. small number of molecules can reproduce only early dynamics, caution is needed when the wave packet spreads over the length scale of system size) and number of photon modes (i.e. a multi-mode description with large energy interval is needed to describe the system evolution). Overall, this is an interesting and especially very thoroughly executed analysis of space-time-resolved exciton wave packet evolution in polaritonic wires. This paper is suitable for the scope of The Journal of Physical Chemistry Letters and I am in favor of publishing this work after the authors addressed the following remarks.

Remarks:

- My main remark is that, although I of course appreciate the details and the overall very thoroughly executed analysis, this manuscripts lacks sometimes a clear picture or goal. If the authors could make their goal (e.g. why investigating these parameters?, how is that relevant for future theoretical and experimental investigations?, how does that go beyond past studies?) clearer in their subsections, it would benefit the manuscript greatly and make it much more appealing for a broader audience. For example a sentence like '… relevant to future theoretical and experiment investigations of polaritonic chemistry' in the conclusion would be much stronger with some concrete examples.

- On page 10 the authors use photon mode and photon number interchangeable. To my understanding N_c is the number of photon modes. Do the authors consider an explicit truncation of the Fock-space i.e. each mode can include only n number of photons? A clarification would be greatly appreciated.

- In Figure 2, the authors mention that the radiation field was modeled using 1601 modes and a fixed Rabi-splitting of 0.1eV. However, given they investigate different numbers of molecules (or length of the cavity) the coupling strength also changes. Should that not also change the Rabi-splitting? Can the authors clarify why different cavity lengths/number of molecules still induces the same Rabi-splitting?

- Given that this is a very quantitative study, and especially because it includes a multi-mode picture with many off-resonant modes to the two level system, can the authors comment on, if the results

would still hold if one would include more than just a two level system i.e. the off-resonant modes could couple to higher energies?

- Additionally as the authors explicitly choose to call their matter systems molecular subsystems (and not atomic), I was wondering which concrete experiments (or molecular systems) they had in mind that are sufficiently represented by their 2-level molecular picture? More detail on that could strengthen the paper a lot and generalize its applicability.

- Can the authors comment on, if the dipole approximation (i.e. the transition dipole does not need a spatial resolution) for the highest frequencies included in this multi-mode picture is still valid? (Given up to 1601 modes are used).

- Very minor, but on page 16 in “In this subsection, we work with …” there is a double sigma_x.

Reviewer: 2

Comments to the Author

Aroeira et al. present a theoretical study of exciton transport of a an ensemble of two-level systems in a nano-cavity. A particular nice point is that the authors look at a Fabry-Perot type cavity that allows for periodic boundary conditions of at least one dimension. This is something that is not done in most theoretical studies, but actually matches experimental conditions. I think that the paper is a timely contribution that nicely complements the theoretical understanding of how to model ensembles under strong light-matter coupling. However, one point of criticism is the the presentation as a "molecular" ensemble. Actual molecules can not be accurately modelled as two-level systems as they are also subject to vibrational excitations and static dipole moments (which may cause additional effects in an ensemble). I recommend to change the wording from molecules to two-level systems. This would not lower the value of the study in my point of view but rather clarify the approximations involved.

Author's Response to Peer Review Comments:

May 29, 2023

Referee response for "Theoretical Analysis of Exciton Wave Packet Dynamics in Polaritonic Wires"

We thank both referees for their insightful comments, suggestions, and pertinent questions. Our response to each item is given below in blue and examples of changes to the main manuscript are provided in *italics*.

Reviewer 1.

"My main remark is that, although I of course appreciate the details and the overall very thoroughly executed analysis, this manuscripts lacks sometimes a clear picture or goal. If the authors could make their goal (e.g. why investigating these parameters?, how is that relevant for future theoretical and experimental investigations?, how does that go beyond past studies?) clearer in their subsections, it would benefit the manuscript greatly and make it much more appealing for a broader audience. For example, a sentence like '... relevant to future theoretical and experiment investigations of polaritonic chemistry' in the conclusion would be much stronger with some concrete examples."

We thank the referee for this suggestion. We have extended or modified several sentences throughout the manuscript to emphasize the goal of each presented result. These changes are highlighted in the marked-up version of the manuscript submitted. Some examples are listed below.

Page 13.

This discussion is important for both theoretical models and the interpretation of experimental results since, as we will show, qualitative incorrect results are obtained when too few photon modes are considered

Page 14.

Note that the error vanishes for a sufficiently large number of photons modes, therefore verifying the convergence of our model. Recent work has shown that this is not always guaranteed. Mandal et. al. reported that using the dipole gauge and RWA, polaritonic dispersion relations do not converge with respect to the number of cavity modes unless the often neglected dipole self-energy terms are included in the model.

Page 14.

Next, we will consider how static fluctuations in the energy gaps of the two-level systems change the results presented in Fig. 3

Page 15.

In the results discussed so far in this letter, we found that quantitative converged results require many photon modes. This indicates that a broad energy range of photons contributes to exciton propagation, which is at odds with the intuition that resonant processes must dominate the dynamics over sufficiently long times. To further understand these findings, we inspect the photonic composition of the wavepacket over time. This will give us insight into the state of the radiation within the cavity which is inaccessible by single-mode models. Moreover, we will discuss how this photonic profile changes with experimentally controllable variables. To obtain this profile, we compute the wave packet via the time-averaged relative mode weight distribution, defined as follows

Page 24.

The results presented here probe the transient dynamics of exciton transport, which differs from previous studies that focused on steady-state properties

"On page 10 the authors use photon mode and photon number interchangeably. To my understanding N_c is the number of photon modes. Do the authors consider an explicit truncation of the Fock-space i.e. each mode can include only n number of photons? A clarification would be greatly appreciated."

We thank the referee for finding this issue. Indeed, the usage of the term "photon number" was not precise in that instance and has been corrected, as we reformulated the sentence mentioned by the referee. We work within the one-excitation manifold, i.e., the Fock space is truncated such that all states contain either 0 or 1 photon. Hence, the dynamics involve only one-photon processes. We included a new sentence in the manuscript (pg. 9) reinforcing this characteristic of our model:

We investigate the dynamics within the one-excitation manifold, i.e., the Fock-space is truncated to include photon modes with either 0 or 1 photons.

"In Figure 2, the authors mention that the radiation field was modeled using 1601 modes and a fixed Rabi-splitting of 0.1 eV. However, given they investigate different numbers of molecules (or length of the cavity) the coupling strength also changes. Should that not also change the Rabi-splitting? Can the authors clarify why different cavity lengths/number of molecules still induces the same Rabi-splitting?"

The Rabi splitting is often expressed as $\Omega_R = g\sqrt{N_M}$ where g is the single-molecule light-matter coupling at resonance. Hence, as noted by the reviewer, increasing the number of sites (molecules, N_M) at fixed volume will increase the Rabi splitting. However, the individual coupling strength g also depends on the quantization volume, e.g., $g = \mu \left(\frac{\hbar\omega}{2\epsilon_0 V}\right)^{1/2}$. Therefore, we can write $\Omega_R = \mu\sqrt{\hbar\omega\rho/2\epsilon_0}$ where ρ is the density of the system ($\frac{N_M}{L_x L_y L_z}$). Since in our finite-size study, we increase both N_M and L_x simultaneously ($L_x = N_M a$, where a is the mean intersite distance), the density of the system remains constant, and thus we are able to maintain the same Rabi splitting for all different-sized simulations. We have made changes to the manuscript making this aspect of the model more explicit (pg. 11).

The results presented in Fig. 2 were obtained by varying the number of sites (N_M) and consequently the wire length ($Lx = N_M a$). This scheme maintains the density of the system constant allowing us to fix the Rabi splitting at 0.1 eV.

"Given that this is a very quantitative study, and especially because it includes a multi-mode picture with many off-resonant modes to the two level system, can the authors comment on, if the results would still hold if one would include more than just a two level system i.e. the off-resonant modes could couple to higher energies?"

If the material system under study presented two or more bright low-lying excited states, the dynamics could be significantly changed. However, in the case where two bright excited states are separated by an energy greater than twice the Rabi splitting, we believe our results would still hold. Consider, for example, a collection of three-level systems ($\{\varepsilon_0, \varepsilon_1, \varepsilon_2\}$). Our initial state is an exciton superposition of ε_1 states. The $\{\varepsilon_2\}$ states can only be accessed via an emission of photon $\varepsilon_1 \to \varepsilon_0$ followed by an absorption event $\varepsilon_0 \to \varepsilon_2$. If the photons emitted by $\{\varepsilon_1\}$ are highly off-resonant with those which efficiently induce the $\varepsilon_0 \to \varepsilon_2$ transition, then the introduction of ε_2 states is highly unlikely to affect the dynamics of the lower lying $\{\varepsilon_1\}$. As a numerical example, let us consider $E_{0\to1} = 2.0$ eV (as in most of our results), and $E_{0\to2} = 3.0$ eV. From Fig. 5, we see that the probability of finding photons with energy greater than 2.5 eV is generally very small. Hence, another excited state centered around 3.0 eV would have very limited participation in the overall process. We added a comment to the main manuscript addressing this point (pg. 19)

The results presented in this section also allow us to evaluate the validity of our two-level approximation for matter. Consider for example a three-level system with well-separated transition energies $E_{0\to1} = 2.0$ eV and $E_{0\to2} = 3.0$ eV. From Fig. 6, we see that the probability of finding photons with energy greater than 2.5 eV is generally very small. Hence, another excited state centered around 3.0 eV would not interact significantly with any of the populated photon modes and, therefore, would have very limited participation in the overall process.

"Additionally, as the authors explicitly choose to call their matter systems molecular subsystems (and not atomic), I was wondering which concrete experiments (or molecular systems) they had in mind that are sufficiently represented by their 2-level molecular picture? More detail on that could strengthen the paper a lot and generalize its applicability."

This was also pointed out by reviewer 2 and made appropriate changes to the terminology were made to more rigorously reflect the approximations employed in this work. Still, we believe that, while the model used here is more suitable for atomic resonators, it can also be used to study molecules over ultrafast timescales, especially if their vibronic coupling is weak. To clarify this assumption, we added the following sentence to the theory part (pg. 6):

Matter is modeled by a chain of two-level systems, which can represent atoms or molecules with weak vibronic coupling over ultrafast times.

"Can the authors comment on if the dipole approximation (i.e., the transition dipole does not need a spatial resolution) for the highest frequencies included in this multi-mode picture is still valid? (Given up to 1601 modes are used)."

In the computation with the largest number of photon modes ($N_c = 1601$), the maximum value of k is approximately 0.1 nm^{-1}, therefore corresponding to a wavelength of roughly 62 nm, which is six times greater than the average distance a between sites, thus making the electric dipole approximation valid. Moreover, as we concluded in this work, highly off-resonant modes (e.g., modes with energy greater than 3.0 eV) give a negligible contribution to the overall dynamics, and we believe that the inclusion of corrections to the electrical dipole approximation would not overcome the energetic suppression of highly off-resonant photon modes. This important observation is included in the revised manuscript (pg. 9):

Since this Hamiltonian relies on the electric dipole approximation, we numerically verified that even when very high-energy photons are considered, the wavelength remains greater than the spacing between sites. Furthermore, as our results will show, these highly off-resonant modes are negligible to the exciton transport.

"Very minor, but on page 16 in "In this subsection, we work with " there is a double sigma_x."

We thank the reviewer for pointing that out. This typo has been corrected.

Reviewer 2.

"However, one point of criticism is the presentation as a "molecular" ensemble. Actual molecules can not be accurately modeled as two-level systems as they are also subject to vibrational excitations and static dipole moments (which may cause additional effects in an ensemble). I recommend to change the wording from molecules to two-level systems. This would not lower the value of the study in my point of view but rather clarify the approximations involved."

We thank the reviewer for a thorough reading of our manuscript. We agree with the reviewer's suggestions and have changed various instances of "molecular" or "molecule" to "two-level systems", "sites", or "atomic resonators" when more appropriate. See also our response to Reviewer 1 addressing the same point for the included change in the manuscript.

We hope that this additional content satisfactorily addresses the comments from the reviewers. Thank you for your time and consideration.

jz-2023-01082p.R2

Name: Peer Review Information for "Theoretical Analysis of Exciton Wave Packet Dynamics in Polaritonic Wires"

Second Round of Reviewer Comments

Reviewer: 1

Comments to the Author

I thank the authors for addressing all my comments and I am in favor of publishing this work.

Author's Response to Peer Review Comments:

The supporting information statement has been corrected as requested by the editor.
